# Physicochemical Drivers of Microbial Community Structure in Sediments of Lake Hazen, Nunavut, Canada

**DOI:** 10.3389/fmicb.2018.01138

**Published:** 2018-06-05

**Authors:** Matti O. Ruuskanen, Kyra A. St. Pierre, Vincent L. St. Louis, Stéphane Aris-Brosou, Alexandre J. Poulain

**Affiliations:** ^1^Department of Biology, University of Ottawa, Ottawa, ON, Canada; ^2^Department of Biological Sciences, University of Alberta, Edmonton, AB, Canada; ^3^Department of Mathematics and Statistics, University of Ottawa, Ottawa, ON, Canada

**Keywords:** microbial diversity, microbial community composition, arctic lakes, lake sediments, high-throughput sequencing, machine learning

## Abstract

The Arctic is undergoing rapid environmental change, potentially affecting the physicochemical constraints of microbial communities that play a large role in both carbon and nutrient cycling in lacustrine environments. However, the microbial communities in such Arctic environments have seldom been studied, and the drivers of their composition are poorly characterized. To address these gaps, we surveyed the biologically active surface sediments in Lake Hazen, the largest lake by volume north of the Arctic Circle, and a small lake and shoreline pond in its watershed. High-throughput amplicon sequencing of the 16S rRNA gene uncovered a community dominated by Proteobacteria, Bacteroidetes, and Chloroflexi, similar to those found in other cold and oligotrophic lake sediments. We also show that the microbial community structure in this Arctic polar desert is shaped by pH and redox gradients. This study lays the groundwork for predicting how sediment microbial communities in the Arctic could respond as climate change proceeds to alter their physicochemical constraints.

## Introduction

While human-induced climate change is a global reality, its effects are amplified in the Arctic, severely impacting freshwater ecosystems there. Indeed, increases in air temperature and precipitation lead to enhanced glacial melt and runoff (Bliss et al., 2014), permafrost thaw (Mueller et al., 2009), and a reduction in ice-cover duration (Vincent and Laybourn-Parry, 2008). In response to these changes, High Arctic lakes can undergo shifts in their temperature, light and nutrient availability, pH, and salinity (Mueller et al., 2009; Lehnherr et al., 2018). Changes in these abiotic factors can be expected to influence the structure of microbial communities which, in turn, can then affect their physicochemical environment, for example through nitrogen fixation, organic carbon mineralization, or sulfate reduction. However, the microbial communities inhabiting polar lake sediments are still poorly characterized, and what drives community composition is relatively unknown. Although over the past few years several studies taking place in the polar regions have used next-generation sequencing to characterize microbial communities (Stoeva et al., 2014; Emerson et al., 2015; Hauptmann et al., 2016; Schütte et al., 2016; Wang et al., 2016; Mohit et al., 2017; Thaler et al., 2017), data on sediment microbial communities in these environments is still sparse. The available data are also biased toward small lakes and thaw ponds, thus underrepresenting large arctic lakes.

To predict how environmental changes might impact future freshwater quality and productivity in the Arctic, we first need to understand the structure of the microbial communities that are mediating the biogeochemical cycles in these environments. This is usually achieved by PCR amplicon sequencing of the 16S rRNA gene, which is commonly used as a phylogenetic marker gene for bacteria and archaea. To move beyond the structural description of a microbial community, we need to understand (i) how the environment is shaping a community, and (ii) how a community, in turn, shapes its environment. Metrics describing microbial community structure can be correlated with physicochemical variables using multivariate methods, such as Non-metric MultiDimensional Scaling (NMDS), (un-)constrained correspondence analysis, or cluster analysis (Buttigieg and Ramette, 2014). However, most of these approaches remain descriptive, and assume that the relationships between community composition and abiotic factors are linear. To address these limitations, machine learning methods have been used, for instance to predict disease progression from human gut microbiomes (Pasolli et al., 2016), to determine the factors affecting microbial diversity in soil (Ge et al., 2008), or to show that pH controls microbial diversity in acid mine drainage (Kuang et al., 2013). More recently, Beall et al. (2016) identified Operational Taxonomic Units (OTUs) with different abundances between high and low ice conditions in lakes, while Sun et al. (2017) predicted that very low levels of antimony [Sb(V)] and arsenic [As(V)] increase microbial diversity in soils. However, such machine learning approaches have not yet been used to characterize the drivers of microbial diversity in Arctic freshwater environments. Without a full metagenomics or metatranscriptomics dataset, it is difficult to properly describe a functional link between community structure and function. When such data are unavailable, studies have suggested that amplicon-based sequencing data be used to make limited functional predictions of environmental microbial communities (Louca et al., 2016b). This type of functional prediction relies on the presence of taxa known to participate in well characterized biological processes or functions (e.g., oxygenic photoautotrophy, sulfate reduction, and methanogenesis; Langille et al., 2013; Aßhauer et al., 2015; Louca et al., 2016b) but has yet to be applied to undersampled and / or extreme environments such as high arctic lake sediments.

Here, we characterized over a period of two years the microbial community structure in sediments collected from freshwater systems in the Lake Hazen watershed, located in Quttinirpaaq National Park on northern Ellesmere Island, in Nunavut, Canada (82°N, 71°W; Figure S1). Bacterial and archaeal 16S rRNA gene amplicon sequencing from environmental DNA samples allowed us to characterize the microbial communities across space and time. Taking advantage of recent developments in machine learning, we determined the physicochemical drivers of the community structures, and use functional mapping of the community structure (Louca et al., 2016b) to make predictions about the sediment microbial communities.

## Materials and methods

### Collection of sediment cores and associated chemistry

The Lake Hazen watershed is a polar oasis with temperatures higher than usually found at similar latitudes (Keatley et al., 2007) due to the influence of the Grant Land mountains in the northwest. Sediment cores were collected from three water bodies within the watershed: Lake Hazen itself, Pond1, and Skeleton Lake (Figure S1). Lake Hazen (74 km long, up to 12 km wide, area 54,200 ha, max. depth of 267 m; Figure S2a) is the world's largest lake by volume north of the Arctic Circle. It is primarily fed by runoff from the outlet glaciers of the Grant Land Ice Cap and drained by the Ruggles River to the northeastern coast of Ellesmere Island. Lake Hazen has a relatively stable year-round water temperature of ~3°C (Reist et al., 1995), is fully ice covered in the winter (Latifovic and Pouliot, 2007), and is ultra-oligotrophic (Keatley et al., 2007). Lake Hazen is monomictic, mixing fully in the summer partially influenced by turbidity currents originating from the glacial inflows (Lehnherr et al., 2018). A slight reverse temperature stratification (i.e., lower temperatures right below the ice) develops during the winter. The surface sediments of Lake Hazen are soft silts, with a total organic carbon content between 3.1 and 8.3%. The bathymetry and geochemistry of Lake Hazen have been thoroughly characterized in Köck et al. (2012). While large lakes like Lake Hazen are rare in the Arctic, small lakes and shallow ponds are a characteristic feature of the Arctic landscape. Skeleton Lake (1.9 ha, max. depth 4.7 m; Figure S2b) is fed by permafrost thaw waters, and subsequently drains through two ponds, a wetland, and a small creek before flowing into Lake Hazen (Emmerton et al., 2016). Pond1 (0.1–0.7 ha, max. depth 0.5–1.3 m; Figures S2c,d) is located along the northwestern shore of Lake Hazen. In high glacial runoff years, Pond1 may become hydrologically connected to Lake Hazen as water levels rise (Emmerton et al., 2016; Figure S2d). The organic carbon content of the sediments in Pond1 ranges from 7.0 to 10.4% and in Skeleton Lake from 13.0 to 35.1%. Skeleton Lake and Pond1 are fairly productive in the summer with photosynthesis by macrophytes, mosses, and algal mats that cover the sediments (Figure S2c), despite their low chlorophyll *a* concentration (Keatley et al., 2007; Lehnherr et al., 2012). Some of the productivity in Skeleton Lake and Pond1 might also be driven by carbon and nutrients originating from fecal matter of birds as both sites are important nesting habitats. In the summer, their water temperature can rise to 19°C, but in the winter, ice cover reaches to the bottom in Pond1 and shallower (<2 m) parts of Skeleton Lake. The water columns of both Skeleton Lake and Pond1 are depleted of O_2_ during the winter because of heterotrophic activity.

Short sediment cores were collected over three field expeditions: (i) in spring 2014 from two sites in Lake Hazen itself (Snowgoose Bay [depth: 44 m] and Deep Hole [258 m]; Figures S2a, S3a), (ii) in spring 2015 from three sites in Lake Hazen (off John's Island [141 m], Snowgoose Bay [50 m], and Deep Hole [261 m]) plus one site at the center of Skeleton Lake [4 m] (Figures S1, S3b), and (iii) in summer 2015 from Pond1 [1.5 m] plus a shallow shoreline site [0.3 m] in Skeleton Lake (Figures S1, S3c,d). In spring, all sites were covered with just less than 2 m of snow-covered ice; in summer, samples were collected during open water (ice-free) conditions. At each site, three intact replicate cores were collected for DNA extraction, and determination of physicochemical profiles and of porewater chemistry. All sediments were collected either with an UWITEC (Mondsee, Austria) gravity corer (deep sites), or manually (shallow sites in Pond1 and Skeleton Lake) into 86 mm inner diameter polyvinyl chloride core tubes. Due to logistical constraints, only a single core was available for DNA extraction from each time and site. Cores for DNA extraction were sectioned in 0.25 cm (spring 2014) or 0.50 cm (summer 2015) intervals immediately after sampling, preserved in Invitrogen™ RNAlater™ (Thermo Fisher Scientific Inc., Waltham, MA, USA), and stored at −18°C before DNA extraction. Contamination of samples was minimized by cleaning the sectioning equipment between each section and wearing non-powdered latex gloves during sample handling. In spring 2015, whole cores were frozen directly after sampling at −18°C, transported back to the University of Ottawa, and sectioned at 1 cm intervals while frozen. Surfaces of the sections in contact with the non-sterile sectioning equipment were scraped clean with bleach-sterilized tools in a laminar flow hood (HEPA 100) before subsampling from the middle of the sections. Redox potential, pH, [H_2_S], and dissolved [O_2_] profiles were measured at 100 μm intervals in the field within an hour of collection, using Unisense (Aarhus, Denmark) microsensors connected to the Unisense Field Multimeter (Tables S1, S2). Redox and [H_2_S] data were unavailable for summer 2015 cores because of broken microsensors. For the summer 2015 cores [${\text{NO}}_{3}^{-}$], [Cl^−^], and [${\text{SO}}_{4}^{2--}$] were also measured in sediment porewaters by ion chromatography (Table S2). Cores used for analyses of porewater chemistry were sectioned at 1 cm intervals into 50 ml falcon tubes in the field, followed by flushing of any headspace with UHP N_2_ before capping. Tubes were then centrifuged at 4,000 rpm, after which the supernatant was filtered through 0.45 μm cellulose nitrate filters into 15 ml tubes, which were then frozen until analysis at the Elemental Analysis and Stable Isotope Ratio Mass Spectrometry Laboratory (Department of Renewable Resources, University of Alberta). Concentrations for H_2_S were set to 0 where it was not detected with the microsensors. For the three lowest horizons in the Skeleton Lake 2015 core, [H_2_S] was input as the value measured at the deepest sediment depth before the microsensor broke (169.8 mgL^−1^), as a conservative estimate since its oxidation in the completely anaerobic sediments was likely minimal. When several measurements were made over the sectioning depth used for DNA extraction, concentration readings were averaged. Hereafter, “sediment depth” refers to the lower sediment depth of each sample, measured down from the sediment-water interface. Principal Components Analysis (PCA) was employed to visualize physicochemical differences and relatedness between the different coring sites. For this, the autoplot function from the R package ggfortify 0.4.1 (Horikoshi and Tang, 2017) was used.

### Sequencing and data preprocessing

Upon returning to the University of Ottawa, samples for DNA extraction were homogenized, divided into duplicate 250 mg (WW) subsamples, and washed with a buffer (10 mM EDTA, 50 mM Tris-HCl, 50 mM Na_2_HPO_4_·7H_2_O at pH 8.0) to remove PCR inhibitors (Zhou et al., 1996; Poulain et al., 2015). DNA was extracted from the duplicate subsamples with PowerSoil® DNA Isolation Kit (MO BIO Laboratories Inc, Carlsbad, CA, USA), and then the duplicate extracts were combined. The 16S rRNA gene fragment was amplified with universal primers in the spring 2014/2015 samples, and primer sets specific to either Bacteria or Archaea in the summer 2015 samples (for details, see SI text). The extraction kit elution buffer was used as a negative control in screening experiments. Sequencing was completed with Illumina MiSeq using paired-end 300 bp reads at Molecular Research LP (Shallowater, TX, USA; for details, see SI text). Sequencing of a single sample per sediment depth per core was deemed sufficient, since no pairwise comparisons of individual samples were conducted in the data analysis. All handling of the samples was conducted in a laminar flow hood (HEPA 100) stainless steel sterile cabinet that was treated with UVC radiation and bleach before each use.

Forward and reverse reads were paired with PEAR 0.9.10 (Zhang et al., 2014a), and libraries were split with QIIME 1.9.1 (Kuczynski et al., 2011). Chimeric sequences were removed with vsearch 2.0.0 (Rognes et al., 2016) utilizing the UCHIME (Edgar et al., 2011), against the SILVA 128 SSU Ref NR99 database (Quast et al., 2013). The reads were clustered into OTUs with Swarm 2.1.9 (Mahé et al., 2015) and singleton OTUs were removed. Counts were normalized using cumulative sum scaling with the Bioconductor package metagenomeSeq 1.18.0 (Paulson et al., 2013). Representative sequences of the OTUs were aligned to the SILVA 128 database (Quast et al., 2013), with SINA Incremental Aligner 1.3.0 (Pruesse et al., 2012). Taxonomy was assigned to the OTUs in SINA, with the Least Common Ancestor method. For phylogeny-based analyses, the alignments were trimmed with trimAl 1.2 using the heuristic “automated1” option (Capella-Gutiérrez et al., 2009) followed by visual inspection in Unipro UGENE 1.26.3 (Okonechnikov et al., 2012). Maximum likelihood phylogenetic trees were built with FastTree 2.1.9 (Price et al., 2010), using the GTR + Γ model of sequence evolution (Aris-Brosou and Rodrigue, 2012).

### Data analyses

The number of sequences was tracked throughout each step of the pipeline for quality control (Table S3). The taxonomy of OTUs with >99% sequence identity to the SILVA 128 database was refined to the closest matching entry to facilitate functional mapping. OTUs with ambiguous, mitochondrial, or plastid assignments were removed with phyloseq 1.20.0 (McMurdie and Holmes, 2013). Negative controls were not sequenced for this study and, as such, we were not able to directly remove possible contamination brought by the DNA extraction kit. Although studies with low microbial biomass (e.g., blood, lungs, dry surfaces) are expected to be more sensitive to contaminants (Salter et al., 2014; Glassing et al., 2016), we tested the impact of possible contaminants by identifying and removing putative contaminating genera from our samples (see SI text; Figure S4). We compared the unmodified data to analyses where we removed 100% of known contaminants from MOBIO PowerSoil DNA extraction kits (Glassing et al., 2016), the kit used in our study. The result of our comparative analyses showed (i) no changes in alpha diversity analyses, (ii) few changes in the clustering analyses (Figure S5) and (iii) no changes in the ordination analyses (Figure S6), leaving our conclusions unaffected in all cases. Note that e.g., 5 of the 10 most abundant contaminants (Veillonella, Methylobacterium, Prevotella, Tumebacillus, and Oxalobacter) were not found in our samples. In addition to known contaminants from the MOBIO kit used here and described in the main text, we also tested for known contaminants from four additional DNA extraction kits (Salter et al., 2014) that were not used in our study (see SI text; Figures S4–S6). However, as the putative contaminant genera could plausibly be part of the sediment community and the identity of true contaminants are not known, they were not removed from the data. To visually estimate the sequencing depth in our samples, rarefaction curves were constructed from non-normalized data with singleton OTUs included (Figure S7). To assess the functional potential of the communities based only on 16S rRNA gene amplicon data, the normalized and curated OTU abundances were mapped to phylogenetically conserved functional groups in a customized database using FAPROTAX 1.0 (see SI text; Figures S8, S9; Louca et al., 2016b). Briefly, the predictions made by FAPROTAX are based on references from the literature, and work by mapping OTUs (at any given taxonomic level) to functional groups. The associations are based solely on cultured strains, so that an association between a taxonomic level and a functional group is only made if all representatives at that taxonomic level display the particular function. The total DNA extracted from sediments does not solely represent the metabolically active part of the community, as DNA from both dormant and dead organisms is usually co-extracted (Klein, 2007; Carini et al., 2017; Lennon et al., in review). Thus, without transcriptomic or proteomic data our functional predictions should be considered hypothetical.

To assess the biological significance of phylogenetic characterizations, samples were analyzed based on two levels of diversity: within and among samples. First, we investigated trends in alpha-diversity (within-sample diversity). Because the contribution of individual taxa to ecosystem processes is likely dependent on their abundance (Nemergut et al., 2013), we chose Simpson's dominance (Morris et al., 2014) as the metric for alpha-diversity. Simpson's dominance is robust to both spurious OTUs and variations in sampling depth between sequencing runs (Pinto and Raskin, 2012). The sequencing depth in our samples (Good's coverage: 76.0–97.2%; Figure S10) suffices to accurately estimate alpha-diversity (Lundin et al., 2012). To enable comparisons of alpha-diversity and sequencing coverage to other studies, we also calculated Chao1 and Shannon indices, and Good's coverage (Figure S10). All this was done based on ten randomized rarefactions of the raw OTU counts with the R package phyloseq 1.20.0 (McMurdie and Holmes, 2013). The relationships between alpha-diversity and its predictors (sample categories or physicochemical variables) were determined with random forests (Breiman, 2001; Liaw and Wiener, 2002). The forests were grown to 5,000 trees, using the R package ranger 0.7.0 (Wright and Ziegler, 2015). Selection of the most important predictors was based on the Gini index by adding predictors one by one in order of decreasing importance (Menze et al., 2009). The best and most parsimonious model was then selected by minimizing the Model Standard Prediction Error (MSPE) for regression random forests (in the case of continuous predictors), or by maximizing Cohen's Kappa for classification random forests (in the case of categorical predictors). The relationships between the most important predictors and Simpson's dominance were estimated with partial dependence plots of the best models with the R package edarf 1.1.1 (Jones and Linder, 2016), which display how model prediction changes as a function of each predictor, while other predictors are fixed to their average value. Thus, each variable's effect on the model prediction is considered independently, and each predictor's relative effect size can be estimated from the variability displayed by the model prediction.

Second, in terms of beta-diversity (between-samples diversity), phylogenetic distances between pairs of samples were calculated with a Double Principle Coordinate Analysis (DPCoA; Pavoine et al., 2004), using OTU abundances and patristic distances estimated from the maximum likelihood tree. The phylogenetic data were limited to OTUs with >0.01% overall abundance, because of the quadratic increase in runtime per added OTU in DPCoA (Fukuyama et al., 2012). The Bray-Curtis distances between samples were calculated from group abundances in the functional predictions. A Mantel test was used to test for differences between sample physicochemistry, and either their phylogenetic or functionally predicted group distances. Phylogenetic data and functional predictions from spring 2014/2015 were then clustered using the *t*-distributed stochastic neighbor embedding (tSNE) algorithm (van der Maaten and Hinton, 2008), implemented in the R package rtsne 0.13 (Krijthe and van der Maaten, 2017), with “perplexity” set to 5. Clusters were identified with the HDBSCAN algorithm (Campello et al., 2013), in the package dbscan 1.1.1 (Hahsler et al., 2017), with “minPts” set to 3. Regression and classification random forests were used together with partial dependence plots to identify the most important physicochemical and categorical variables for the clustering patterns, as described above. Two different subsets of the phylogenetic data were analyzed: the most abundant OTUs (>0.01% abundance) and the dataset limited to OTUs that matched at least one group in the FAPROTAX database. Summer 2015 data were not included in the tSNE analyses because only a single core per lake was available.

The correlations between categorical and continuous variables to beta-diversity were assessed by unconstrained correspondence analysis with “envfit” from the R package vegan 2.4.3 (Oksanen et al., 2016). The variables were fit on NMDS ordinated distance matrices (described above) for both phylogenetic data and functional predictions, and the statistical significance was assessed with 10,000 permutations. For the continuous physicochemical variables, non-linear relationships were analyzed with “ordisurf,” which is based on surface fits, contra vector fits in envfit (**Figure 6**, Figures S12–S14). All *P*-values were Bonferroni-corrected per data set. Random forests (described above) were further used to corroborate these analyses. In the phylogenetic data, OTU abundances were grouped at phylum, class, and order levels for the random forest models, which were all screened for the best and most parsimonious model. Lower (i.e., more exclusive) taxonomic levels were disregarded to increase the ecological meaningfulness of the results (Xu et al., 2014). Partial dependence plots were again generated with the R package edarf to examine the relationships between the most important OTUs and their functionally predicted group abundances to each sample category, spatial, and environmental variable. All these data analyses were done with R 3.4.0 (R Core Team, 2017); the corresponding scripts can be accessed through GitHub (https://github.com/Begia/Hazen16S), the sequencing data can be retrieved from the NCBI Sequence Read Archive (https://www.ncbi.nlm.nih.gov/bioproject/PRJNA430127), and the geochemical data from the National Centers for Environmental Information online repository (http://accession.nodc.noaa.gov/0171496).

## Results and discussion

### Sediment microbial communities are similar to other arctic lakes

**Figure 1:**
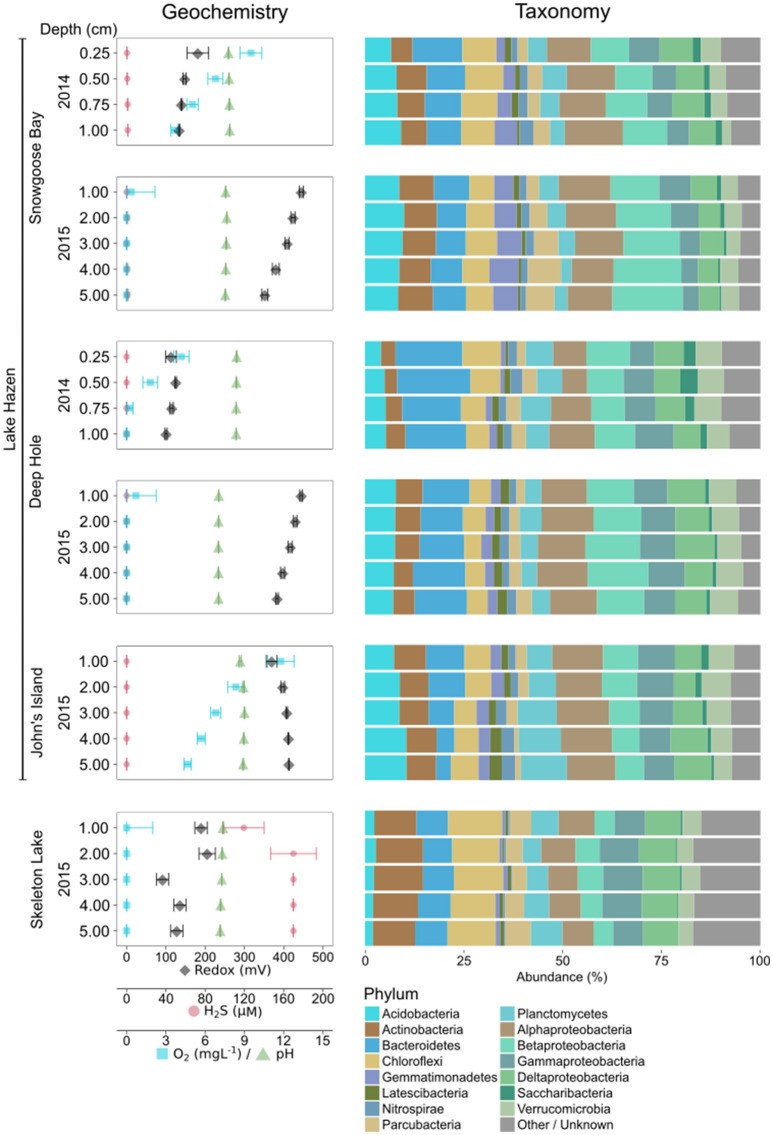
Geochemical variability, and microbial community composition of the spring 2014/2015 samples using universal primers. Abundances of taxa have been merged at the phylum level (Proteobacteria at class level). Phyla with less than 1% overall abundance in the data set are merged.

Microbial community structure of Lake Hazen and Skeleton Lake sediments in spring 2014/2015 exhibited similarities to other lake sediments in polar (Tang et al., 2013; Wang et al., 2016; Mohit et al., 2017) and high-altitude regions (Zhang et al., 2014b) that have comparable ranges of temperature, nutrient and light availability. The most abundant bacterial phyla at our sampling sites were Proteobacteria, Bacteroidetes, Chloroflexi, Actinobacteria, Acidobacteria, Planctomycetes, and Verrucomicrobia (see SI text; Figure 1). The dominant archaeal phylum at all sites was Woesearchaeota, similarly to water columns of oligotrophic high-altitude lakes (Ortiz-Alvarez and Casamayor, 2016).

Sediments in Skeleton Lake had higher abundances of Chloroflexi, Actinobacteria, Cyanobacteria, and archaeal phyla, while Acidobacteria were more abundant in Lake Hazen sediments. Differences between the lakes might be driven by better light availability at the sediment-water interface, and higher production of sulfide in Skeleton Lake sediments compared to Lake Hazen. Indeed, all coring sites from Lake Hazen had overlying water columns of more than 40 m, measurable dissolved [O_2_] in the top 1 cm (John's Island samples had >4.7 mgL^−1^ O_2_ down to 5 cm), and low [H_2_S] (< 1.2 μM). Furthermore, although toxic, low levels of H_2_S can enhance cyanobacterial photosynthesis when light intensity is low (Klatt et al., 2015). Hence, Cyanobacteria in Skeleton Lake might be able to photosynthesize below the ice cover in the spring.

**Figure 2:**
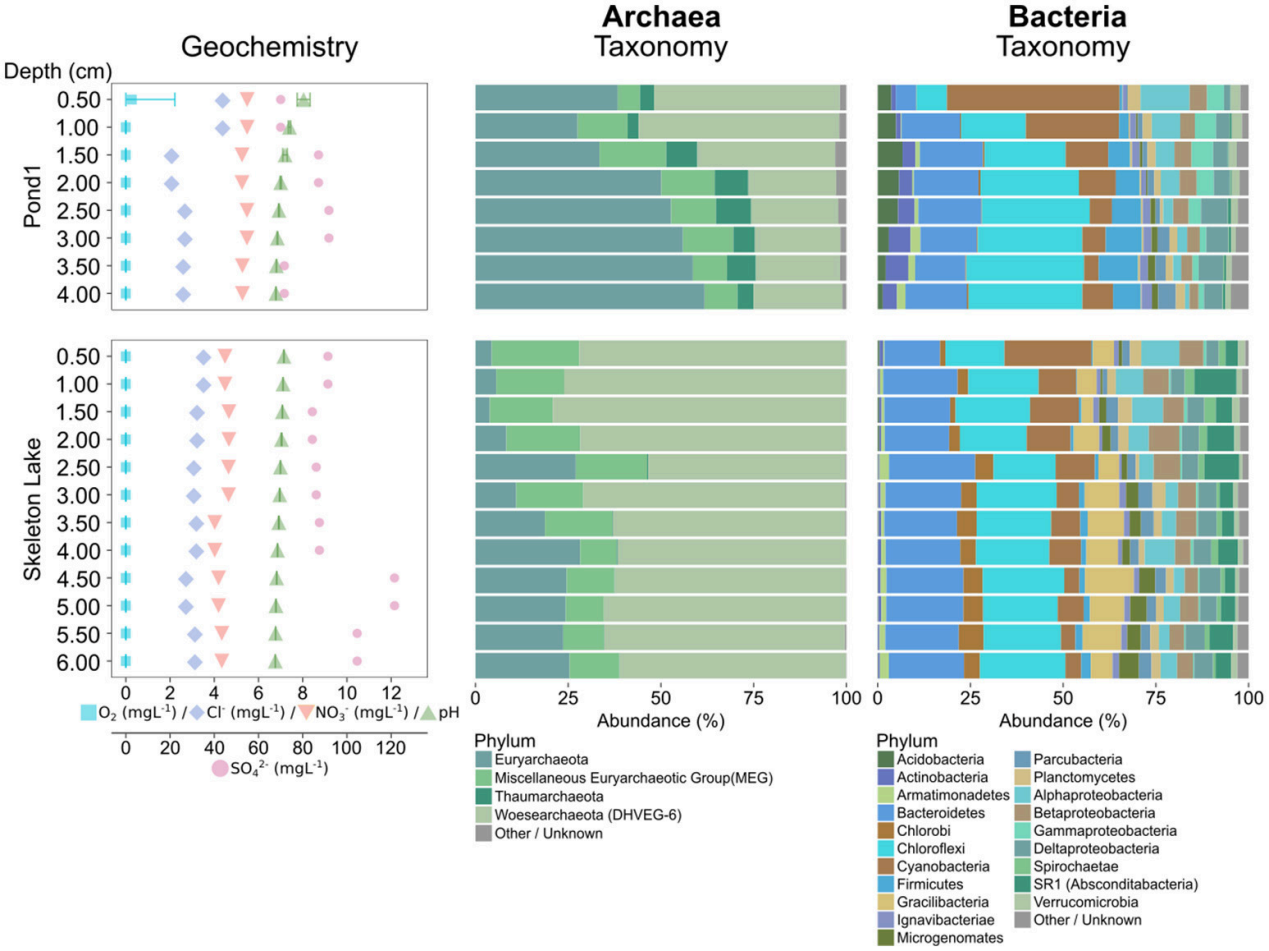
Geochemical variability, and microbial community composition of the summer 2015 samples using archaeal and bacterial primers. Abundances of taxa have been merged at the phylum level (Proteobacteria at class level). Phyla with less than 1% overall abundance in each data set are merged.

In sediments sampled in summer 2015, Chloroflexi was the most abundant bacterial phylum in both Skeleton Lake and Pond1 (Figure 2). Their high abundance has been previously observed in hypersaline methane-rich springs in the High Arctic (Lamarche-Gagnon et al., 2015). In the current study, the salinity was low ([Cl^−^] < 4.4 mgL^−1^), but Skeleton Lake, and the ponds bordering Lake Hazen are methanogenic (Emmerton et al., 2016). The archaeal communities in Skeleton Lake sediments were dissimilar to those in Pond1. Woesearchaeota were more common in Skeleton Lake, and Euryarchaeota in Pond1 sediments.

Mercury methylation had previously been quantified in both Skeleton Lake and Pond1 (Lehnherr et al., 2012), but its microbial actors were unknown. Fourteen OTUs in our data mapped to mercury methylation in our custom functional mapping database, and their 16S sequences, all matched closely to *Methanosphaerula palustris* (Cadillo-Quiroz et al., 2009). The genome of the type strain of this species has been shown to possess the *hgcAB* genes that strongly predict mercury methylation capability (Gilmour et al., 2013). Other taxa most likely also take part in mercury methylation in these sediments. However, our amplicon-based study might have missed their presence because of primer bias and low 16S database coverage of organisms in these environments.

Intra-lake/pond compositional variability could also be high. For instance, the communities at the two sites sampled in Skeleton Lake in spring (Figure 1) and summer 2015 (Figure 2), were strikingly different. Sediments from the deeper site (Figure S3b), sampled in spring 2015 under ice cover, had a mostly heterotrophic community dominated by Proteobacteria. Meanwhile, sediments from the shallower site (Figure S3d), sampled in summer 2015, were dominated by phototrophs such as Chloroflexi and Cyanobacteria, and anaerobic fermenters such as Bacteroidetes and Gracilibacteria (Thomas et al., 2011; Wrighton et al., 2012). This indicates high spatial heterogeneity of sediment communities in Skeleton Lake sediments. Primer bias and seasonality of the microbial communities in Skeleton Lake might play a part in this, but the question would require further study. Our qualitative observations of the microbial communities are consistent with (i) measurements of high CH_4_ emissions from ponds bordering Lake Hazen (Emmerton et al., 2016), (ii) increased [MeHg] in Skeleton Lake (unpublished data), (iii) high autochthonous carbon, and (iv) nitrogen limitation at the sites (St. Louis, unpublished data).

### Both redox chemistry and pH drive community diversity and structure

#### Physicochemical data partially explains phylogenetic variability

**Figure 3:**
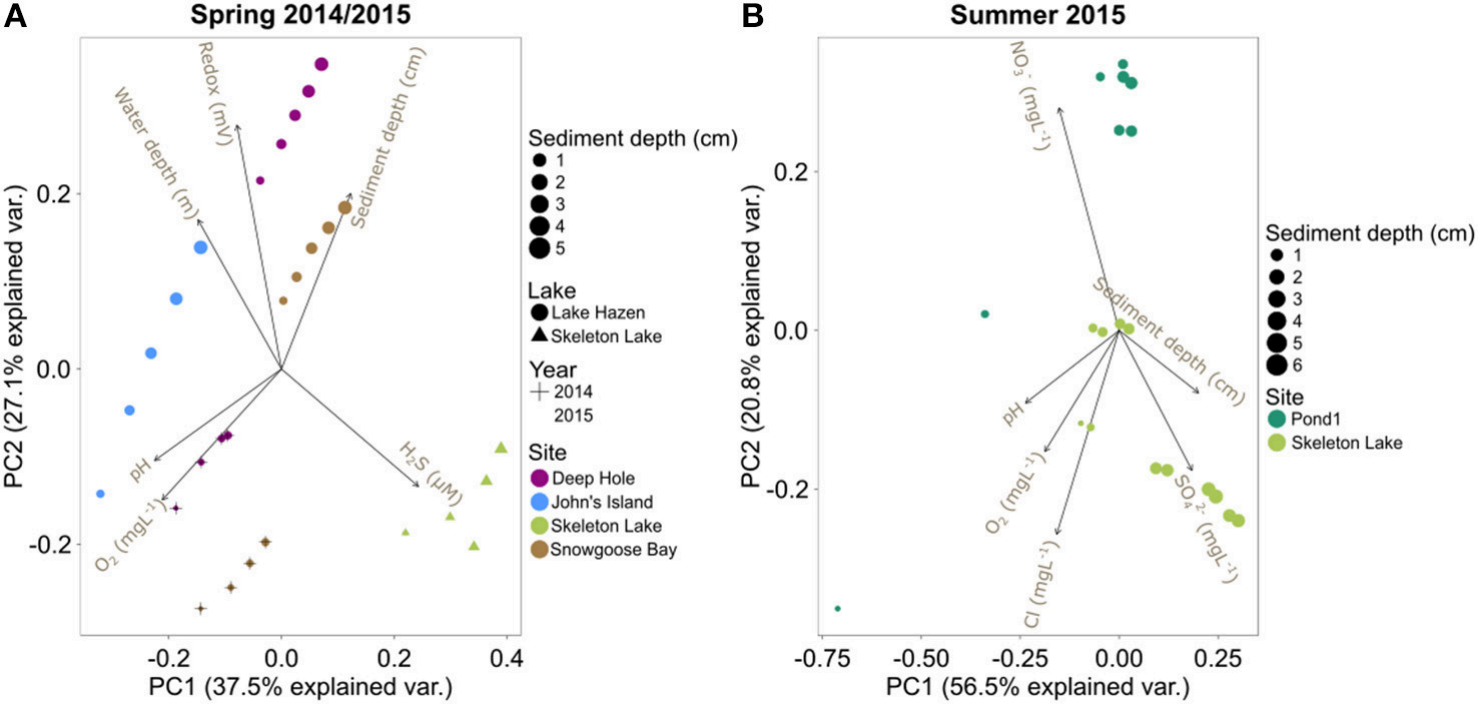
PCA biplots of the physicochemical variables in: **(A)** spring 2014/2015 samples, and **(B)** summer 2015 samples.

The phylogenetic variability may be driven by the unique physicochemical properties of each site, which can vary substantially both in time and in space. A PCA of the physical and geochemical variables in spring 2014/2015 shows that samples group by individual core (Figure 3A). More specifically, the PCA revealed two major independent (orthogonal) axes of variability: (i) [H_2_S]/redox/water depth, and (ii) pH/[O_2_] (Figure 3A). Samples with measurable [H_2_S] had lower redox potential and were from shallower sites (mostly from Skeleton Lake; Table S1). Samples closer to the sediment/water interface had higher pH and [O_2_]. Our sampling likely captured some of the most relevant physicochemical variables constraining the microbial community structures. Indeed, the Euclidean distances between the samples calculated from physicochemical variables were correlated with their phylogenetic DPCoA distances (Mantel-R^2^ = 0.57, Bonferroni-corrected *P* < 0.01). The physicochemical variability also correlated with the functionally predicted group abundances (Bray-Curtis distances; Mantel-R^2^ = 0.40, Bonferroni-corrected *P* < 0.01). However, only 25% of the OTUs could be mapped to any function and were thus covered by this analysis.

The PCA on the summer 2015 physicochemical data revealed that these sediments also clustered separately, with Pond1 on one side of PC2 and Skeleton Lake on the other side (Figure 3B). Most of the differences between these two sites were driven by higher [${\text{NO}}_{3}^{-}$] in sediments from Pond1 and higher [${\text{SO}}_{4}^{2-}$] in sediments from Skeleton Lake, while pH, [O_2_], and [Cl^−^] covaried. However, the top 1 cm surface sediments from Pond1 were highly influential in the PCA because of their higher pH and [O_2_] than in other samples (Figure 3B, Table S2). This higher pH and [O_2_] could reflect the influence of the incoming Lake Hazen waters into Pond1, which tend to be higher in pH and O_2_, especially in the summer under the direct influence of the glacial inflows. It is possible that this difference in the scaling of the sites along the PCA reflects different water sources between Pond1 and Skeleton Lake.

Unlike with the spring 2014/2015 data, the summer 2015 data showed no significant correlations between the physicochemical distances of the samples, and either phylogenetic data or functional predictions (bacterial and archaeal; all Bonferroni-corrected *P* > 0.05). This indicates that the measured physicochemical variability does not explain differences in community structures among samples. Unknown variables, such as redox potential, might be influencing the community assembly at these sites. Furthermore, the two sites in this data set have similar physicochemistry throughout each sediment profile, which probably reduces discriminatory power for this analysis.

#### Higher redox potential and lower sulfide concentration drive alpha-diversity

**Figure 4:**
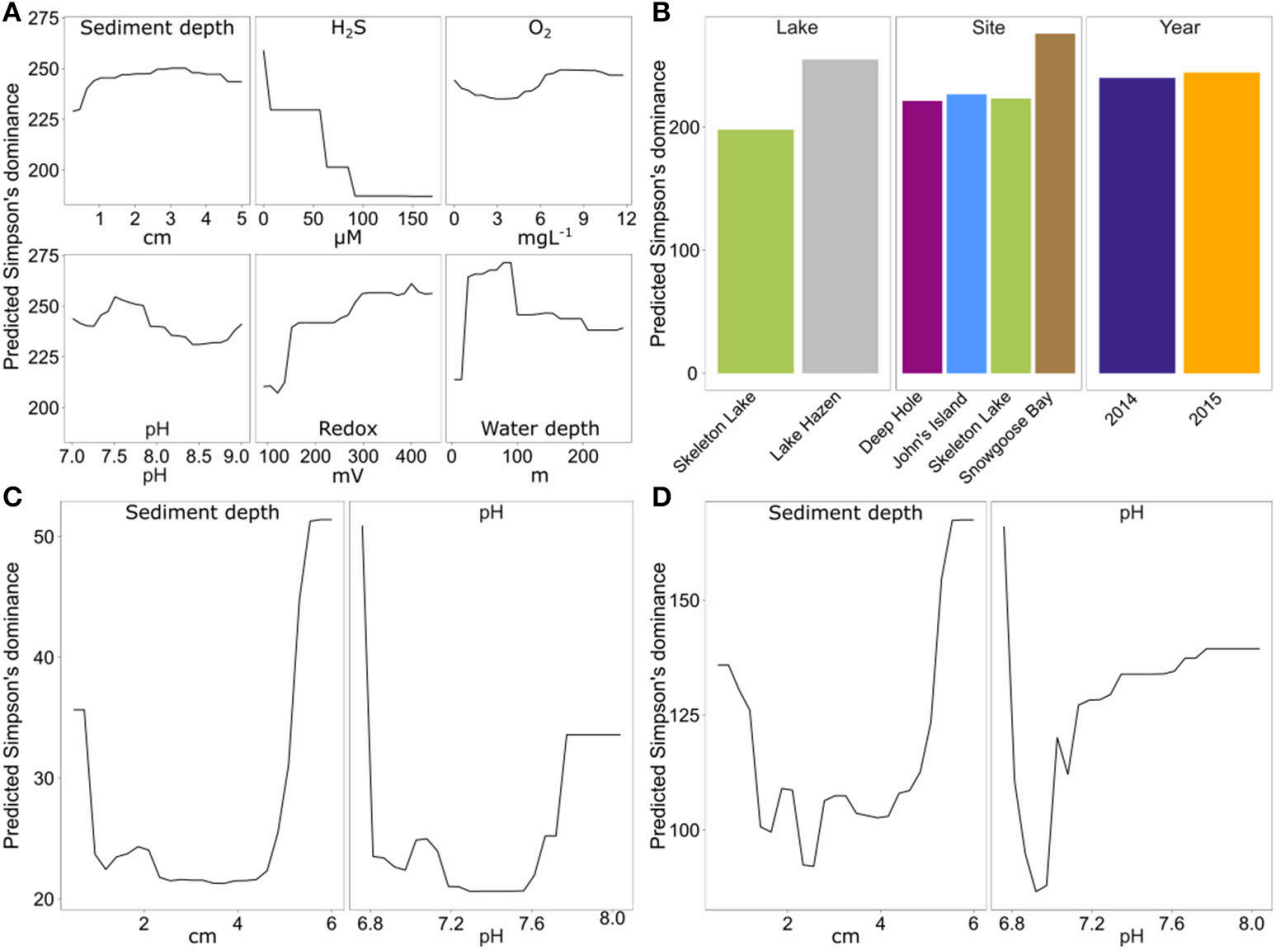
Partial dependence of predicted Simpson's dominance on continuous and categorical variables of the random forest model with the smallest prediction error, for each of the data sets. Spring 2014/2015 with universal primers **(A)** 6 continuous variables; **(B)** three categorical variables. **(C)** Summer 2015 with archaeal primers (two continuous variables). **(D)** Summer 2015 with bacterial primers (two continuous variables).

To identify the drivers of alpha-diversity at Lake Hazen, we fitted random forest models to our data (Touw et al., 2013). Based on Simpson's dominance, diversity in the spring 2014/2015 sediment samples was best predicted by a model including all physicochemical variables (in order of importance: [H_2_S], overlying water depth, redox potential, site, lake, pH, sediment depth, [O_2_], and year; pseudo-R^2^ = 0.72). These results are consistent with our expectations, as H_2_S can be highly toxic to microbial communities (Hoppe et al., 1990; Brouwer and Murphy, 1995). Water depth was the second most important variable explaining alpha-diversity (Figure 4A). The shallow Lake Hazen sediments at Snowgoose Bay had the highest diversity (Figure 4B, Figure S1; Table S1), which might be driven by high heterogeneity of the sediments, including steep [H_2_S] and [O_2_] gradients. The Snowgoose Bay site is also under the direct influence of two glacial river outlets, which might contribute to the heterogeneity through increased delivery of nutrients and inorganic matter. Our observations are consistent with previous findings of the positive relationship between sediment heterogeneity and alpha-diversity (Lozupone and Knight, 2007). Redox potential, the third most important continuous variable, also had positive relationship with predicted diversity. The effect was similar in magnitude to [H_2_S] and was expected since sulfate reducers are active at low redox potentials. This is consistent with previous studies showing that microbial communities can react quickly to changes in redox potential, changing from aerobic chemoheterotrophy to anaerobic respiration and fermentation (DeAngelis et al., 2010). Finally, we identified pH as a driver of alpha-diversity: since non-extremophilic bacteria need to maintain an optimal intracellular pH of around 7.5 (Booth, 1985), the subsistence of a more diverse community at this pH might be facilitated.

Again, the summer 2015 data set differed from spring 2014/2015 data set, as the best model only included sediment depth and pH as the most important variables for both archaeal (pseudo-R^2^ = 0.53) and bacterial data (pseudo-R^2^ = 0.32). However, direct comparisons between the spring 2014/2015 data and summer 2015 data sets are difficult, since different sites were sampled, and different geochemical variables were measured. Archaeal and bacterial alpha-diversity in the summer 2015 data set were highest in the deepest sediments, with a discrete increase at the sediment surface (Figures 4C,D). The increase in diversity might be caused by higher diversity of organisms with obligate aerobic (at the surface sediments) or anaerobic metabolisms (at deeper sediments). Unfortunately, no reliable data could be obtained for [H_2_S] or redox potential in these samples because of broken microsensors. Here, pH also seemed to be a factor explaining diversity both for archaea and bacteria, but diversity predictions might be driven only by a few outliers at the extremes of sediment depth.

#### Communities cluster phylogenetically by pH and display similar functional predictions

**Figure 5:**
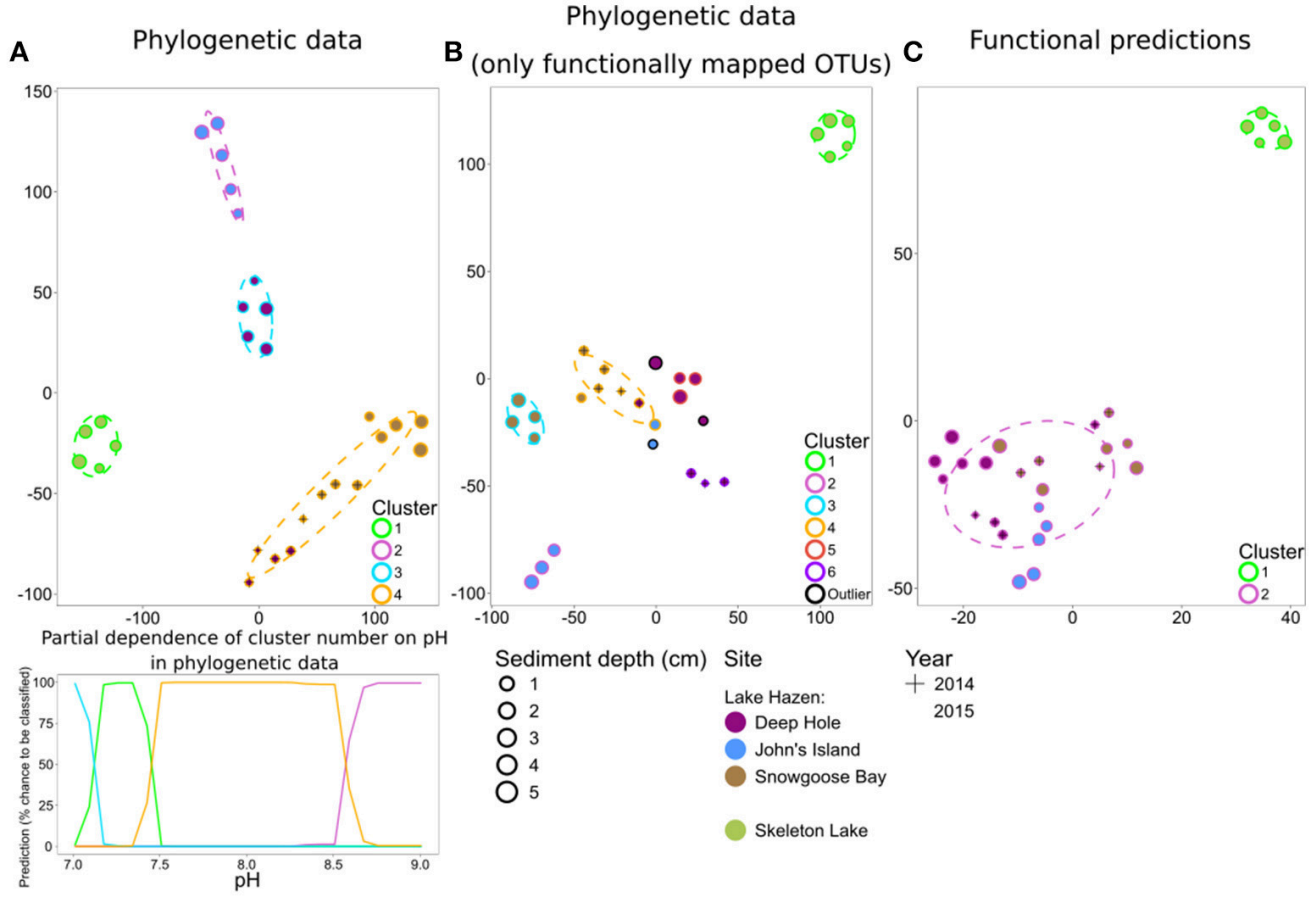
tSNE analysis of spring 2014/2015 samples. Phylogenetic dissimilarities were measured with DPCoA, and differences in functional predictions with Bray-Curtis dissimilarity. **(A)** Phylogenetic data, including only OTUs with >0.01% overall abundance. Partial dependence of cluster number on pH is included for this data below the t-SNE plot. **(B)** Phylogenetic data, including only OTUs that were matched to a function (roughly 25% of the full phylogenetic data). **(C)** Functional predictions.

To independently support these predictions based on random forests, we performed a tSNE cluster analysis on the spring 2014/2015 samples. These samples clustered mostly by individual sediment core for both full phylogenetic data (Figure 5A) and for data including only the 25% of OTUs that could be functionally predicted (Figure 5B). The tSNE analysis of functionally predicted data identified only two clusters, one per lake (Figure 5C). This shows that phylogenetically distinct sediment communities in Lake Hazen have similar functional predictions. Furthermore, Lake Hazen sediments clustered invariably separate from Skeleton Lake sediments in all of these analyses. Random forest classification of the clustering patterns identified pH as the most important predictor for clustering both in the full phylogenetic data set (one predictor; OOB Error = 0%; Figure S11a), and for the functionally mapped OTUs (seven predictors; OOB Error = 12%; Figure S11b). In the full phylogenetic data set, the sediment communities also appear to be more similar to each other over ranges of pH (Figure 4A, lower panel). [H_2_S] was the most important predictor to explain differences between the clusters in the functionally predicted data (one predictor; OOB Error = 0%; Figure S11c). However, because we sampled only a single site in Skeleton Lake in spring 2015, it remains uncertain if [H_2_S] is the only factor affecting the observed difference in the functionally predicted groups in the sediments of the two lakes. Furthermore, heterogeneity of the communities within Skeleton Lake itself could not be addressed with the clustering analysis due to only a single core being analyzed. Regardless, all the phylogenetically distinct communities in Lake Hazen sediments clustered together after functional prediction.

Our results suggest that pH strongly affects phylogenetic community composition in our samples. Indeed, pH has previously been shown to be a major determinant of community composition in similar lake sediments (e.g., Xiong et al., 2012). Sediment microbial communities might be altered in the future because climate change related effects can increase pH in arctic lakes (Kokelj et al., 2005; Mesquita et al., 2010). We observed that the microbial communities in Lake Hazen sediments cored at different sites at different times are phylogenetically distinct from each other and Skeleton Lake sediments. All the samples from Lake Hazen displayed similar functional predictions, while remaining distinct from Skeleton Lake samples. Decoupling between phylogeny and function of microbial communities has previously been observed, e.g., in the global ocean microbial communities (Louca et al., 2016b), and plant-associated environments (Louca et al., 2016a). However, our results rely solely on the analysis of 16S rRNA genes, and therefore lack direct evidence about the actual microbial functioning and activity in the lake sediments. Critical insights could be gained here by employing metagenomics, metatranscriptomics, and (ideally) metaproteomics (Louca et al., 2016a).

#### Beta-diversity is also driven by redox chemistry and pH

**Figure 6:**
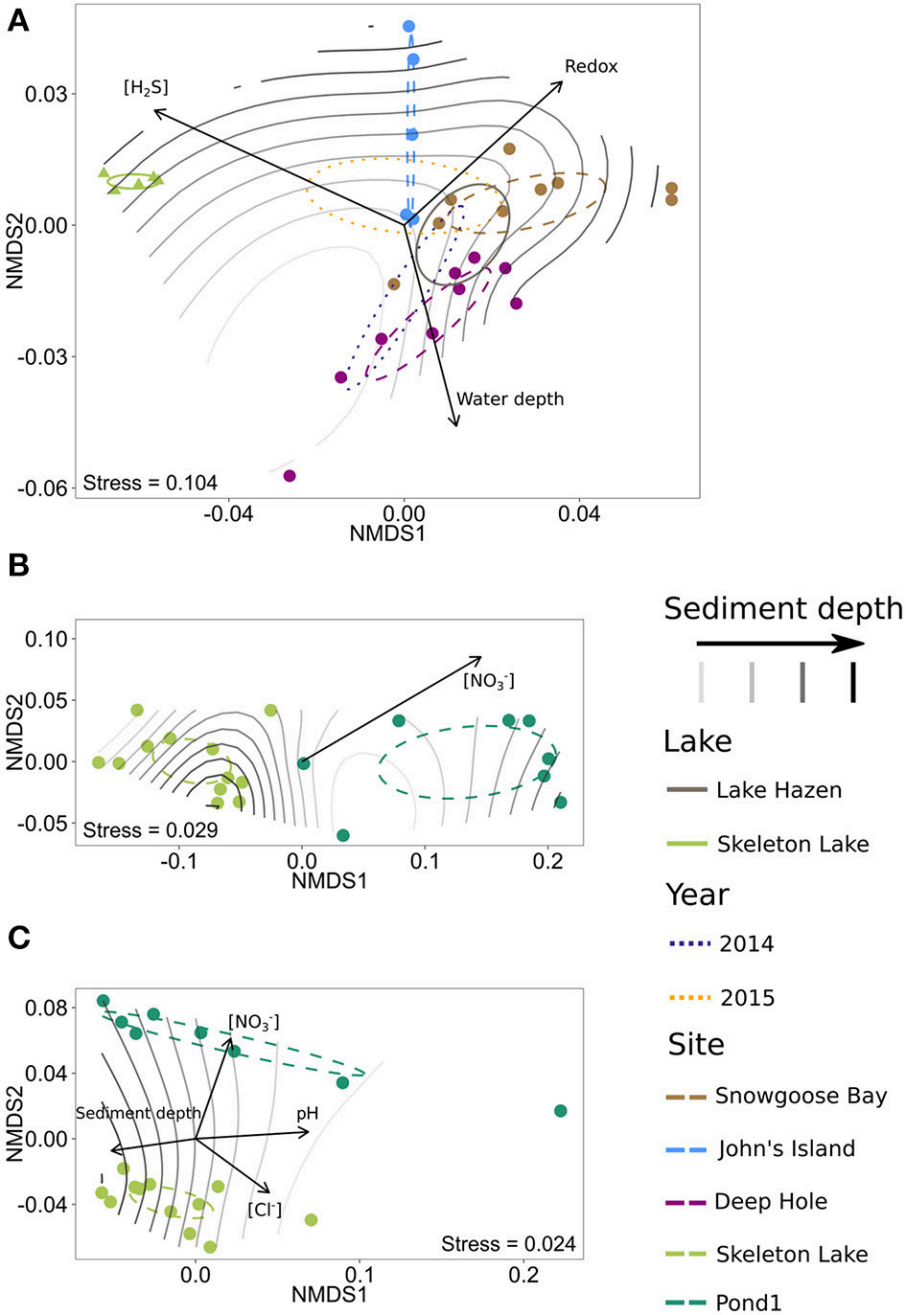
NMDS ordinations of phylogenetic DPCoA distances of the samples, with significantly correlated physicochemical variables as vectors. 95%-confidence interval for centroids of sample categories (lake, year, and site, where applicable) is shown with ellipses and sediment depth is overlaid on the plots as a surface fit. **(A)** Spring 2014/2015 with universal primers. **(B)** Summer 2015 with archaeal primers. **(C)** Summer 2015 with bacterial primers.

For the spring 2014/2015 bacterial communities, the centroids of the clusters found by NMDS ordinations for both lakes and individual sites were different from each other (Bonferroni-corrected *P* < 0.01), but no year effect could be found (Bonferroni-corrected *P* > 0.05; Figure 6A). As both spring 2014 and spring 2015 samples were also sequenced using the same primer set, we analyzed samples from different years together. The communities in Skeleton Lake sediments were phylogenetically distinct from Lake Hazen sediments, and the communities in individual Lake Hazen cores were also phylogenetically dissimilar to each other. While these patterns are consistent with the tSNE analysis, they are not as clear because NMDS preserves pairwise distances instead of emphasizing them (like tSNE). [H_2_S], redox potential, and water depth correlated linearly with phylogenetic distances of the communities (Bonferroni-corrected *P* < 0.05; Figure 6A). Sediment depth was not linearly correlated with the phylogenetic distances, but the communities at the sediment surface might be more similar to each other than communities deeper in the sediment. This can be observed in the grouping of surface samples together in the middle of the ordination (Figure 6A). The deepest sediments at John's Island appeared quite unique, which might be due to the presence of O_2_ all the way down to 5 cm below sediment surface, whereas O_2_ is not found at any other sites below 1 cm (Figure 1, Table S1).

Archaeal communities in sediments from Pond1 and Skeleton Lake (summer 2015) also differed from each other phylogenetically (Bonferroni-corrected *P* < 0.01; Figure 6B). However, [${\text{NO}}_{3}^{-}$] was the only physicochemical variable linearly correlated with phylogenetic differences of the communities in the samples (Bonferroni-corrected *P* < 0.05). Similar to archaeal communities, bacterial communities in sediments from Pond1 and Skeleton Lake (summer 2015) were phylogenetically significantly different from each other (Bonferroni-corrected *P* < 0.01; Figure 6C). [${\text{NO}}_{3}^{-}$], pH, sediment depth and [Cl^−^] correlated linearly with phylogenetic differences between the samples (Bonferroni-corrected *P* < 0.05). The communities in surface sediments of both Pond1 and Skeleton Lake seemed most dissimilar to the other samples from the same core.

Altogether, beta-diversity seems to be affected mostly by [H_2_S], redox potential and pH. These are the variables that have surfaced in either the ordination or tSNE analysis for both spring 2014/2015 and summer 2015 data sets. In addition, we also observed trends with water depth in spring 2014/2015 data and [${\text{NO}}_{3}^{-}$] in the summer 2015 data set. The effects of [H_2_S] and redox potential are probably linked to toxicity of H_2_S and different availability of electron acceptors in the changing redox potential, which together alter the community composition. Water depth in the spring 2014/2015 data set can be seen as a proxy for several factors influencing community structure; the depth of the overlying water column influences both light availability and sediment dynamics, such as differences in sedimentation rate and nutrient inputs, resuspension, and sediment focusing. However, the trends in summer 2015 data with [${\text{NO}}_{3}^{-}$] are questionable, as (i) [${\text{NO}}_{3}^{-}$] covaries with sulfate and sediment depth (deeper sediment horizons have lower nitrate and higher sulfate; Figure 3B; Table S2), and (ii) [${\text{NO}}_{3}^{-}$] in Pond1 is much higher than in Skeleton Lake (Table S2).

### Taxonomic group abundances vary along physicochemical gradients

We conducted random forest analyses to discover relationships between physicochemical gradients and abundances of taxonomic groups, and our functional predictions (see SI text; Tables 1, 2; Figures S15–S37). We found an association between increasing levels of [H_2_S] and (i) decreasing abundances of aerobic taxa and functionally predicted aerobic groups (putative aerobic ammonia oxidizers, aerobic chemoheterotrophs, aerobic nitrite oxidizers, and predatory/exoparasitic microbes), and (ii) increasing abundances of functionally predicted sulfate respirers, methanogens, and cyanobacteria (cyanobacteria are all photosynthetic and thus mapped to a single group; Figure S15). Skeleton Lake sediments had much higher [H_2_S] than Lake Hazen sediments, but the community differences linked to [H_2_S] are not completely explained by differences between the lakes (Figure S34). [H_2_S] seems to affect both phylogenetic and functionally predicted community composition, and climate change has previously been thought to result in increased accumulation of sulfur in high arctic lake sediments (Drevnick et al., 2010). Chemical weathering of sulfate containing minerals (e.g., gypsum-CaSO_4_) following glacial melt and/or permafrost thaw could also increase delivery of SO_4_ to waterbodies in the Lake Hazen watershed. Enhanced rates of sulfur cycling in sediments might change the community structure, which might affect other geochemical cycles mediated by the sediment communities.

**Table 1:** Summary of the regression random forest models for continuous variables.

			**Before model selection**	**After model selection**		
**Data set**	**Variable**	**Taxonomic level**	***n* (predictors)**	***n* (predictors)**	**MSPE (95% CI)**	**pseudo-R^2^**
Spring 2014/2015	H_2_S	Class	164	16	132.67 (0.00–267.65)	0.966
		Functional mapping	48	7	97.48 (15.62–179.35)	0.961
	pH	Order	280	8	0.06 (0.03–0.08)	0.875
		Functional mapping	48	3	0.07 (0.03–0.10)	0.851
	O_2_	Order	280	3	5.42 (1.37–9.46)	0.560
		Functional mapping	48	7	5.99 (2.45–9.53)	0.514
	Redox potential	Order	280	8	3978.13 (1794.36–6161.90)	0.793
		Functional mapping	48	2	5036.48 (2100.30–7972.66)	0.738
	Sediment depth	Class	164	2	1.10 (0.58–1.63)	0.591
		Functional mapping	48	9	1.62 (0.87–2.38)	0.398
	Water depth	Class	164	2	738.28 (0.00–1484.27)	0.932
		Functional mapping	48	3	3121.19 (1611.48–4630.90)	0.711
Summer 2015/Archaea	pH	Class	13	3	0.04 (0.00–0.11)	0.538
		Functional mapping	10	7	0.04 (0.00–0.12)	0.482
	O_2_	Phylum	8	1	0.00 (0.00–0.01)	0.000
		Functional mapping	10	2	0.00 (0.00–0.018)	−0.145
	${\text{SO}}_{4}^{2-}$	Phylum	8	2	77.56 (19.82–135.3)	0.633
		Functional mapping	10	2	105.62 (41.37–169.86)	0.500
	Sediment depth	Order	12	9	0.69 (0.38–1.00)	0.744
		Functional mapping	10	2	0.45 (0.17–0.72)	0.833
	Cl^−^	Order	12	3	0.01 (0.04–0.16)	0.729
		Functional mapping	10	7	0.27 (0.00–0.54)	0.270
	${\text{NO}}_{3}^{-}$	Order	12	9	0.04 (0.01–0.06)	0.869
		Functional mapping	10	2	0.04 (0.01–0.06)	0.867
Summer 2015/Bacteria	pH	Class	85	8	0.04 (0.00–0.09)	0.585
		Functional mapping	26	4	0.04 (0.00–0.10)	0.518
	O_2_	Phylum	37	1	0.00 (0.00–0.01)	0.000
		Functional mapping	26	4	0.00 (0.00–0.01)	−0.052
	${\text{SO}}_{4}^{2-}$	Class	85	2	79.64 (43.64–115.64)	0.623
		Functional mapping	26	3	154.05 (74.19–233.9)	0.271
	Sediment depth	Class	85	1	0.38 (0.20–0.56)	0.858
		Functional mapping	26	5	0.99 (0.59–1.39)	0.631
	Cl^−^	Order	113	10	0.11 (0.03–0.19)	0.695
		Functional mapping	26	10	0.19 (0.03–0.35)	0.465
	${\text{NO}}_{3}^{-}$	Order	113	5	0.03 (0.01–0.04)	0.898
		Functional mapping	26	2	0.03 (0.01–0.04)	0.901

*Number of predictors (n) is shown before and after stepwise model selection, while Mean Standard Prediction Error (MSPE) and pseudo-R^2^ are shown after selection*.

**Table 2:** Summary of the classification random forest models for categorical variables.

			**Before model selection**	**After model selection**		
**Data set**	**Variable**	**Taxonomic level**	***n* (predictors)**	***n* (predictors)**	**Cohen's Kappa**	**OOB Error (%)**
Spring 2014/2015	Site	Class	164	2	1	0
		Functional mapping	48	11	0.9	7.14
	Lake	Phylum	54	1	1	0
		Functional mapping	48	1	1	0
	Sampling year	Class	164	2	0.91	3.57
		Functional mapping	48	3	0.81	7.14
Summer 2015/Archaea	Site	Phylum	8	3	1	0
		Functional mapping	10	1	1	0
Summer 2015/Bacteria	Site	Phylum	37	1	1	0
		Functional mapping	26	3	1	0

*Number of predictors (n) is shown before and after stepwise model selection, while Cohen's Kappa values and Out Of Bag (OOB) error rates are shown after selection*.

Taxonomic groups that increased in abundance with increasing redox potential were aerobic chemoheterotrophs, such as Acidobacteria (Ward et al., 2009), and obligate aerobic methylotrophic Betaproteobacteria (Chistoserdova and Lidstrom, 2013; Figure S18a). In addition, the functionally predicted group of methanol oxidizers increased in abundance with increasing redox, which suggests that these organisms are aerobic (Jenkins et al., 1987). However, putative sulfur reducers also showed a positive relationship with redox, which was a surprising result. Most of the taxa mapped with FAPROTAX to this functional group belong to the uncultured genus *Desulfurellaceae* H16, which has been previously detected in anaerobic bioreactors (Wei et al., 2017). Bacteria from the family Desulfurellaceae are typically strict anaerobic sulfur-reducers (Greene, 2014; Florentino et al., 2017), but here seem to be abundant at sites with high redox potential (>400 mV) and in the presence of oxygen (>4 mgL-1). To the best of our knowledge, this has not been observed in previous studies.

In the current study, we identified pH as an important driver of the sediment microbial community structure and diversity, similarly to previous studies (see SI text; Xiong et al., 2012). Random forest analysis showed that the relationships of taxonomic groups to variation in pH were mostly supportive of previous observations in lake sediments (see SI text; Figures S16, S27a; Xiong et al., 2012). We also detected an increased abundance of Cyanobacteria at higher pH (Figures S16, S27), which is in accordance to the generation of alkaline conditions via autotrophic pathways. Similar relationships between pH and Cyanobacteria in the High Arctic have been previously observed in lake microbial mats (Lionard et al., 2012). We also observed a higher abundance of functionally predicted sulfate respirers and methanogens at lower pH. This is in accordance with lower pH optimums of these processes (Ferry, 1993; Hao et al., 1996), than the average pH of 7-8 in our samples.

Finally, results from the random forest analysis showed that abundances of predicted fermenters and intracellular parasites (most of these are known as Amoebae-Resistant Microbes; Greub and Raoult, 2004) increase with water depth (Figure S20b). The OTUs identified in our analysis included representatives of, e.g., phylum Chlamydiae (Lory, 2014), and orders Legionnellales (Garrity et al., 2015) and Rickettsiales (Renvoisé et al., 2011). The presence of obligate intracellular parasites indicates a higher abundance of grazing protists, and in the case of Rickettsiales, of arthropods (Renvoisé et al., 2011) at the deeper sites. These organisms might together with fermenting microbes contribute to increased cycling of organic matter and transfer of energy to higher trophic levels (Lei et al., 2014). The increased abundance of microbes involved in organic matter cycling suggests increased delivery of material to the deep basin (i.e., sediment focusing) in Lake Hazen. Furthermore, the longer duration of ice-free periods (Latifovic and Pouliot, 2007; Surdu et al., 2016) and increased runoff (Bliss et al., 2014) seem to have already increased the sediment, carbon, and nutrient inputs to Lake Hazen (Lehnherr et al., 2018).

## Conclusions

Despite extreme conditions in the High Arctic, our results show that lake sediments from this area harbor highly diverse microbial communities that vary both in time and space, but that are mainly shaped by redox and pH. Although the microbial communities in cores sampled at the three sites in Lake Hazen were phylogenetically distinct, they were functionally predicted to exhibit similarities. However, such functional predictions need now to be validated with metagenomics or metatranscriptomics studies, especially when performed on undersampled and extreme environments such as Lake Hazen.

The way such extreme environments will behave in the context of climate change is unclear. On the one hand, the predicted functional similarity of the communities in the backdrop of spatiotemporal microbial heterogeneity could be interpreted as a sign of resilience. However, as rising temperatures have both direct and indirect influences on redox chemistry and pH, the main drivers of microbial communities identified herein, it is very plausible that the current community structure could be disrupted under the climate regime predicted for the Arctic. Future work on Arctic lake sediments should focus on elucidating the functioning of the communities, and long-term studies performed throughout the seasonal regime shifts. As these seasonal shifts drive the redox chemistry, light and nutrient availability in the lakes, they might also affect the structure of microbial communities within.

## Data availability

All analysis scripts generated for this study can be found in the GitHub repository (https://github.com/Begia/Hazen16S), sequencing data is deposited in the NCBI Sequence Read Archive (https://www.ncbi.nlm.nih.gov/bioproject/PRJNA430127), and the geochemical data is deposited in the the NOAA National Centers for Environmental Information database (http://accession.nodc.noaa.gov/0171496).

## Author contributions

MR, AP and VS designed the experiments. MR, KS and VS performed the sampling. KS and VS produced the physicochemical data. MR extracted DNA, analyzed the data and prepared the manuscript, with extensive contributions from all coauthors. SA-B and AP supervised the project.

### Conflict of interest statement

The authors declare that the research was conducted in the absence of any commercial or financial relationships that could be construed as a potential conflict of interest.

## References

[B1] Aris-Brosou S. Rodrigue N. (2012). The essentials of computational molecular evolution, in Evolutionary Genomics Methods in Molecular Biology, ed Anisimova M. (Totowa, NJ: Humana Press), 111–152. 10.1007/978-1-61779-582-4_4 22407707

[B2] Aßhauer K. P. Wemheuer B. Daniel R. Meinicke P. (2015). Tax4Fun: predicting functional profiles from metagenomic 16S rRNA data. Bioinformatics 31, 2882–2884. 10.1093/bioinformatics/btv287 25957349 PMC4547618

[B3] Beall B. F. Twiss M. R. Smith D. E. Oyserman B. O. Rozmarynowycz M. J. Binding C. E.. (2016). Ice cover extent drives phytoplankton and bacterial community structure in a large north-temperate lake: implications for a warming climate: effect of ice cover on microbial community structure. Environ. Microbiol. 18, 1704–1719. 10.1111/1462-2920.12819 25712272

[B4] Bliss A. Hock R. Radić V. (2014). Global response of glacier runoff to twenty-first century climate change. J. Geophys. Res. Earth Surf. 119, 717–730. 10.1002/2013JF002931

[B5] Booth I. R. (1985). Regulation of cytoplasmic pH in bacteria. Microbiol. Rev. 49, 359–378. 3912654 10.1128/mr.49.4.359-378.1985 PMC373043

[B6] Breiman L. (2001). Random forests. Mach. Learn. 45, 5–32. 10.1023/A:1010933404324

[B7] Brouwer H. Murphy T. (1995). Volatile sulfides and their toxicity in freshwater sediments. Environ. Toxicol. Chem. 14, 203–208. 10.1002/etc.5620140204

[B8] Buttigieg P. L. Ramette A. (2014). A guide to statistical analysis in microbial ecology: a community-focused, living review of multivariate data analyses. FEMS Microbiol. Ecol. 90, 543–550. 10.1111/1574-6941.12437 25314312

[B9] Cadillo-Quiroz H. Yavitt J. B. Zinder S. H. (2009). Methanosphaerula palustris gen. nov., sp. nov., a hydrogenotrophic methanogen isolated from a minerotrophic fen peatland. Int. J. Syst. Evol. Microbiol. 59, 928–935. 10.1099/ijs.0.006890-0 19406770

[B10] Campello R. J. G. B. Moulavi D. Sander J. (2013). Density-based clustering based on hierarchical density estimates, in Advances in Knowledge Discovery and Data Mining Lecture Notes in Computer Science, eds Pei J. Tseng V. S. Cao L. Motoda H. Xu G. (Berlin; Heidelberg: Springer), 160–172.

[B11] Capella-Gutiérrez S. Silla-Martínez J. M. Gabaldón T. (2009). trimAl: a tool for automated alignment trimming in large-scale phylogenetic analyses. Bioinformatics 25, 1972–1973. 10.1093/bioinformatics/btp348 19505945 PMC2712344

[B12] Carini P. Marsden P. J. Leff J. W. Morgan E. E. Strickland M. S. Fierer N. (2017). Relic DNA is abundant in soil and obscures estimates of soil microbial diversity. Nat. Microbiol. 2:16242. 10.1038/nmicrobiol.2016.242 27991881

[B13] Chistoserdova L. Lidstrom M. E. (2013). Aerobic methylotrophic prokaryotes, in The Prokaryotes, eds Rosenberg E. DeLong E. F. Lory S. Stackebrandt E. Thompson F. (Berlin; Heidelberg: Springer), 267–285.

[B14] DeAngelis K. M. Silver W. L. Thompson A. W. Firestone M. K. (2010). Microbial communities acclimate to recurring changes in soil redox potential status. Environ. Microbiol. 12, 3137–3149. 10.1111/j.1462-2920.2010.02286.x 20629704

[B15] Drevnick P. E. Muir D. C. G. Lamborg C. H. Horgan M. J. Canfield D. E. Boyle J. F.. (2010). Increased accumulation of sulfur in lake sediments of the High Arctic. Environ. Sci. Technol. 44, 8415–8421. 10.1021/es101991p 20973547

[B16] Edgar R. C. Haas B. J. Clemente J. C. Quince C. Knight R. (2011). UCHIME improves sensitivity and speed of chimera detection. Bioinformatics 27, 2194–2200. 10.1093/bioinformatics/btr381 21700674 PMC3150044

[B17] Emerson J. B. Varner R. K. Johnson J. E. Owusu-Dommey A. Binder M. Woodcroft B. J.. (2015). Linking sediment microbial communities to carbon cycling in high-latitude lakes. *AGU Fall Meet. Abstr*. 21. Available online at: http://adsabs.harvard.edu/abs/2015AGUFM.B21C0454E

[B18] Emmerton C. A. St. Louis V. L. Lehnherr I. Graydon J. A. Kirk J. L. Rondeau K. J. (2016). The importance of freshwater systems to the net atmospheric exchange of carbon dioxide and methane with a rapidly changing high Arctic watershed. Biogeosciences 13, 5849–5863. 10.5194/bg-13-5849-2016

[B19] Ferry J. G. (1993). Methanogenesis: Ecology, Physiology, Biochemistry and Genetics. Dordrecht: Springer Science and Business Media.

[B20] Florentino A. P. Stams A. J. M. Sánchez-Andrea I. (2017). Genome sequence of *Desulfurella amilsii* strain TR1 and comparative genomics of Desulfurellaceae family. Front. Microbiol. 8:222. 10.3389/fmicb.2017.00222 28265263 PMC5317093

[B21] Fukuyama J. McMurdie P. J. Dethlefsen L. Relman D. A. Holmes S. (2012). Comparisons of distance methods for combining covariates and abundances in microbiome studies. Pac. Symp. Biocomput. 213–224. 10.1142/9789814366496_0021 22174277 PMC4532668

[B22] Garrity G. M. Bell J. A. Lilburn T. (2015). Legionellales ord. nov, in Bergey's Manual of Systematics of Archaea and Bacteria, eds Whitman W. B. Rainey F. Kämpfer P. Trujillo M. Chun J. DeVos P.. (Chichester, UK: John Wiley and Sons, Ltd.), 1–1.

[B23] Ge Y. He J. Zhu Y. Zhang J. Xu Z. Zhang L.. (2008). Differences in soil bacterial diversity: driven by contemporary disturbances or historical contingencies? ISME J. 2, 254–264. 10.1038/ismej.2008.2 18239609

[B24] Gilmour C. C. Podar M. Bullock A. L. Graham A. M. Brown S. D. Somenahally A. C.. (2013). Mercury methylation by novel microorganisms from new environments. Environ. Sci. Technol. 47, 11810–11820. 10.1021/es403075t 24024607

[B25] Glassing A. Dowd S. E. Galandiuk S. Davis B. Chiodini R. J. (2016). Inherent bacterial DNA contamination of extraction and sequencing reagents may affect interpretation of microbiota in low bacterial biomass samples. Gut Pathog. 8:24. 10.1186/s13099-016-0103-7 27239228 PMC4882852

[B26] Greene A. C. (2014). The family Desulfurellaceae, in The Prokaryotes, eds Rosenberg E. DeLong E. F. Lory S. Stackebrandt E. Thompson F. (Berlin, Heidelberg: Springer Berlin Heidelberg), 135–142.

[B27] Greub G. Raoult D. (2004). Microorganisms resistant to free-living amoebae. Clin. Microbiol. Rev. 17, 413–433. 10.1128/CMR.17.2.413-433.2004 15084508 PMC387402

[B28] Hahsler M. Piekenbrock M. Arya S. Mount D. (2017). dbscan: Density Based Clustering of Applications with Noise (DBSCAN) and Related Algorithms. Available online at: https://CRAN.R-project.org/package=dbscan (Accessed November 29, 2017).

[B29] Hao O. J. Chen J. M. Huang L. Buglass R. L. (1996). Sulfate-reducing bacteria. Crit. Rev. Environ. Sci. Technol. 26, 155–187. 10.1080/10643389609388489

[B30] Hauptmann A. L. Markussen T. N. Stibal M. Olsen N. S. Elberling B. Bælum J.. (2016). Upstream freshwater and terrestrial sources are differentially reflected in the bacterial community structure along a small Arctic river and its estuary. Front. Microbiol. 7:1474. 10.3389/fmicb.2016.01474 27708629 PMC5030300

[B31] Hoppe H. Gocke K. Kuparinen J. (1990). Effect of H2S on heterotrophic substrate uptake, extracellular enzyme activity and growth of brackish water bacteria. Mar. Ecol. Prog. Ser. 64, 157–167. 10.3354/meps064157

[B32] Horikoshi M. Tang Y. (2017). ggfortify: Data Visualization Tools for Statistical Analysis Results. Available online at: https://cran.r-project.org/web/packages/ggfortify/index.html.

[B33] Jenkins O. Byrom D. Jones D. (1987). Methylophilus: a new genus of methanol-utilizing bacteria. Int. J. Syst. Evol. Microbiol. 37, 446–448. 10.1099/00207713-37-4-446

[B34] Jones Z. Linder F. (2016). edarf: exploratory data analysis using random forests. J. Open Source Softw. 1:92. 10.21105/joss.00092

[B35] Keatley B. E. Douglas M. S. V. Smol J. P. (2007). Limnological characteristics of a High Arctic oasis and comparisons across northern Ellesmere Island. Arctic 60, 294–308. 10.14430/arctic221

[B36] Klatt J. M. Haas S. Yilmaz P. de Beer D. Polerecky L. (2015). Hydrogen sulfide can inhibit and enhance oxygenic photosynthesis in a cyanobacterium from sulfidic springs. Environ. Microbiol. 17, 3301–3313. 10.1111/1462-2920.12791 25630511

[B37] Klein D. A. (2007). Microbial communities in nature: a postgenomic perspective. Microbe Mag. 2, 591–595. 10.1128/microbe.2.591.1

[B38] Köck G. Muir D. Yang F. Wang X. Talbot C. Gantner N.. (2012). Bathymetry and sediment geochemistry of Lake Hazen (Quttinirpaaq National Park, Ellesmere Island, Nunavut). Arctic 56–66. 10.14430/arctic4165

[B39] Kokelj S. V. Jenkins R. E. Milburn D. Burn C. R. Snow N. (2005). The influence of thermokarst disturbance on the water quality of small upland lakes, Mackenzie Delta region, Northwest Territories, Canada. Permafr. Periglac. Process. 16, 343–353. 10.1002/ppp.536

[B40] Krijthe J. van der Maaten L. (2017). rtsne: T-Distributed Stochastic Neighbor Embedding Using a Barnes-Hut Implementation. Available online at: https://cran.r-project.org/web/packages/Rtsne/index.html (Accessed November 29, 2017).

[B41] Kuang J.-L. Huang L.-N. Chen L.-X. Hua Z.-S. Li S.-J. Hu M.. (2013). Contemporary environmental variation determines microbial diversity patterns in acid mine drainage. ISME J. 7, 1038–1050. 10.1038/ismej.2012.139 23178673 PMC3635239

[B42] Kuczynski J. Stombaugh J. Walters W. A. González A. Caporaso J. G. Knight R. (2011). Using QIIME to analyze 16S rRNA gene sequences from microbial communities. Curr. Protoc. Bioinformatics. Ed. Board Andreas Baxevanis Al CHAPTER, Unit10.7. 10.1002/0471250953.bi1007s36 PMC3249058 22161565

[B43] Lamarche-Gagnon G. Comery R. Greer C. W. Whyte L. G. (2015). Evidence of in situ microbial activity and sulphidogenesis in perennially sub-0 °C and hypersaline sediments of a high Arctic permafrost spring. Extremophiles 19, 1–15. 10.1007/s00792-014-0703-4 25381577

[B44] Langille M. G. I. Zaneveld J. Caporaso J. G. McDonald D. Knights D. Reyes J. A.. (2013). Predictive functional profiling of microbial communities using 16S rRNA marker gene sequences. Nat. Biotechnol. 31, 814–821. 10.1038/nbt.2676 23975157 PMC3819121

[B45] Latifovic R. Pouliot D. (2007). Analysis of climate change impacts on lake ice phenology in Canada using the historical satellite data record. Remote Sens. Environ. 106, 492–507. 10.1016/j.rse.2006.09.015

[B46] Lehnherr I. St. Louis V. L. Kirk J. L. (2012). Methylmercury cycling in high arctic wetland ponds: controls on sedimentary production. Environ. Sci. Technol. 46, 10523–10531. 10.1021/es300577e 22799567

[B47] Lehnherr I. St. Louis V. L. Sharp M. Gardner A. S. Smol J. P. Schiff S. L.. (2018). The world's largest High Arctic lake responds rapidly to climate warming. Nat. Commun. 9:1290. 10.1038/s41467-018-03685-z 29599477 PMC5876346

[B48] Lei Y.-L. Stumm K. Wickham S. A. Berninger U.-G. (2014). Distributions and biomass of benthic ciliates, foraminifera and amoeboid protists in marine, brackish, and freshwater sediments. J. Eukaryot. Microbiol. 61, 493–508. 10.1111/jeu.12129 24919761

[B49] Liaw A. Wiener M. (2002). Classification and regression by randomforest. R News 2, 18–22.

[B50] Lionard M. Péquin B. Lovejoy C. Vincent W. F. (2012). Benthic cyanobacterial mats in the High Arctic: multi-layer structure and fluorescence responses to osmotic stress. Front. Microbiol. 3:140. 10.3389/fmicb.2012.00140 22557996 PMC3337508

[B51] Lory S. (2014). The Phylum Chlamydiae, in The Prokaryotes, eds Rosenberg E. DeLong E. F. Lory S. Stackebrandt E. Thompson F. (Berlin; Heidelberg: Springer), 497–499.

[B52] Louca S. Jacques S. M. S. Pires A. P. F. Leal J. S. Srivastava D. S. Parfrey L. W.. (2016a). High taxonomic variability despite stable functional structure across microbial communities. Nat. Ecol. Evol. 1:0015. 10.1038/s41559-016-0015 28812567

[B53] Louca S. Parfrey L. W. Doebeli M. (2016b). Decoupling function and taxonomy in the global ocean microbiome. Science 353, 1272–1277. 10.1126/science.aaf4507 27634532

[B54] Lozupone C. A. Knight R. (2007). Global patterns in bacterial diversity. Proc. Natl. Acad. Sci. U.S.A. 104, 11436–11440. 10.1073/pnas.0611525104 17592124 PMC2040916

[B55] Lundin D. Severin I. Logue J. B. Östman Ö. Andersson A. F. Lindström E. S. (2012). Which sequencing depth is sufficient to describe patterns in bacterial α- and β-diversity? Environ. Microbiol. Rep. 4, 367–372. 10.1111/j.1758-2229.2012.00345.x 23760801

[B56] Mahé F. Rognes T. Quince C. de Vargas C. Dunthorn M. (2015). Swarm v2: highly-scalable and high-resolution amplicon clustering. PeerJ 3:e1420. 10.7717/peerj.1420 26713226 PMC4690345

[B57] McMurdie P. J. Holmes S. (2013). phyloseq: an R package for reproducible interactive analysis and graphics of microbiome census data. PLoS ONE 8:e61217. 10.1371/journal.pone.0061217 23630581 PMC3632530

[B58] Menze B. H. Kelm B. M. Masuch R. Himmelreich U. Bachert P. Petrich W.. (2009). A comparison of random forest and its Gini importance with standard chemometric methods for the feature selection and classification of spectral data. BMC Bioinformatics 10:213. 10.1186/1471-2105-10-213 19591666 PMC2724423

[B59] Mesquita P. S. Wrona F. J. Prowse T. D. (2010). Effects of retrogressive permafrost thaw slumping on sediment chemistry and submerged macrophytes in Arctic tundra lakes. Freshw. Biol. 55, 2347–2358. 10.1111/j.1365-2427.2010.02450.x

[B60] Mohit V. Culley A. Lovejoy C. Bouchard F. Vincent W. F. (2017). Hidden biofilms in a far northern lake and implications for the changing Arctic. NPJ Biofilms Microbiomes 3:17. 10.1038/s41522-017-0024-3 28702216 PMC5500582

[B61] Morris E. K. Caruso T. Buscot F. Fischer M. Hancock C. Maier T. S.. (2014). Choosing and using diversity indices: insights for ecological applications from the German biodiversity exploratories. Ecol. Evol. 4, 3514–3524. 10.1002/ece3.1155 25478144 PMC4224527

[B62] Mueller D. R. Van Hove P. Antoniades D. Jeffries M. O. Vincent W. F. (2009). High Arctic lakes as sentinel ecosystems: cascading regime shifts in climate, ice cover, and mixing. Limnol. Oceanogr. 54, 2371–2385. 10.4319/lo.2009.54.6_part_2.2371

[B63] Nemergut D. R. Schmidt S. K. Fukami T. O'Neill S. P. Bilinski T. M. Stanish L. F.. (2013). Patterns and processes of microbial community assembly. Microbiol. Mol. Biol. Rev. MMBR 77, 342–356. 10.1128/MMBR.00051-12 24006468 PMC3811611

[B64] Okonechnikov K. Golosova O. Fursov M. The UGENE Team (2012). Unipro UGENE: a unified bioinformatics toolkit. Bioinformatics 28, 1166–1167. 10.1093/bioinformatics/bts091 22368248

[B65] Oksanen J. Blanchet F. G. Friendly M. Kindt R. Legendre P. McGlinn D.. (2016). vegan: Community Ecology Package. Available online at: https://CRAN.R-project.org/package=vegan

[B66] Ortiz-Alvarez R. Casamayor E. O. (2016). High occurrence of Pacearchaeota and Woesearchaeota (Archaea superphylum DPANN) in the surface waters of oligotrophic high-altitude lakes. Environ. Microbiol. Rep. 8, 210–217. 10.1111/1758-2229.12370 26711582

[B67] Pasolli E. Truong D. T. Malik F. Waldron L. Segata N. (2016). Machine learning meta-analysis of large metagenomic datasets: tools and biological insights. PLoS Comput. Biol. 12:e1004977. 10.1371/journal.pcbi.1004977 27400279 PMC4939962

[B68] Paulson J. N. Stine O. C. Bravo H. C. Pop M. (2013). Robust methods for differential abundance analysis in marker gene surveys. Nat. Methods 10, 1200–1202. 10.1038/nmeth.2658 24076764 PMC4010126

[B69] Pavoine S. Dufour A. B. Chessel D. (2004). From dissimilarities among species to dissimilarities among communities: a double principal coordinate analysis. J. Theor. Biol. 228, 523–537. 10.1016/j.jtbi.2004.02.014 15178200

[B70] Pinto A. J. Raskin L. (2012). PCR biases distort bacterial and archaeal community structure in pyrosequencing datasets. PLoS ONE 7:e43093. 10.1371/journal.pone.0043093 22905208 PMC3419673

[B71] Poulain A. J. Aris-Brosou S. Blais J. M. Brazeau M. Keller W. B. Paterson A. M. (2015). Microbial DNA records historical delivery of anthropogenic mercury. ISME J. 9, 2541–2550. 10.1038/ismej.2015.86 26057844 PMC4817628

[B72] Price M. N. Dehal P. S. Arkin A. P. (2010). FastTree 2 – Approximately maximum-likelihood trees for large alignments. PLoS ONE 5:e9490. 10.1371/journal.pone.0009490 20224823 PMC2835736

[B73] Pruesse E. Peplies J. Glöckner F. O. (2012). SINA: accurate high-throughput multiple sequence alignment of ribosomal RNA genes. Bioinformatics 28, 1823–1829. 10.1093/bioinformatics/bts252 22556368 PMC3389763

[B74] Quast C. Pruesse E. Yilmaz P. Gerken J. Schweer T. Yarza P.. (2013). The SILVA ribosomal RNA gene database project: improved data processing and web-based tools. Nucleic Acids Res. 41, D590–D596. 10.1093/nar/gks1219 23193283 PMC3531112

[B75] R Core Team (2017). R: A Language and Environment for Statistical Computing. Vienna: R Foundation for Statistical Computing. Available online at: https://www.R-project.org/.

[B76] Reist J. D. Gyselman E. Babaluk J. A. Johnson J. D. Wissink R. (1995). Evidence for two morphotypes of Arctic char (Salvelinus alpinus (L.)) from Lake Hazen, Ellesmere Island, Northwest Territories, Canada. Nord. J. Freshw. Res. 71, 396–410

[B77] Renvoisé A. Merhej V. Georgiades K. Raoult D. (2011). Intracellular rickettsiales: insights into manipulators of eukaryotic cells. Trends Mol. Med. 17, 573–583. 10.1016/j.molmed.2011.05.009 21763202

[B78] Rognes T. Flouri T. Nichols B. Quince C. Mahé F. (2016). VSEARCH: a versatile open source tool for metagenomics. PeerJ 4:e2584. 10.7717/peerj.2584 27781170 PMC5075697

[B79] Salter S. J. Cox M. J. Turek E. M. Calus S. T. Cookson W. O. Moffatt M. F.. (2014). Reagent and laboratory contamination can critically impact sequence-based microbiome analyses. BMC Biol. 12:87. 10.1186/s12915-014-0087-z 25387460 PMC4228153

[B80] Schütte U. M. Cadieux S. B. Hemmerich C. Pratt L. M. White J. R. (2016). Unanticipated geochemical and microbial community structure under seasonal ice cover in a dilute, dimictic Arctic lake. Front. Microbiol. 7:1035. 10.3389/fmicb.2016.01035 27458438 PMC4932660

[B81] Stoeva M. K. Aris-Brosou S. Chételat J. Hintelmann H. Pelletier P. Poulain A. J. (2014). Microbial community structure in lake and wetland sediments from a High Arctic polar desert revealed by targeted transcriptomics. PLoS ONE 9:e89531. 10.1371/journal.pone.0089531 24594936 PMC3940601

[B82] Sun W. Xiao E. Xiao T. Krumins V. Wang Q. Häggblom M.. (2017). Response of soil microbial communities to elevated antimony and arsenic contamination indicates the relationship between the innate microbiota and contaminant fractions. Environ. Sci. Technol. 51, 9165–9175. 10.1021/acs.est.7b00294 28700218

[B83] Surdu C. M. Duguay C. R. Fernández Prieto D. (2016). Evidence of recent changes in the ice regime of lakes in the Canadian High Arctic from spaceborne satellite observations. Cryosphere 10, 941–960. 10.5194/tc-10-941-2016

[B84] Tang C. Madigan M. T. Lanoil B. (2013). Bacterial and Archaeal diversity in sediments of West Lake Bonney, McMurdo Dry Valleys, Antarctica. Appl. Environ. Microbiol. 79, 1034–1038. 10.1128/AEM.02336-12 23183970 PMC3568539

[B85] Thaler M. Vincent W. F. Lionard M. Hamilton A. K. Lovejoy C. (2017). Microbial community structure and interannual change in the last epishelf lake ecosystem in the north Polar region. Front. Mar. Sci. 3:275. 10.3389/fmars.2016.00275

[B86] Thomas F. Hehemann J.-H. Rebuffet E. Czjzek M. Michel G. (2011). Environmental and gut bacteroidetes: the food connection. Front. Microbiol. 2:93. 10.3389/fmicb.2011.00093 21747801 PMC3129010

[B87] Touw W. G. Bayjanov J. R. Overmars L. Backus L. Boekhorst J. Wels M.. (2013). Data mining in the life sciences with random forest: a walk in the park or lost in the jungle? Brief. Bioinformatics 14, 315–326. 10.1093/bib/bbs034 22786785 PMC3659301

[B88] van der Maaten L. Hinton G. (2008). Visualizing data using t-SNE. J. Mach. Learn. Res. 9, 2579–2605.

[B89] Vincent W. F. Laybourn-Parry J. (2008). Polar Lakes and Rivers: Limnology of Arctic and Antarctic Aquatic Ecosystems. Oxford, UK: Oxford University Press.

[B90] Wang N. F. Zhang T. Yang X. Wang S. Yu Y. Dong L. L.. (2016). Diversity and composition of bacterial community in soils and lake sediments from an arctic lake area. Front. Microbiol. 7:1170. 10.3389/fmicb.2016.01170 27516761 PMC4963411

[B91] Ward N. L. Challacombe J. F. Janssen P. H. Henrissat B. Coutinho P. M. Wu M.. (2009). Three genomes from the phylum Acidobacteria provide insight into the lifestyles of these microorganisms in soils. Appl. Environ. Microbiol. 75, 2046–2056. 10.1128/AEM.02294-08 19201974 PMC2663196

[B92] Wei H. Wang J. Hassan M. Han L. Xie B. (2017). Anaerobic ammonium oxidation-denitrification synergistic interaction of mature landfill leachate in aged refuse bioreactor: variations and effects of microbial community structures. Bioresour. Technol. 243, 1149–1158. 10.1016/j.biortech.2017.07.077 28764129

[B93] Wright M. N. Ziegler A. (2015). ranger: a fast implementation of random forests for high dimensional data in C++ and R. ArXiv150804409 Stat.

[B94] Wrighton K. C. Thomas B. C. Sharon I. Miller C. S. Castelle C. J. VerBerkmoes N. C.. (2012). Fermentation, hydrogen, and sulfur metabolism in multiple uncultivated bacterial phyla. Science 337, 1661–1665. 10.1126/science.1224041 23019650

[B95] Xiong J. Liu Y. Lin X. Zhang H. Zeng J. Hou J.. (2012). Geographic distance and pH drive bacterial distribution in alkaline lake sediments across Tibetan Plateau. Environ. Microbiol. 14, 2457–2466. 10.1111/j.1462-2920.2012.02799.x 22676420 PMC3477592

[B96] Xu Z. Malmer D. Langille M. G. I. Way S. F. Knight R. (2014). Which is more important for classifying microbial communities: who's there or what they can do? ISME J. 8, 2357–2359. 10.1038/ismej.2014.157 25171332 PMC4260698

[B97] Zhang J. Kobert K. Flouri T. Stamatakis A. (2014a). PEAR: a fast and accurate Illumina Paired-End reAd mergeR. Bioinformatics 30, 614–620. 10.1093/bioinformatics/btt593 24142950 PMC3933873

[B98] Zhang J. Yang Y. Zhao L. Li Y. Xie S. Liu Y. (2014b). Distribution of sediment Bacterial and Archaeal communities in plateau freshwater lakes. Appl. Microbiol. Biotechnol. 99, 3291–3302. 10.1007/s00253-014-6262-x 25432677

[B99] Zhou J. Bruns M. A. Tiedje J. M. (1996). DNA recovery from soils of diverse composition. Appl. Environ. Microbiol. 62, 316–322. 8593035 10.1128/aem.62.2.316-322.1996 PMC167800

