# Supplementary material for: Physicochemical Drivers of Microbial Community Structure in Sediments of Lake Hazen, Nunavut, Canada
Source: Front Microbiol. 2018 Jun 5;9:1138. doi: 10.3389/fmicb.2018.01138 (PMC5996194; doi:10.3389/fmicb.2018.01138)
Supplement: Supplementary file 1 [file Data_Sheet_1.DOCX]

Physicochemical drivers of sediment microbial community structure in Lake Hazen, Nunavut, Canada

Supplementary material

Matti O. Ruuskanen^1^*, Kyra A. St. Pierre^2^, Vincent L. St. Louis^2^, Stéphane Aris-Brosou^1^,^3^ and Alexandre J. Poulain^1^

^1^Department of Biology, University of Ottawa, Ottawa, ON, Canada,
^2^Department of Biological Sciences, University of Alberta, Edmonton, AB, Canada,
^3^Department of Mathematics & Statistics, University of Ottawa, Ottawa, ON, Canada

Table of contents

[1 Supplementary text 2](#_Toc510980250)

[1.1 Sequencing details 2](#_Toc510980251)

[1.2 Assessing contamination from DNA extraction kits 2](#_Toc510980252)

[1.3 Overview of the microbial community structure 3](#_Toc510980253)

[1.4 Taxonomic and functionally predicted group abundances along physicochemical gradients 5](#_Toc510980254)

[1.4.1 Continuous variables (regression rf) 6](#_Toc510980255)

[1.4.2 Categorical variables (classification rf) 8](#_Toc510980256)

[2 Supplementary figures and tables 10](#_Toc510980257)

[2.1 Sediment samples 10](#_Toc510980258)

[2.2 Data analysis quality control 15](#_Toc510980259)

[2.3 Functional mapping 20](#_Toc510980260)

[2.4 Alpha- and beta-diversity 22](#_Toc510980261)

[2.5 Partial dependence plots of gradient analysis random forests 27](#_Toc510980262)

[2.5.1 Continuous variables: spring 2014/2015 27](#_Toc510980263)

[2.5.2 Continuous variables: summer 2015 29](#_Toc510980264)

[2.5.3 Categorical variables: spring 2014/2015 34](#_Toc510980265)

[2.5.4 Categorical variables: summer 2015 35](#_Toc510980266)

[References 36](#_Toc510980267)

# 1 Supplementary text

## 1.1 Sequencing details

For spring 2014/2015 samples, the V3-V4 region of the bacterial 16S rRNA gene was sequenced in two separate runs using the primers 341F (5’-CCT ACG GGN GG CWG CAG-3’) and 805R (5’-GAC TAC HVG GGT ATC TAA TCC-3’; Herlemann et al., 2011). The use of these primers is recommended for environmental studies (Klindworth et al., 2013). Summer 2015 samples were sequenced for the V3-V5 region of the archaeal 16S with the primers Arch349F (5’-GYG CAS CAG KCG MGA AW-3’) and Arch806R (5’-GGA CTA CVS GGG TAT CTA AT-3’; Takai and Horikoshi, 2000). The same samples were also sequenced for the V1-V3 region of the bacterial 16S with primers 27Fmod (5’-AGR GTT TGA TCM TGG CTC AG-3’; Vergin et al., 1998) and 519Rmodbio (5’-GTN TTA CNG CGG CKG CTG-3’; Andreotti et al., 2011). The primers for each sequencing were used in a 28 cycle PCR with barcodes on the forward primer, using the HotStarTaq Plus Master Mix Kit (Qiagen, Germantown, MD, USA). Temperature cycling in PCR consisted of: 94°C, 3 min; 28 cycles of {94°C, 30 s; 53°C, 40 s; 72°C, 1 min}, with a final elongation at 72°C for 5 min. The PCR products were then checked in a 2% agarose gel and pooled in equal proportions based on the molecular weights and DNA concentrations of the products, followed by purification with calibrated Ampure XP beads (Beckman Coulter, CA, USA). All sequencing was performed on the Illumina MiSeq platform producing paired-end 300 bp reads according to the manufacturer’s guidelines, by Molecular Research LP (Shallowater, TX, USA).

## 1.2  Assessing contamination from DNA extraction kits

Briefly, putative contaminating genera from MOBIO PowerSoil kit (Glassing et al., 2016) and four other extraction kits (Salter et al., 2014) not used in this study were identified from the assigned taxonomy of the OTUs in all our data (Figure S4). The complete data analysis script was then run with either 100% of the putative MOBIO PowerSoil contaminants or 10%, 20%, 50% and 100% of abundances of all the putative contaminants removed from the OTU tables. None of our primary conclusions (from analysis of alpha- and beta-diversity, tSNE clustering, and differential analyses of taxa and functionally predicted groups along physicochemical gradients) were affected by removal of 100% of the putative MOBIO PowerSoil kit contaminants (Figures S5, S6). Furthermore, no effects were observed when 10% of abundances of all the putative contaminant genera were removed, but at 20% reduced abundance, the clustering patterns and physicochemical gradient analyses were visibly affected (data not shown). Beta-diversity analyses were more robust to reducing the abundances up to 20%, but at 50% reduced abundance water depth did not significantly correlate with the phylogenetic differences in the spring 2014/2015 data set. Results of our alpha-diversity analyses were not affected by even 100% removal of all the putative contaminant genera (data not shown).

## 1.3 Overview of the microbial community structure

Lake Hazen sediments in spring 2014/2015 were dominated by Proteobacteria (38%; Figure [1](#fig:Relative-abundances)). The largest Proteobacterial classes in Lake Hazen were Alphaproteobacteria (12%) and Betaproteobacteria (11%). Other major phyla were Bacteroidetes (10%), Acidobacteria (8%), Chloroflexi (7%), and Actinobacteria (7%). The most abundant archaeal phyla in Lake Hazen sediments was Woesearchaeota (formerly “DHVEG-6”; 0.2%, including bacteria in the total), and Thaumarchaeota (0.02%). Skeleton Lake sediments were also dominated by Proteobacteria (32%), but the classes differed from those in Lake Hazen: Deltaproteobacteria (9%), Gammaproteobacteria (9%) and Alphaproteobacteria (8%). Other major phyla in Skeleton Lake sediments were Chloroflexi (12%), Actinobacteria (11%), Bacteroidetes (8%), Planctomycetes (6%), and Cyanobacteria (6%). The most abundant archaeal phyla in Skeleton Lake sediments were Woesearchaeota (1%, including bacteria), followed by Euryarchaeota (0.9%).

In the summer 2015 sediment samples from Skeleton Lake, the archaeal community primarily consisted of Woesearchaeota (67%), Euryarchaeota (17%) and the Miscellaneous Euryarchaeotic Group (MEG; 16%; Figure [2](#fig:Relative-abundances)). The Pond1 archaeal community mostly consisted of Euryarchaeota (47%), Woesearchaeota (32%), MEG (12%), and Thaumarchaeota (6%). The most abundant bacterial phyla in the 2015 summer Skeleton Lake sediments were Chloroflexi (20%), Bacteroidetes (20%), Proteobacteria (16%, in order of abundance: Beta-, Alpha-, Delta-, and Gamma-), Cyanobacteria (9%), Gracilibacteria (8%), and SR1 (Absconditabacteria; 5%; Figure [2](#fig:Relative-abundances)). The most abundant bacterial phyla in Pond1 sediments were Chloroflexi (24%), Proteobacteria (18%, in order of abundance: Alpha-, Delta-, Beta- and Gamma-), Bacteroidetes (15%), Cyanobacteria (15%), and Firmicutes (6%).

The most common functionally mapped groups in Lake Hazen in spring 2014/2015 were aerobic chemoheterotrophs (while ranking second in Skeleton Lake), and cyanobacteria (as they are grouped together at phylum level in FAPROTAX) in Skeleton Lake in spring 2014/2015 (Figure S8). Besides cyanobacteria, sulfate reducers were the second most common functionally mapped group in Skeleton Lake sediments, while almost absent from sediments in Lake Hazen. Finally, aerobic ammonia and nitrite oxidizers, as well as intracellular parasites were more prevalent in Lake Hazen than in Skeleton Lake sediments. Overall, functionally mapped groups associated with aerobic metabolism seemed to be more prevalent in Lake Hazen than in Skeleton Lake sediments.

Skeleton Lake and Pond1 had somewhat similar functional predictions, as methanogenesis dominated in both sediments (Figure S9). About 2/3 of the archaeal functional mapping groups were methanogenic; mercury methylators, and nitrogen fixing archaea were present at both sites. In the bacterial data from 2015, cyanobacteria, and aerobic chemoheterotrophs were the most abundant functional predictions.

1.4  Taxonomic and functionally predicted group abundances along physicochemical gradients

The best random forest models had pseudo-R^2^ values between -0.15 (*i.e.*, worse than fitting a straight line) and 0.97 with 1 to 16 predictors for continuous physicochemical variables (Figures S15−S32). For the models of categorical variables, the OOB error rates varied from 0% to 7.14% with 1 to 11 predictors (Figures S33−S37). Most of the taxonomic models had the best prediction accuracy on the order level (8 out of 20 models), which was the lowest taxonomic rank included. The predictions of taxonomic data were generally better than functionally mapped data for the same variable. The average improvement in MSPE from functionally mapped to taxonomic data was 66% for spring 2014/2015, 434% for summer 2015 archaeal data (improvement in the Cl^-^ model from 0.27 to 0.01 MSPE was highly influential) and 54% for summer 2015 bacterial data. The improvement was probably caused by the lower coverage of the functional mapping of the OTUs in the data since random forests usually perform better with more data. The high improvement in the archaeal data set was mostly caused by the difference between the two [Cl^-^] models. Highest pseudo-R2 values from the models were obtained for [H_2_S] (0.96−0.97, only spring 2014/2015), water depth (0.71−0.93, only spring 2014/2015), [NO_3_^-^] (0.87−0.90, only summer 2015), pH (0.48−0.88), sediment depth (0.40−0.86) and redox potential (0.74−0.79, only spring 2014/2015). All variables except [O_2_] in summer 2015 (pseudo-R^2^ ≤ 0), could be linked to changes in taxonomical and/or functional mapping group abundances along their gradients. Variability in the predictors explained by our regression random forest models was high for both taxonomic and functional mapping groups. The few most important groups in the models were selected among up to 280 taxonomic or up to 48 functional mapping groups. They also represented groups that we expected to have the strongest response to the variable in question.

Trends of observed relationships between phylogenetic and functional mapping groups to the most relevant physicochemical variables ([H_2_S], redox potential, pH, water depth, and [NO_3_-]) are described below, with further detail than in the main manuscript.

### 1.4.1 Continuous variables (regression rf)

Higher [H_2_S] was linked to lower abundance of 16 bacterial classes, *e.g.*, within Acidobacteria and Bacteroidetes in the spring 2014/2015 data set (Figure S15a). These taxa are primarily aerobic heterotrophs (for example (Kämpfer, 2015; Rapp et al., 2016; Ward et al., 2009)). The functional mapping showed a lower abundance of aerobic ammonia oxidizers, chemoheterotrophs and nitrite oxidizers, together with predatory or exoparasitic microbes in the spring 2014/2015 data set (Figure S15b). In the spring 2014/2015 functionally mapped data, higher [H_2_S] correlated with increasing abundances of cyanobacteria, methanogens, and sulfate respirers (Figure S15b).

In the spring 2014/2015 phylogenetic data, the abundance of 4 orders within the phylum Acidobacteria, the order Gemmatimonadales, and the orders Methylophilales and TRA3-20 within Betaproteobacteria increased with increasing redox potential (Figure S18a). However, the abundance of order Anaerolineales decreased with increasing redox (Figure S18a). In the spring 2014/2015 functionally mapped data, methanol oxidizers and sulfur respirers had positive trends with higher redox (Figure S18b). The only group decreased in abundance with increasing redox potential was the strictly anaerobic order Anaerolineales from the phylum Chloroflexi.

In the spring 2014/2015 phylogenetic data, increasing pH was linked with increasing abundance of the order Ignavibacteriales, and uncultured/unknown orders within phyla Omnitrophica and Verrucomicrobia (Figure S16a). Orders Frankiales, Coriobacteriales, Bacteriodales, Rhodobacterales, and Desulfuromonadales decreased in abundance with increasing pH in the spring 2014/2015 data set (Figure S16a). In the summer 2015 archaeal communities, the abundance of an uncultured class in phylum Woesearchaeota (DHVEG-6) increased with increasing pH (Figure S21a). An uncultured class within the Miscellaneous Euryarchaeotic Group (MEG) decreased in abundance with increasing pH (Figure S21a). In the bacterial communities, the abundances of phyla Cyanobacteria and Proteobacteria increased with increasing pH (Figure S27a). 17 phyla decreased in abundance with increasing pH, for example, Actinobacteria, Bacteroidetes, Chlorobi, Chloroflexi, Firmicutes, Gracilibacteria, Microgenomates, and Absconditabacteria (Figure S27a). Iron respirers, photoheterotrophs, and sulfate respirers decreased in abundance with increasing pH (Figure S16b). In summer 2015 archaeal data, abundances of mercury methylators were positively related to pH (Figure S21b). Also, several methanogenic groups had a negative relationship with pH (Figure S21b). In summer 2015 bacterial data, increasing pH was linked with increasing abundances of aerobic ammonia oxidizers, anoxygenic phototrophs and chemoheterotrophs, cyanobacteria, and ureolytic microbes (Figure S27b). However, sulfur oxidizers, fermenters, iron respiring and sulfate respiring microbes decreased in abundance with increasing pH (Figure S27b).

Our results correspond to previous results on the role of pH as a factor controlling lake sediment microbial community structure and diversity (Xiong et al., 2012). However, in the current study the abundance of an Alphaproteobacterial order declined towards higher pH (Figure S16a), contrary to previous observations (Xiong et al., 2012). Our results are also somewhat contrary to positive relationships of Actinobacteria and Bacteroidetes with pH in arctic soil (Chu et al., 2010). However, the discrepancies might be explained by the different pH ranges and taxonomic levels examined in these studies.

In the spring 2014/2015 data two classes, Subgroup 2 within phylum Acidobacteria, and Flavobacteria, had higher abundance at deeper sites (Figure S20a). From functional mapping groups, fermenters and intracellular parasites, also had positive relationship with water depth, while iron respirers displayed a U-shaped trend (either low or high abundance at deeper sites; Figure S20b).

Higher [NO_3_-] in the summer 2015 archaeal data was affiliated with increased abundance of 5 uncultured or unidentified orders within the Soil Crenarchaeotic Group, and decreased abundance of orders Methanomicrobiales, Methanosarcinales, and Thermoplasmatales (Figure S26a). In the summer 2015 archaeal data the abundances of an uncultured Bacteroidetes order, and the orders Chlorobiales, Fibrobacterales, Burkholderiales, and Desufovibrionales were lower at higher [NO_3_-] (Figure S32a). In the summer 2015 archaeal functionally mapped data, aerobic ammonia oxidizers and methanogens (disproportionation of methyl groups) had higher abundances at higher [NO_3_-] (Figure S26b). In the summer 2015 bacterial functionally mapped data, fermenters and photoheterotrophs had lower abundances at higher [NO_3_-] (Figure S32b).

### 1.4.2 Categorical variables (classification rf)

The most important taxa for classifying samples between the four sites in the spring 2014/2015 data were classes Subgroup 2 within phylum Acidobacteria, and Flavobacteria (Figure S33a). The most important functional mapping groups for classifying samples by sites in the spring 2014/2015 data were aerobic chemoheterotrophs, cyanobacteria, dark oxidizers of sulfur compounds (including sulfide and thiosulfate oxidation), fermenters, intracellular parasites, nitrate denitrifiers, photoheterotrophs, exoparasites, and ureolytic microbes (Figure S33b). In summer 2015 data, the archaeal phyla Bathyarchaeota, Thaumarchaeota, and Woesearchaeota (DHVEG-6; Figure S36a), together with the bacterial phylum Gracilibacteria were most important for classifying the sites (Figure S37a). In summer 2015 data sets, the functionally predicted methanogens (by disproportionation of methyl groups; Figure S36b), anoxygenic photoautotrophs, fermenters, and methanotrophs were most important in differentiating between the two sites (Figure S37b).

In the spring 2014/2015 data, the abundance changes in a single group of microbes, methanogenic Euryarchaeota, could be used to divide the samples accurately (OOB Error = 0%) by origin to Lake Hazen and Skeleton Lake; the group was absent in Lake Hazen and present in Skeleton Lake (Figure S34).

# 2 Supplementary figures and tables

## 2.1 Sediment samples


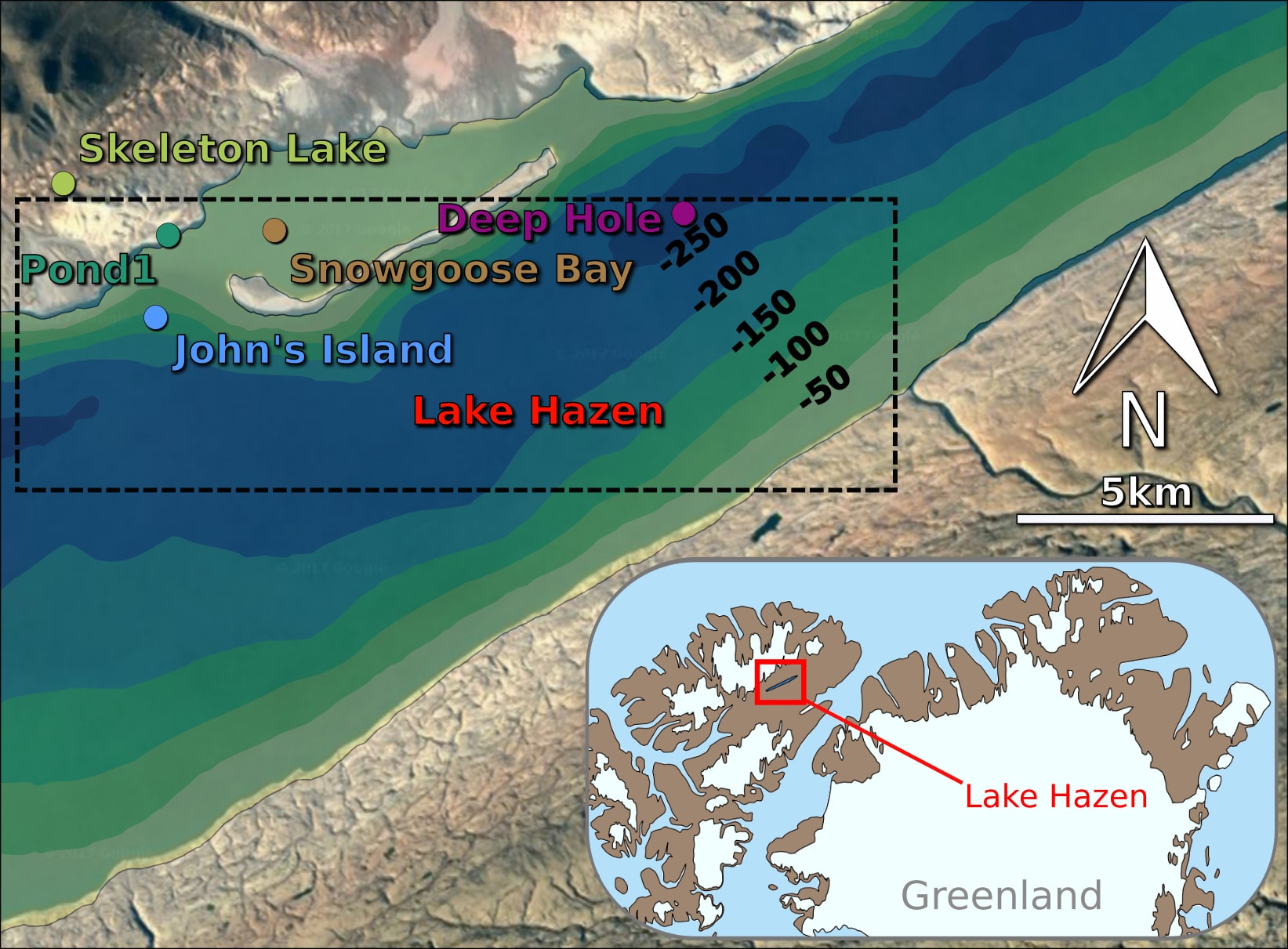
Figure S1: Map of the sampling sites and location of the sampling area. Enclosed depth map: (Köck et al., 2012) map data: Google Earth / Terrametrics (2017).


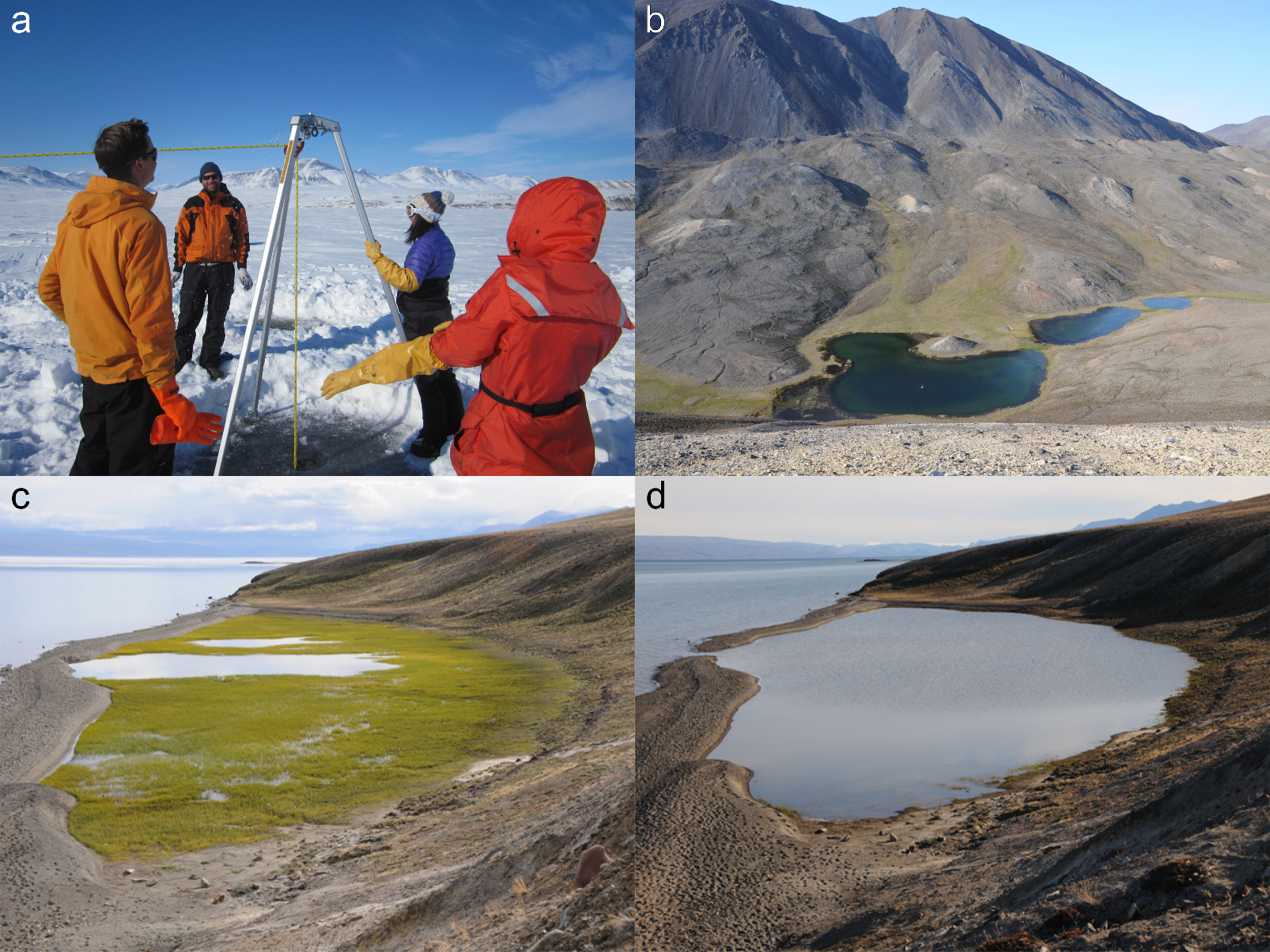
Figure S2: Photographs of the sampling area. **a**: Sampling at the Deep Hole site on Lake Hazen in spring 2015, looking NW. **b**: View over Skeleton Lake (leftmost) in summer 2015. **c**: Pond1 on July 15^th^, 2010, looking SW towards Lake Hazen (on the left), showing the development of vegetation. **d**: Pond1 on July 19^th^, 2010, showing a change in water level after formation of the hydrological connection to Lake Hazen.


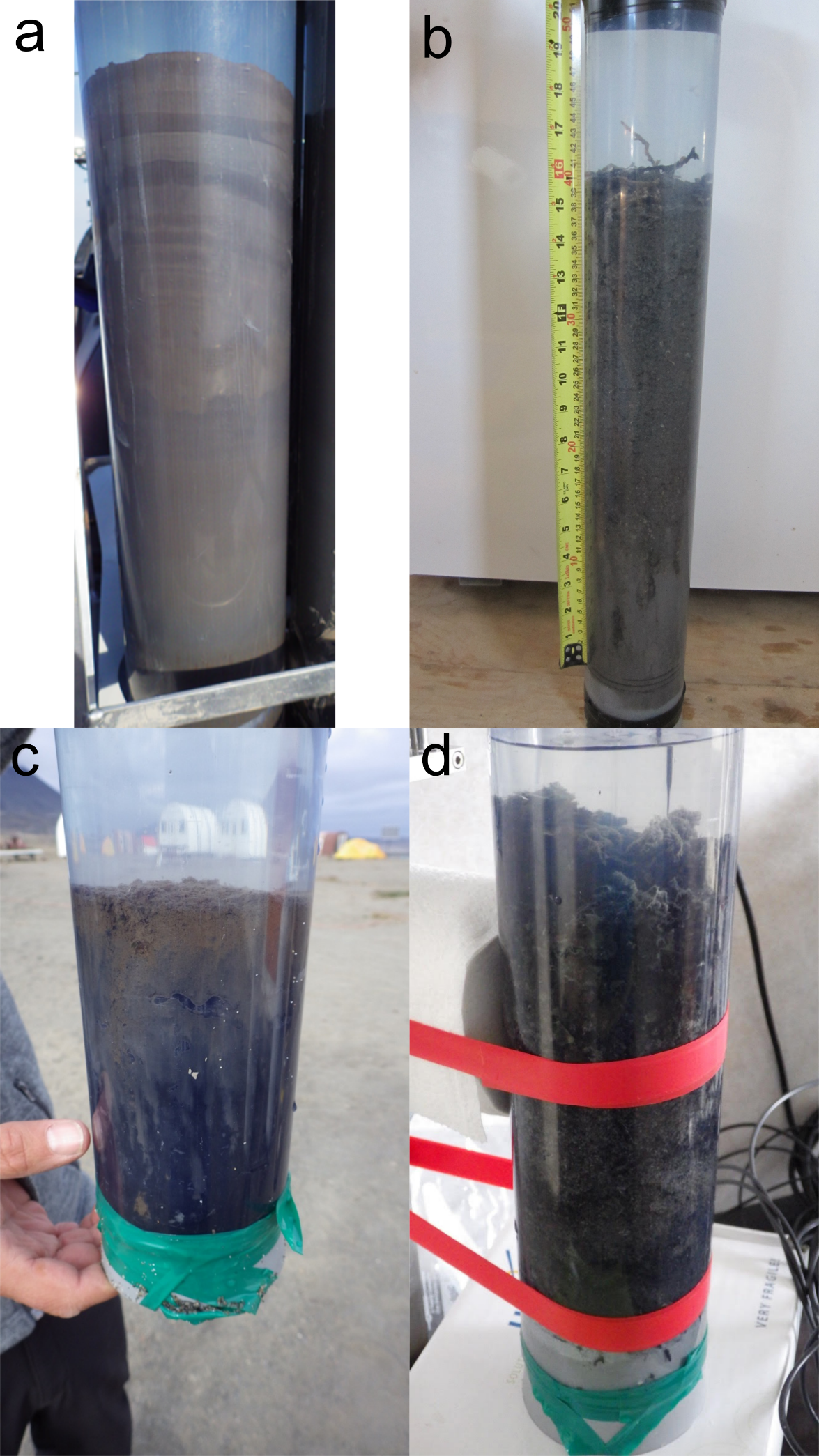
Figure S3: Photos of sediment cores from **a**: Lake Hazen Deep Hole in spring 2014, **b**: Skeleton Lake (deeper site) in spring 2015, **c**: Pond1 in summer 2015, and **d**: Skeleton Lake (shallower site) in summer 2015

Table S1: Physicochemical data for spring 2014/2015 samples. Values with higher resolution than sampling for DNA extraction were averaged over the range in question.

| Lake | Site | Year | Water depth (m) | Sediment depth (cm) | H_2_S (µM) | pH | Redox (mV) | O_2_ (mgL^-1^) |
| --- | --- | --- | --- | --- | --- | --- | --- | --- |
|  |  |  |  |  |  |  |  |  |
| Lake Hazen | Snowgoose Bay | 2014 | 44 | 0.25 | 0.37 | 7.78 | 181.02 | 9.49 |
|  |  |  |  | 0.5 | 0.66 | 7.82 | 147.86 | 6.78 |
|  |  |  |  | 0.75 | 1.01 | 7.85 | 139.69 | 5.04 |
|  |  |  |  | 1 | 1.17 | 7.87 | 133.67 | 3.69 |
|  |  | 2015 | 50 | 1 | 0 | 7.57 | 444.72 | 0.34 |
|  |  |  |  | 2 | 0.33 | 7.65 | 423.43 | 0 |
|  |  |  |  | 3 | 0 | 7.61 | 408.65 | 0 |
|  |  |  |  | 4 | 0.23 | 7.57 | 379.81 | 0 |
|  |  |  |  | 5 | 0.79 | 7.54 | 351.84 | 0 |
|  |  |  |  |  |  |  |  |  |
|  | Deep Hole | 2014 | 258 | 0.25 | 0 | 8.40 | 112.85 | 4.19 |
|  |  |  |  | 0.5 | 0 | 8.39 | 125.02 | 1.81 |
|  |  |  |  | 0.75 | 0 | 8.37 | 114.62 | 0.20 |
|  |  |  |  | 1 | 0 | 8.37 | 100.41 | 0 |
|  |  | 2015 | 261 | 1 | 0 | 7.04 | 444.35 | 0.71 |
|  |  |  |  | 2 | 0 | 7.02 | 429.24 | 0 |
|  |  |  |  | 3 | 0 | 7.01 | 416.65 | 0 |
|  |  |  |  | 4 | 0 | 7.01 | 397.13 | 0 |
|  |  |  |  | 5 | 0 | 7.02 | 383.14 | 0 |
|  |  |  |  |  |  |  |  |  |
|  | John’s Island | 2015 | 141 | 1 | 0 | 8.68 | 369.22 | 11.77 |
|  |  |  |  | 2 | 0 | 8.92 | 397.32 | 8.35 |
|  |  |  |  | 3 | 0 | 9.01 | 407.65 | 6.80 |
|  |  |  |  | 4 | 0 | 8.95 | 411.88 | 5.70 |
|  |  |  |  | 5 | 0 | 8.90 | 413.02 | 4.67 |
|  |  |  |  |  |  |  |  |  |
| Skeleton Lake |  | 2015 | 4 | 1 | 119.31 | 7.35 | 189.38 | 0 |
|  |  |  |  | 2 | 169.83 | 7.30 | 204.96 | 0 |
|  |  |  |  | 3 | 169.83 | 7.27 | 91.41 | 0 |
|  |  |  |  | 4 | 169.83 | 7.19 | 135.81 | 0 |
|  |  |  |  | 5 | 169.83 | 7.15 | 127.47 | 0 |

Table S2: Physicochemical data for summer 2015 samples. Values with higher resolution than sampling for DNA extraction were averaged over the range in question.

| Site | Water depth (m) | Sediment depth (cm) | pH | O**_2_** (mgL^-1^) | NO_3_^-^ (mgL^-1^) | Cl^-^ (mgL^-1^) | SO_4_^2-^ (mgL^-1^) |
| --- | --- | --- | --- | --- | --- | --- | --- |
|  |  |  |  |  |  |  |  |
| Skeleton Lake | 0.3 | 0.5 | 7.15 | 0 | 4.48 | 3.51 | 91.38 |
|  |  | 1 | 7.11 | 0 | 4.48 | 3.51 | 91.38 |
|  |  | 1.5 | 7.08 | 0 | 4.66 | 3.22 | 84.34 |
|  |  | 2 | 7.04 | 0 | 4.66 | 3.22 | 84.34 |
|  |  | 2.5 | 6.99 | 0 | 4.65 | 3.07 | 86.11 |
|  |  | 3 | 6.96 | 0 | 4.65 | 3.07 | 86.11 |
|  |  | 3.5 | 6.92 | 0 | 4.02 | 3.19 | 87.55 |
|  |  | 4 | 6.86 | 0 | 4.02 | 3.19 | 87.55 |
|  |  | 4.5 | 6.82 | 0 | 4.19 | 2.72 | 121.51 |
|  |  | 5 | 6.79 | 0 | 4.19 | 2.72 | 121.51 |
|  |  | 5.5 | 6.77 | 0 | 4.34 | 3.12 | 104.62 |
|  |  | 6 | 6.76 | 0 | 4.34 | 3.12 | 104.62 |
|  |  |  |  |  |  |  |  |
| Pond1 | 1.5 | 0.5 | 8.04 | 0.27 | 5.49 | 4.38 | 70.03 |
|  |  | 1 | 7.4 | 0 | 5.49 | 4.38 | 70.03 |
|  |  | 1.5 | 7.2 | 0 | 5.26 | 2.07 | 87.14 |
|  |  | 2 | 7.005 | 0 | 5.26 | 2.07 | 87.14 |
|  |  | 2.5 | 6.925 | 0 | 5.48 | 2.67 | 91.83 |
|  |  | 3 | 6.855 | 0 | 5.48 | 2.67 | 91.83 |
|  |  | 3.5 | 6.81 | 0 | 5.27 | 2.59 | 71.69 |
|  |  | 4 | 6.79 | 0 | 5.27 | 2.59 | 71.69 |

## 2.2 Data analysis quality control


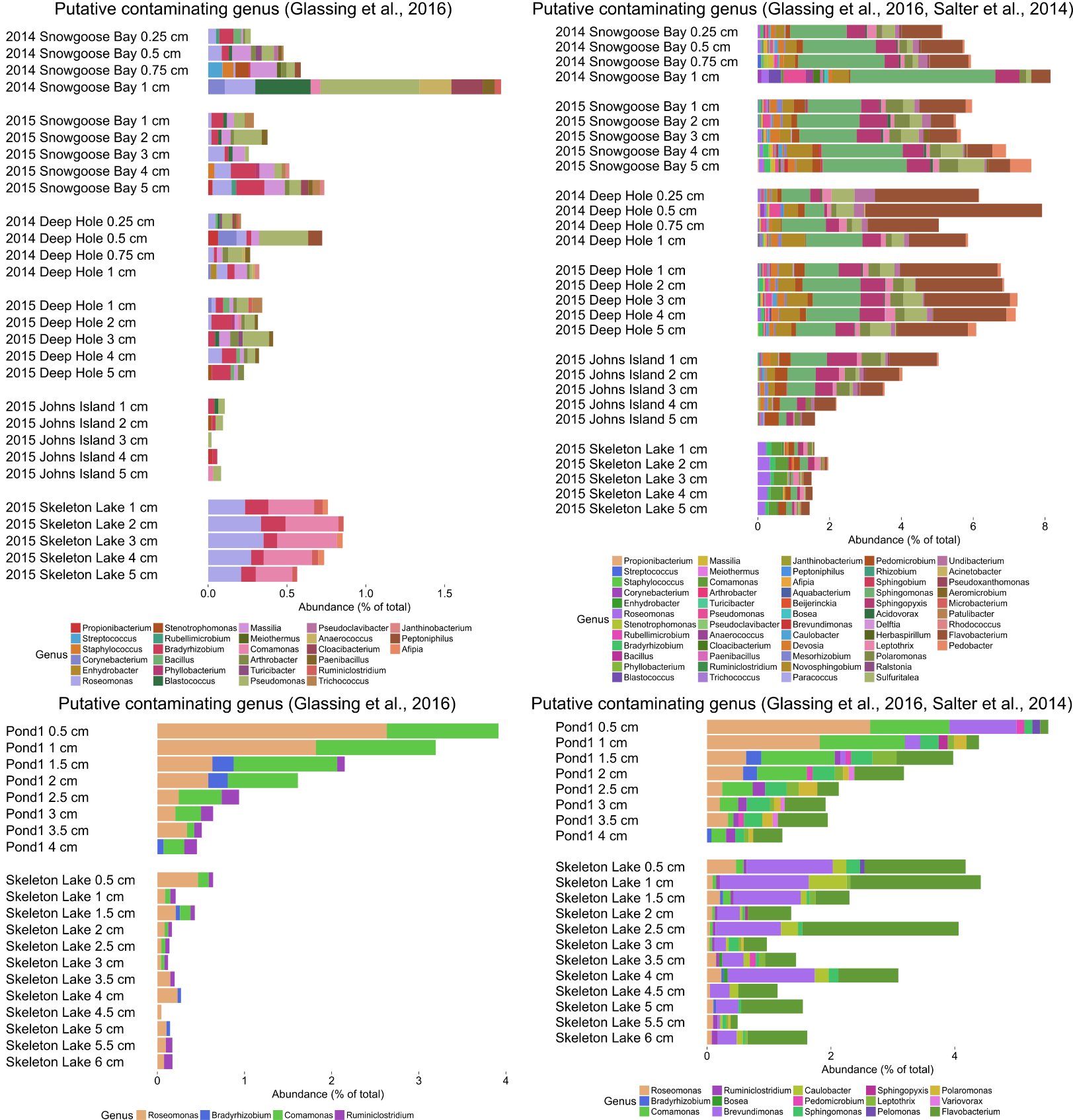


Figure S4: Per sample abundance of putative contaminant genera in spring 2014/2015 samples and summer 2015 samples identified on the left panel in the MOBIO PowerSoil kit (Glassing et al., 2016) and on the right panel in other kits and reagents (Salter et al., 2014). Only bacterial data is shown for summer 2015 since no putative contaminant archaea have been identified.


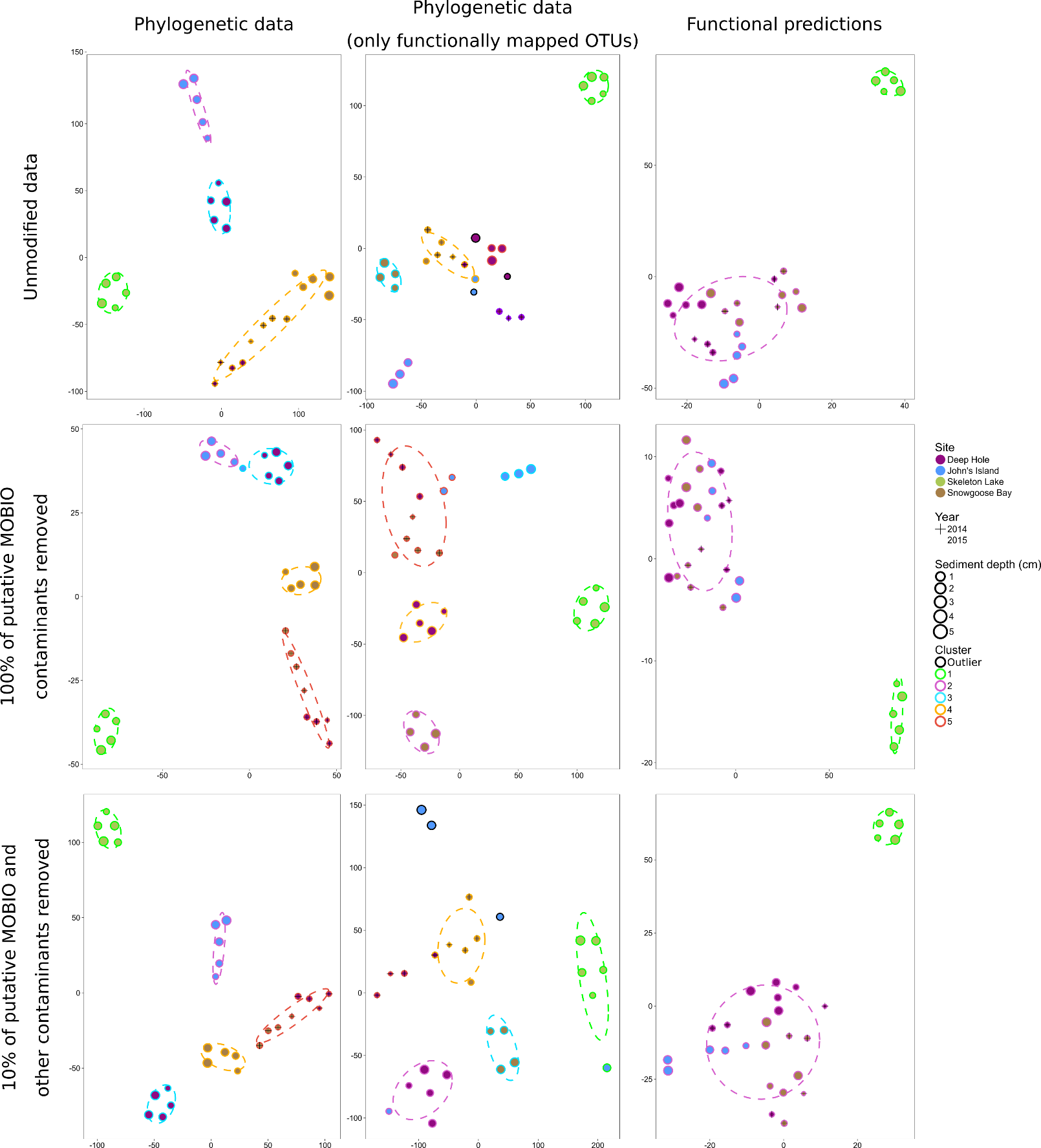


Figure S5: Effects of removing putative contaminant genera on the tSNE clustering patterns in spring 2014/2015 data. Unmodified data compared to analyses where 100% of genera identified in the MOBIO PowerSoil kit (Glassing et al., 2016) and 10% of both MOBIO and other kit contaminants (Salter et al., 2014) were removed.


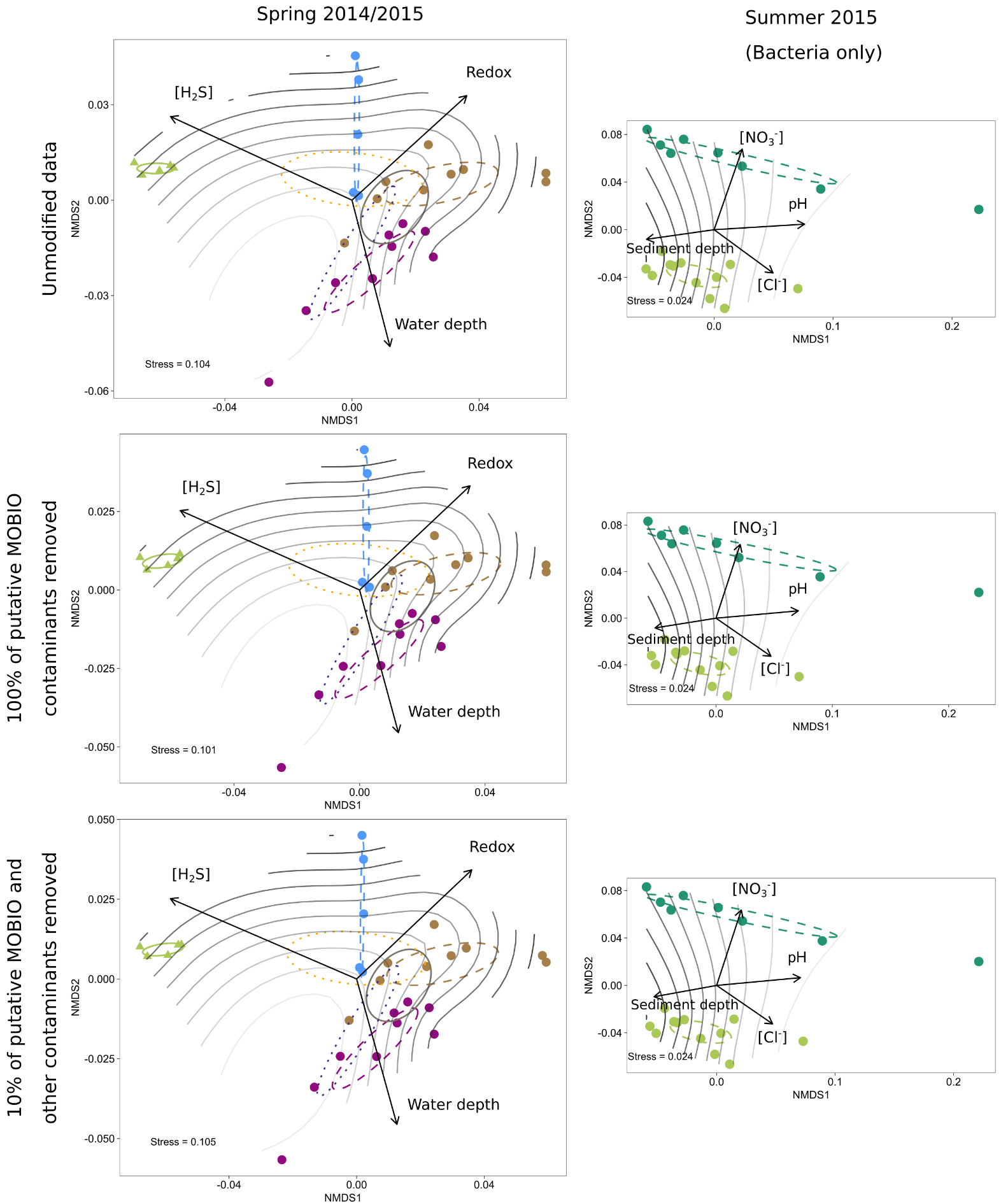


Figure S6: Effects of removing putative contaminant genera on the NMDS ordination patterns in spring 2014/2015 and summer 2015 bacterial data. Unmodified data compared to analyses where 100% of genera identified in the MOBIO PowerSoil kit (Glassing et al., 2016) and 10% of both MOBIO and other kit contaminants (Salter et al., 2014) were removed. Only bacterial data is shown for summer 2015 since no putative contaminant archaea have been identified.

Table S3: Number of reads and OTUs after each handling step.

|  | Data set: | | |
| --- | --- | --- | --- |
|  | spring 2014/2015 | summer 2015 archaeal | summer 2015 bacterial |
|  |  |  |  |
| Raw reads | 5,395,074 | 4,894,213 | 4,753,402 |
| Reads after pairing | 5,357,889 | 4,723,603 | 4,333,772 |
| Reads after sample pruning & QC | 560,323 | 390,236 | 148,030 |
| Reads after chimera picking | 482,308 | 361,960 | 132,832 |
| Unique (dereplicated) reads | 240,833 | 169,365 | 87,590 |
| Total clusters (OTUs) | 75,600 | 36,177 | 39,359 |
| Non-singleton OTUs | 16,933 | 4,865 | 5,415 |
| OTUs after sample pruning & taxonomic QC | 15,176 | 1,067 | 5,240 |
| OTUs with > 0.1 ‰ overall abundance | 2,655 | 1,067 | 2,928 |
|  |  |  |  |
| Functionally mapped OTUs | 3,772 (25%) | 153 (14%) | 1,238 (24%) |
| Unique functional mapping groups | 48 | 10 | 26 |


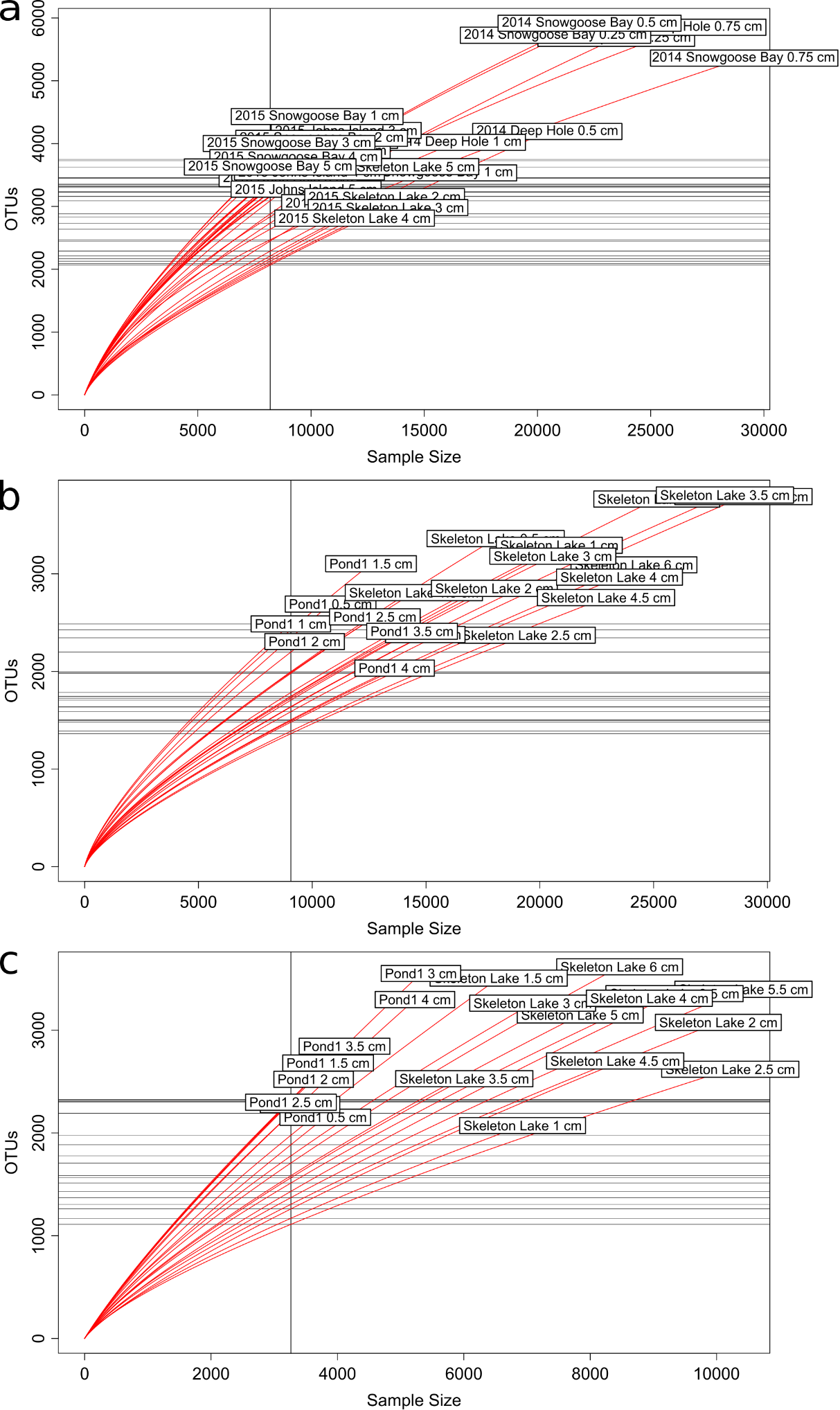
Figure S7: Rarefaction curves of the data sets showing number of OTUs as a function of rarefied read counts on non-normalized data. Vertical line shows the smallest number of reads in a sample in the data set in question, while horizontal lines show number of OTUs retained for samples at this rarefaction depth. **a**: spring 2014/2015 with universal primers, **b**: summer 2015 with archaeal primers, **c**: summer 2015 with bacterial primers.

##
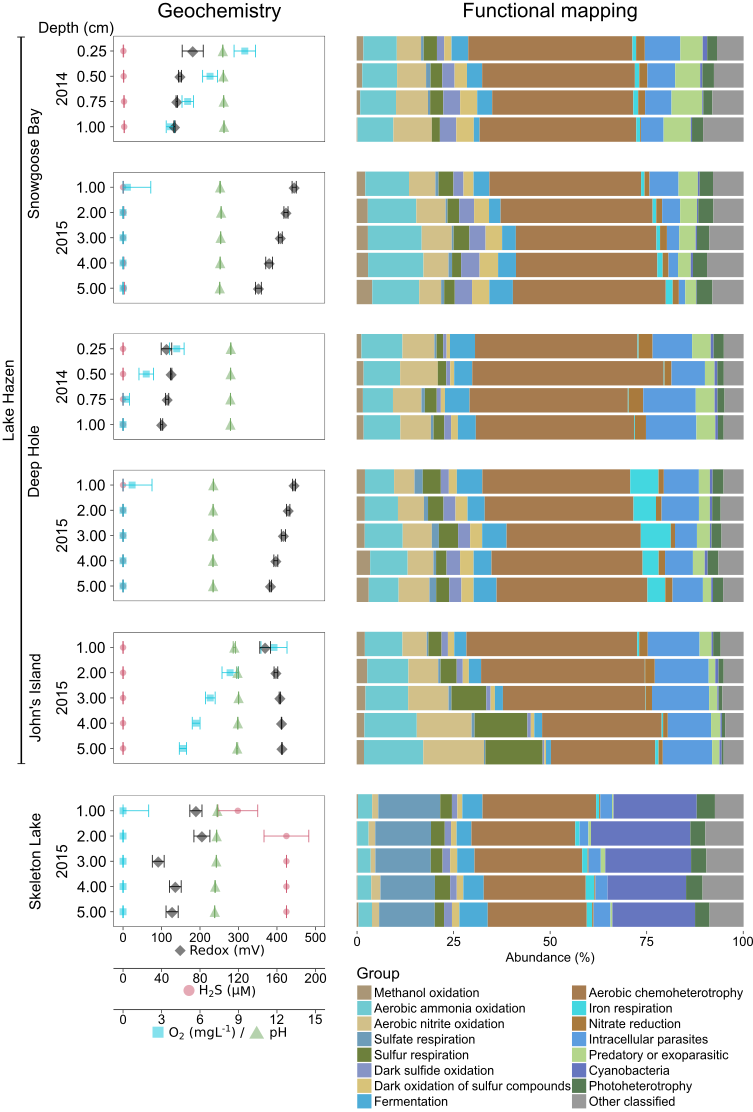
2.3 Functional mapping

Figure S8: Geochemical variability, and functional mapping group composition of the spring 2014/2015 samples using universal primers. Groups with less than 1% overall abundance in the data set are merged as “Other classified”.


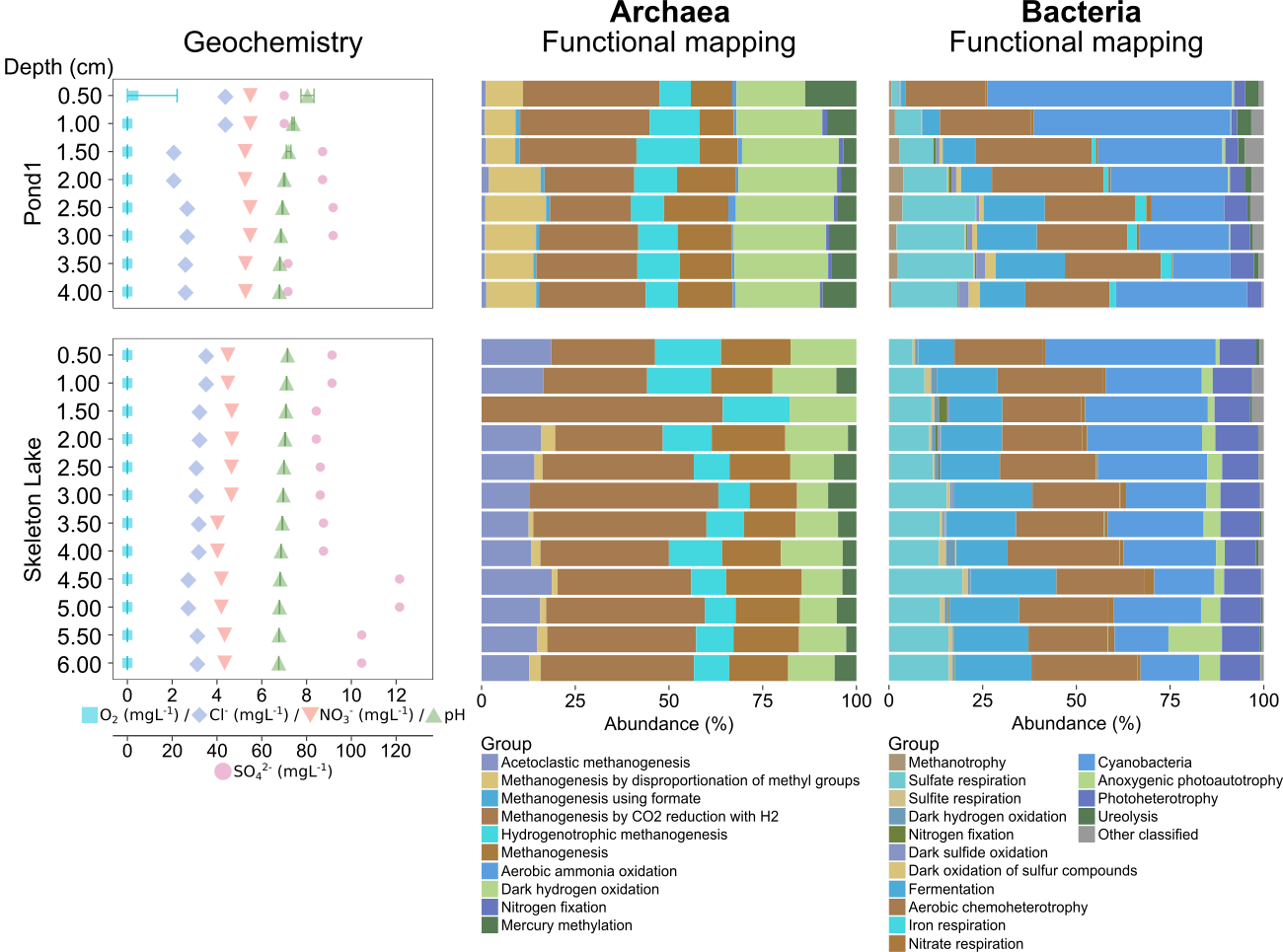


Figure S9: Geochemical variability, and functional mapping group composition of the summer 2015 samples using archaeal and bacterial primers. Groups with less than 1% overall abundance in each data set are merged as “Other classified”.

##
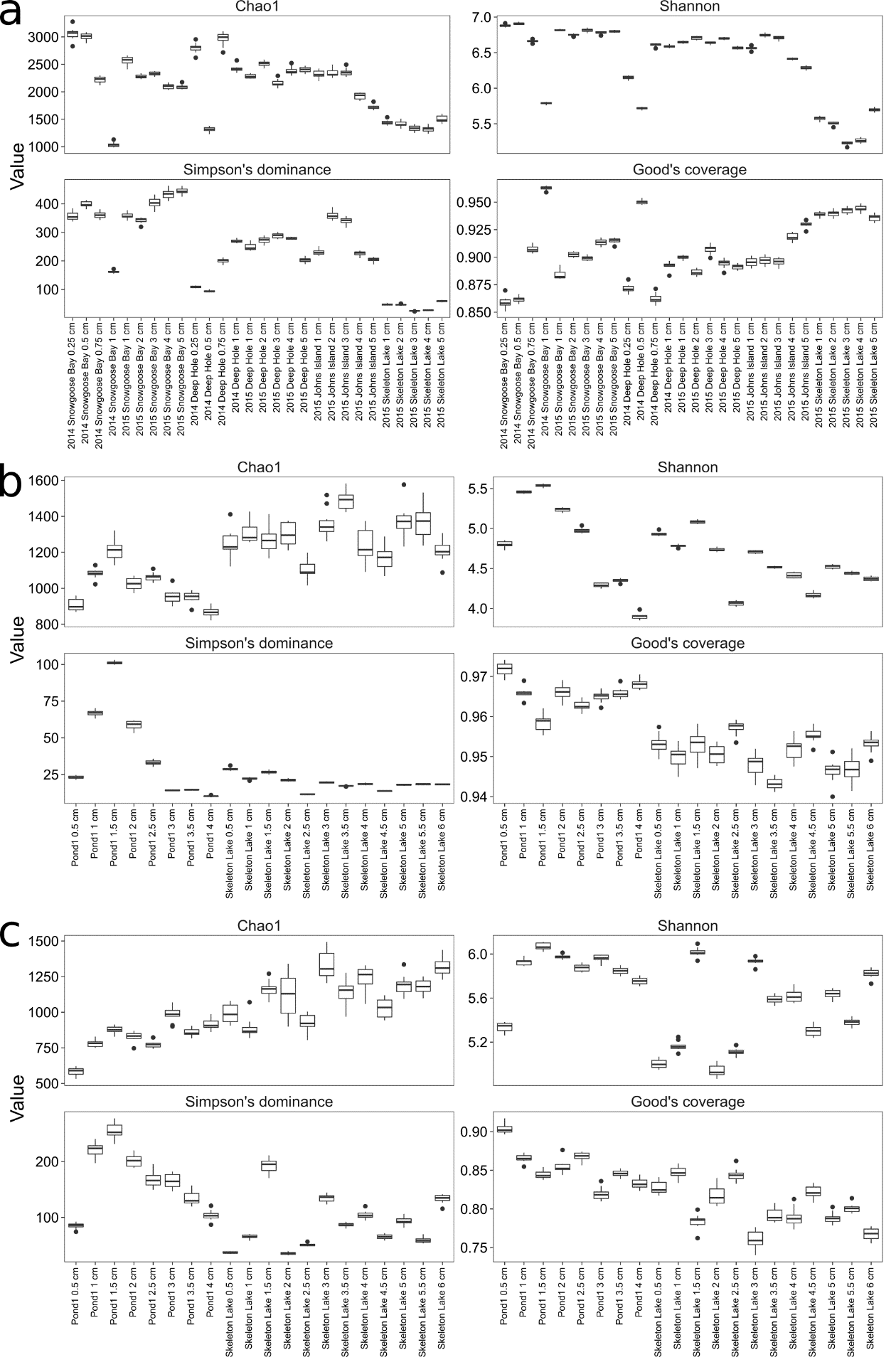
2.4 Alpha- and beta-diversity

Figure S10: Richness and diversity of the communities summarized by Chao1, Shannon, and Simpson’s Dominance (InvSimpson) indices, with Good’s Coverage. **a**: spring 2014/2015 with universal primers,  **b**: summer 2015 with archaeal primers, **c**: summer 2015 with bacterial primers.


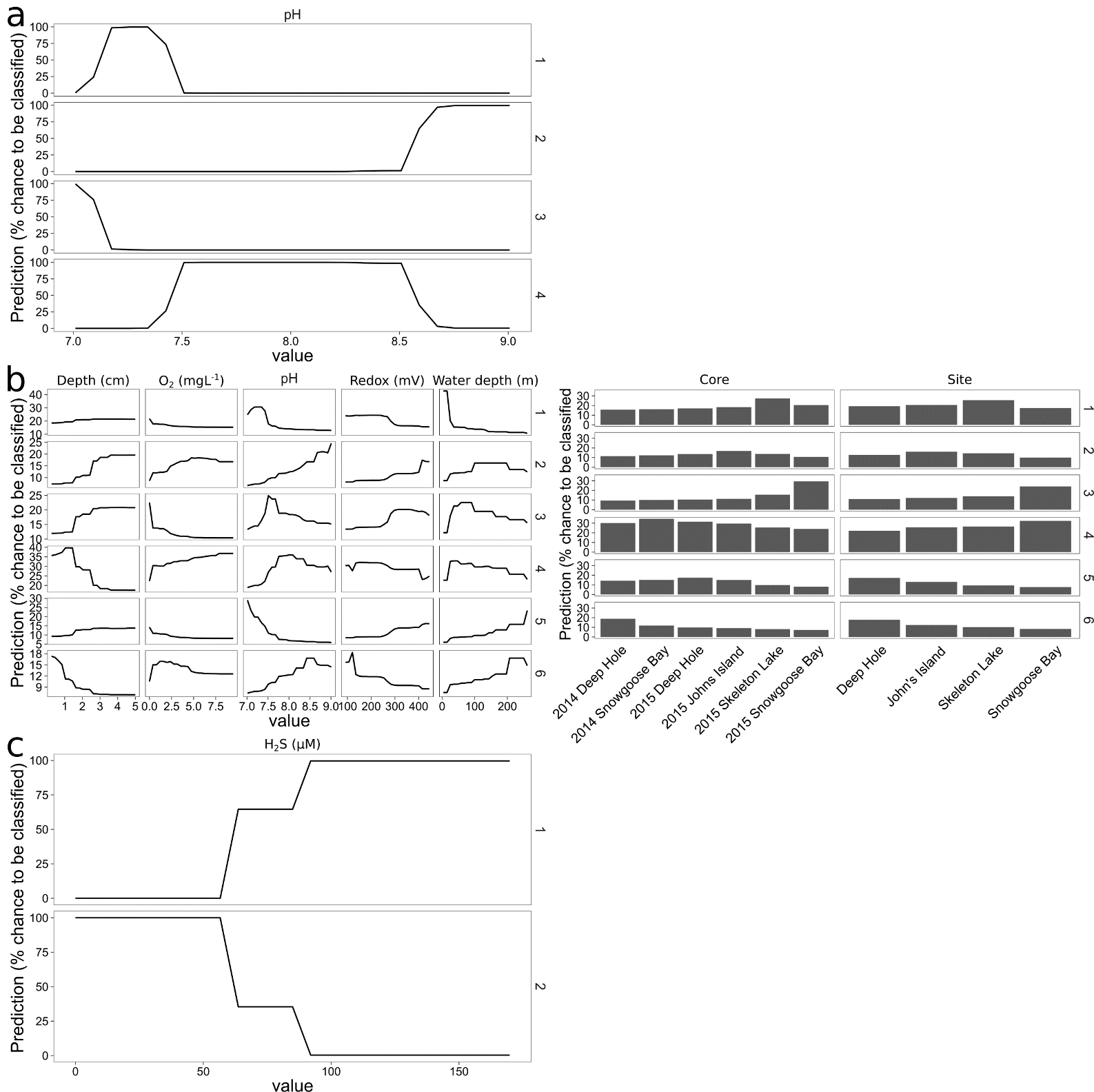


Figure S11: Partial dependence of random forest model prediction of cluster group (after recursive feature elimination) from spring 2014/2015 data set with universal primers. **a**: Data set only including OTUs with
> 0.1 ‰ overall abundance, with DPCoA distance matrix. **b**: Data set only including OTUs that were matched to a function through FAPROTAX, with DPCoA distance matrix. **c**: Functionally mapped data, with Bray-Curtis distance matrix.


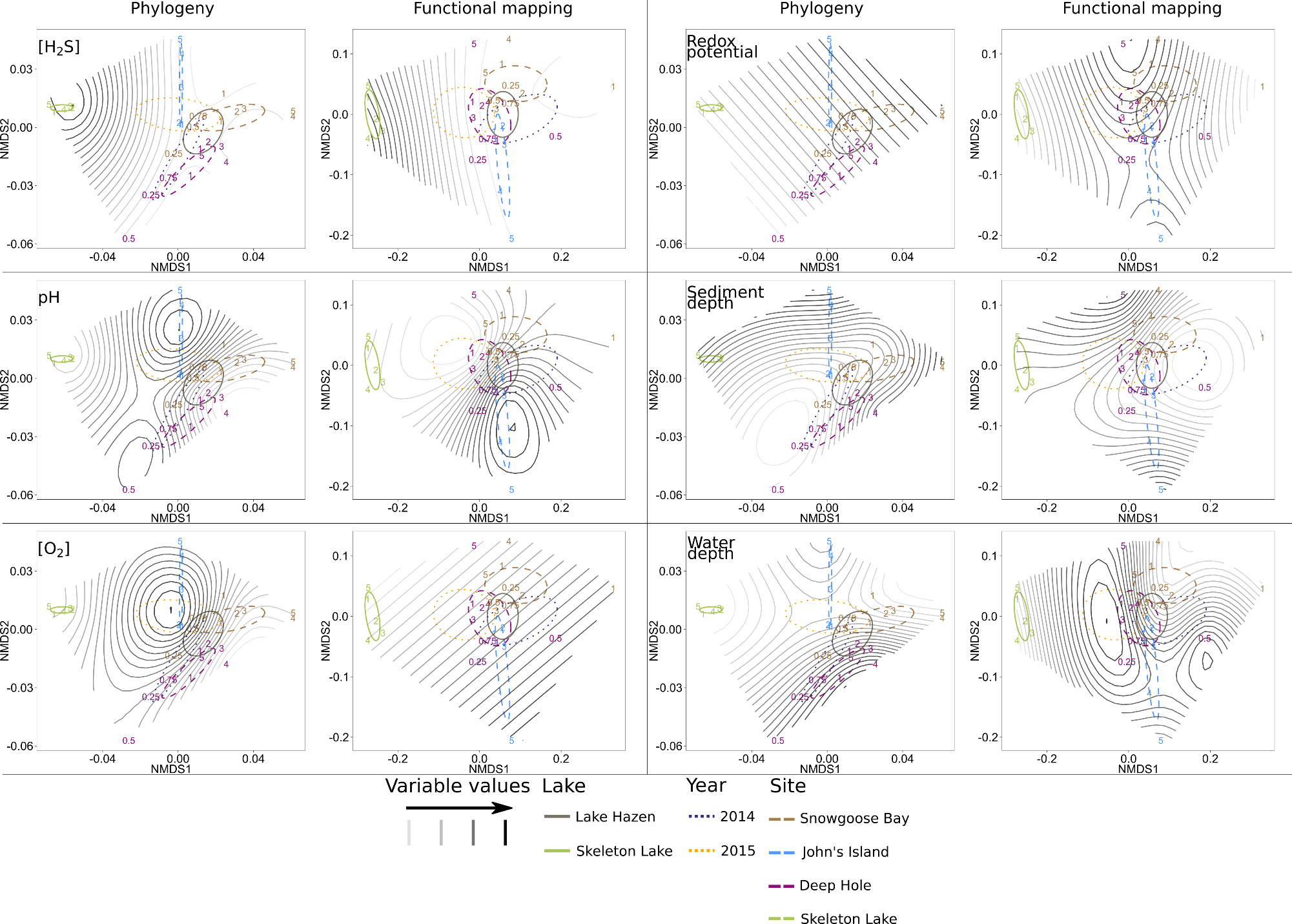

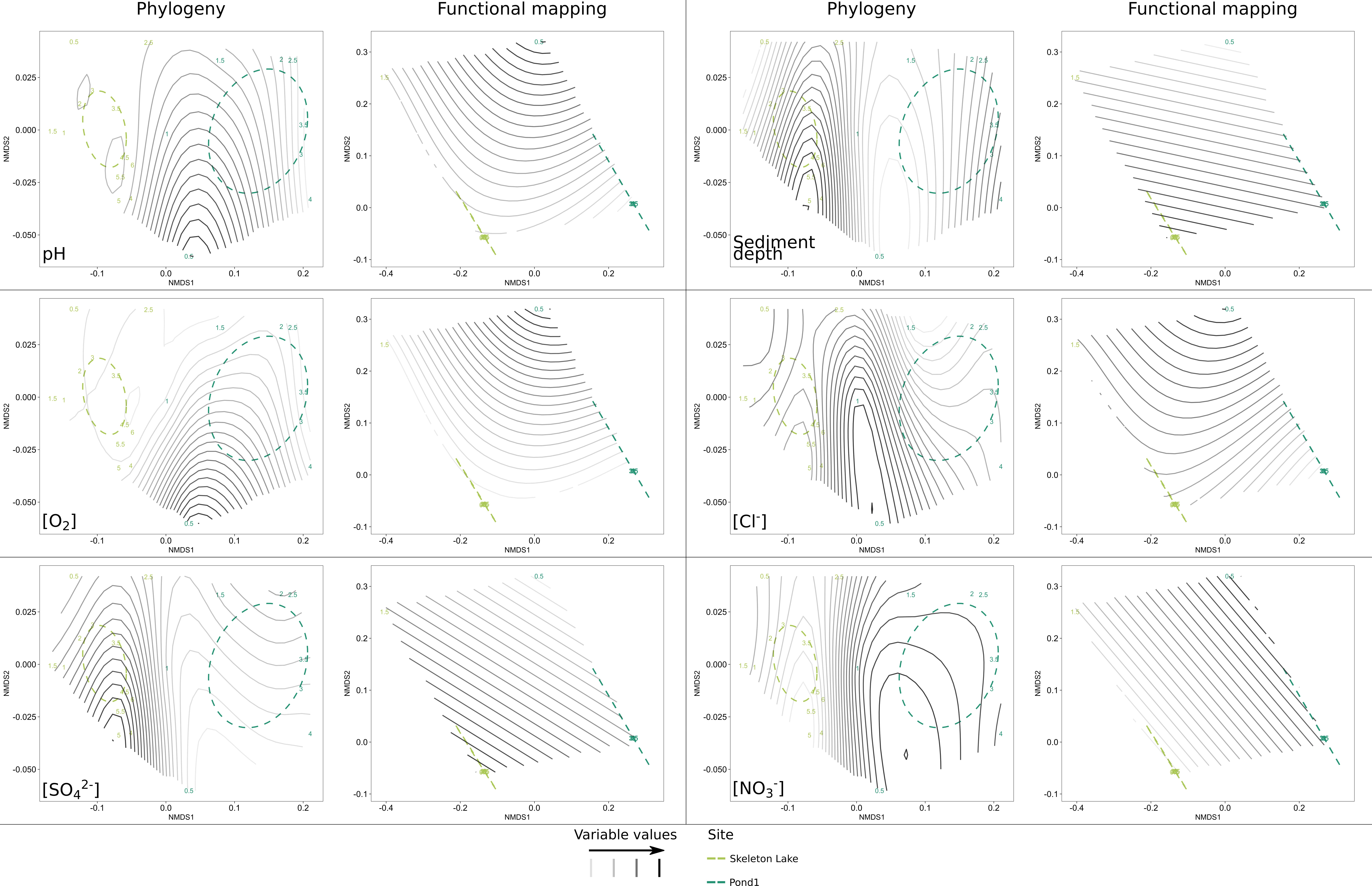
Figure S12: NMDS ordinations of the spring 2014/2015 data. Phylogenetic distances of samples through DPCoA (“Phylogeny” columns) and Bray-Curtis dissimilarity of the functional mappings (“Functional mapping” columns), with surface fits of all measured physicochemical variables, as indicated on the phylogeny column, on rows. 95%-confidence interval for centroids of sample categories (lake, year, and site) are shown with ellipses and lower extent of sampling depth in cm is indicated as the marker of each data point.


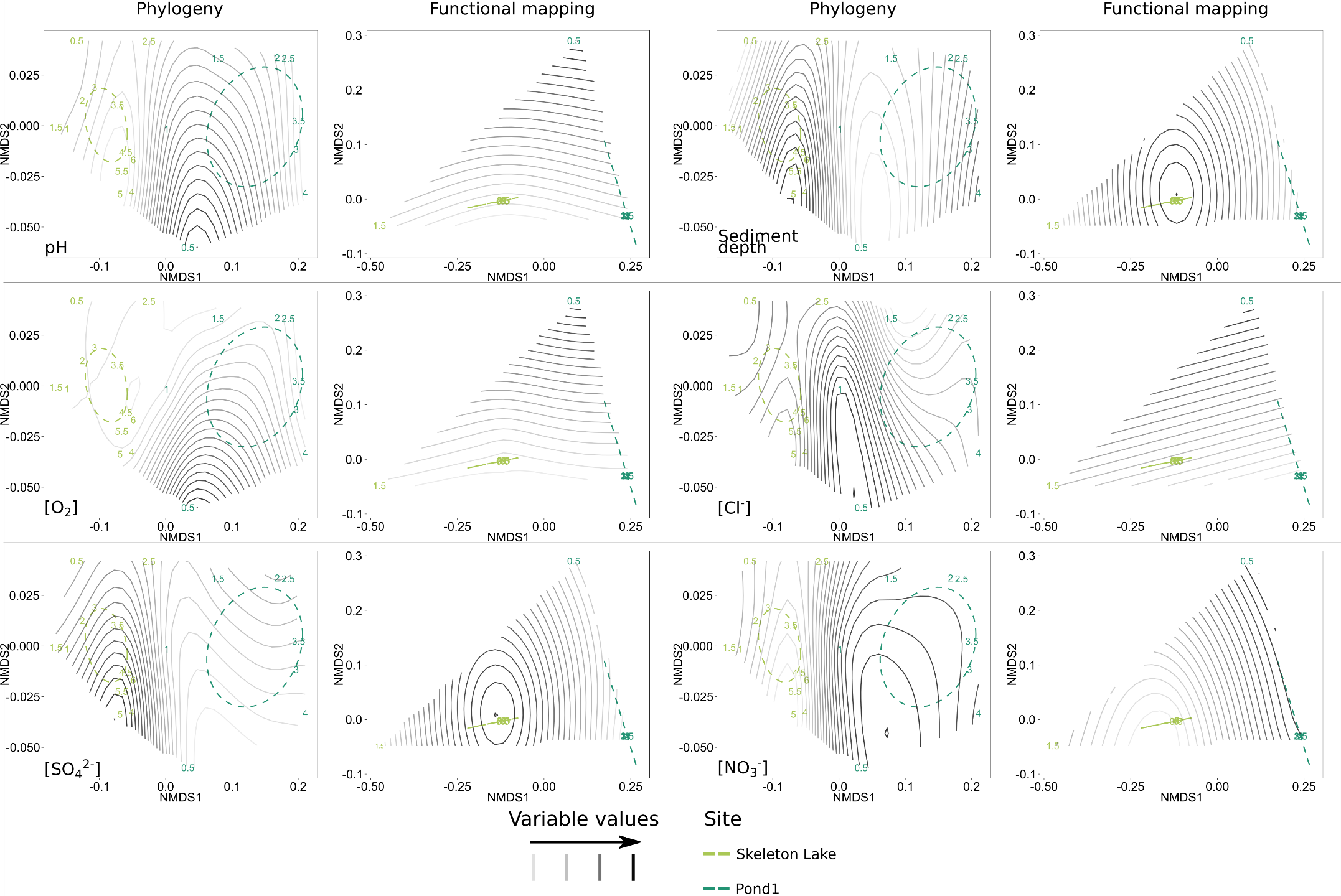
Figure S13: NMDS ordinations of the summer 2015 archaeal data. Phylogenetic distances of samples through DPCoA (“Phylogeny” columns) and Bray-Curtis dissimilarity of the functional mappings (“Functional mapping” columns), with surface fits of all measured physicochemical variables, as indicated on the phylogeny column, on rows. 95%-confidence interval for centroids of sampled sites are shown with ellipses and lower extent of sampling depth in cm is indicated as the marker of each data point.


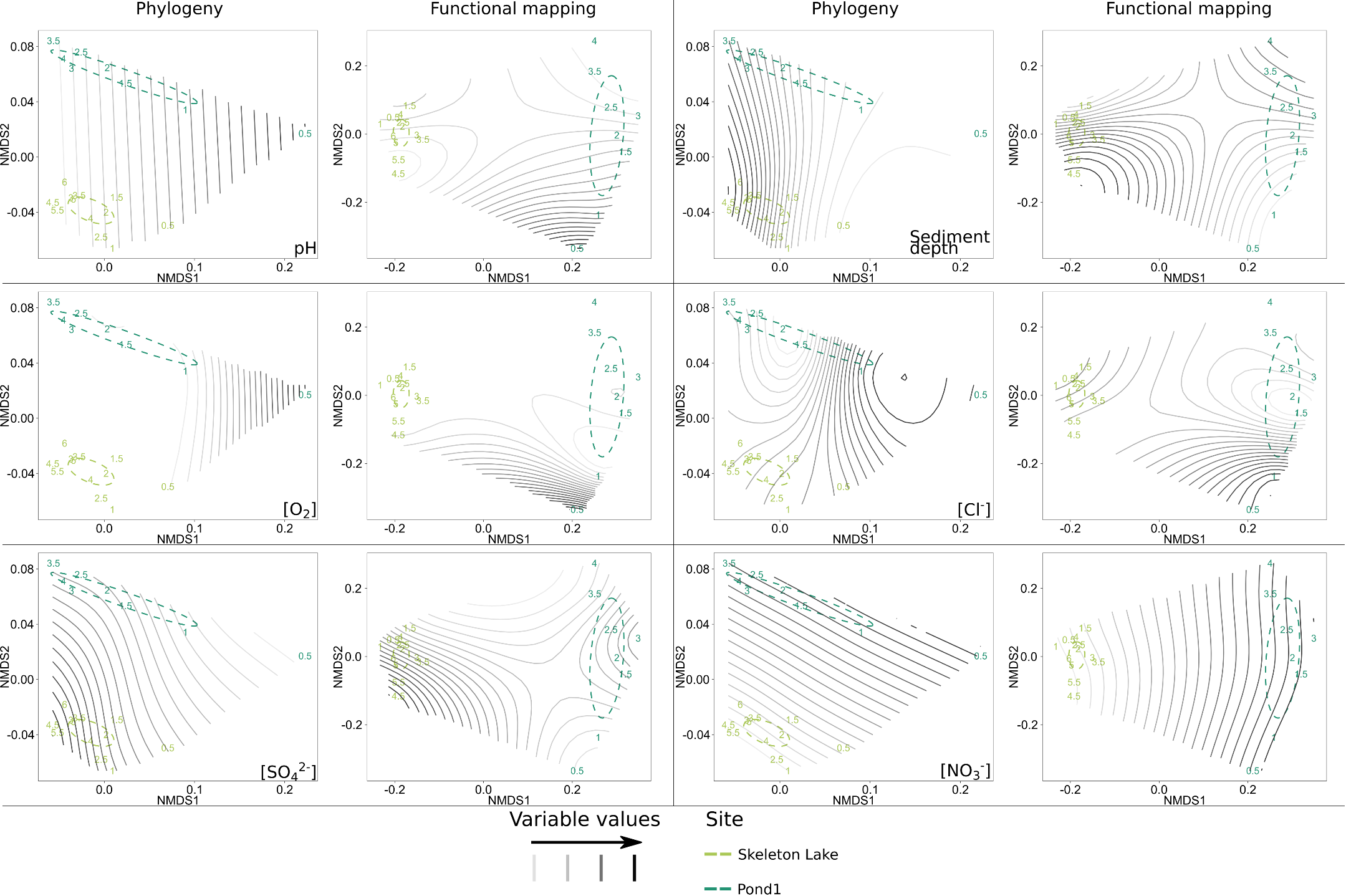


Figure S14: NMDS ordinations of the summer 2015 bacterial data. Phylogenetic distances of samples through DPCoA (“Phylogeny” columns) and Bray-Curtis dissimilarity of the functional mappings (“Functional mapping” columns), with surface fits of all measured physicochemical variables, as indicated on the phylogeny column, on rows. 95%-confidence interval for centroids of sampled sites are shown with ellipses and lower extent of sampling depth in cm is indicated as the marker of each data point.

## 2.5 Partial dependence plots of gradient analysis random forests

### 2.5.1 Continuous variables: spring 2014/2015


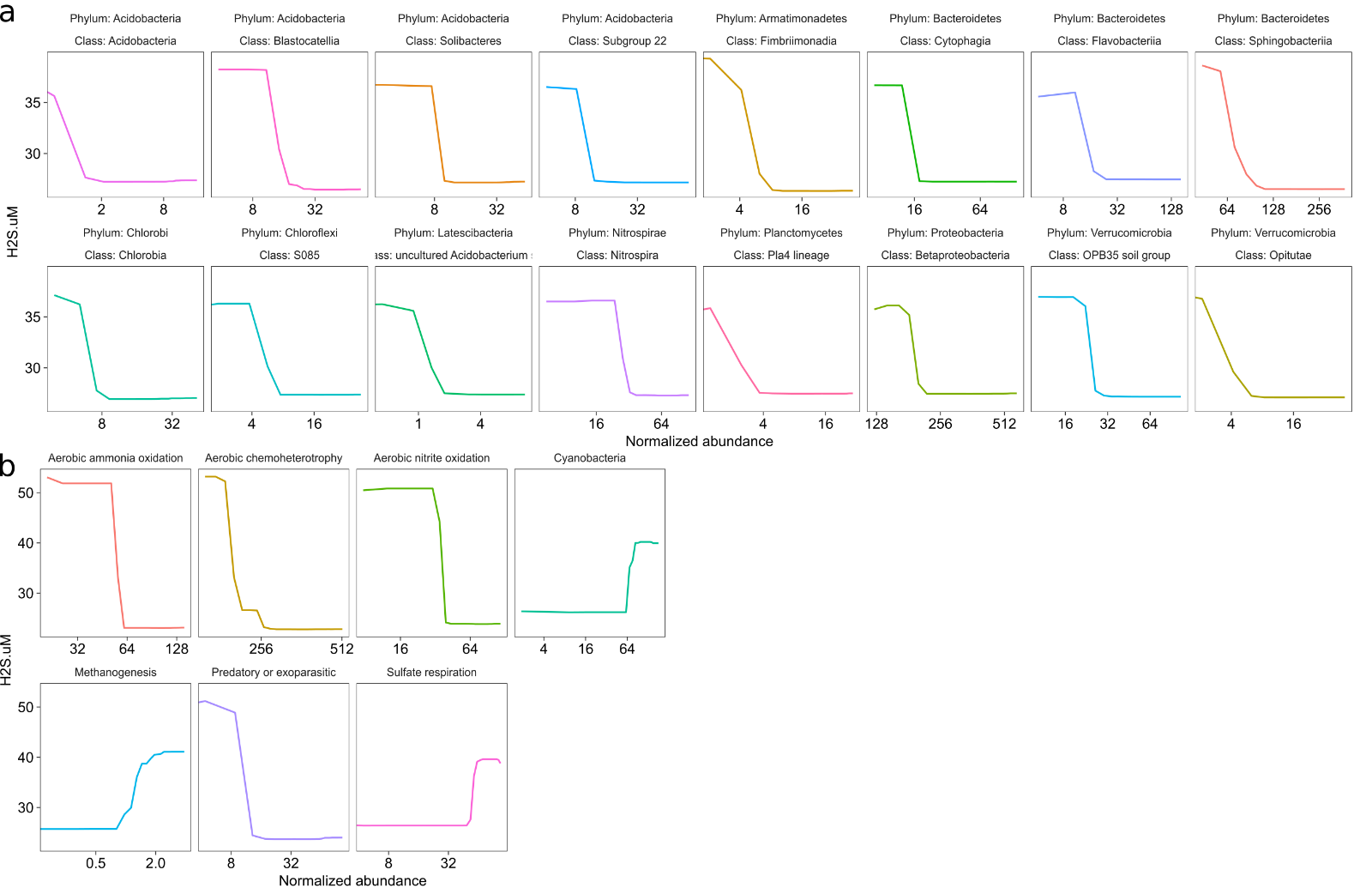


Figure S15: Partial dependence of random forest model prediction of [H_2_S] from spring 2014/2015 data set with universal primers. **a**: Phylogenetic data **b**: Functionally mapped data.


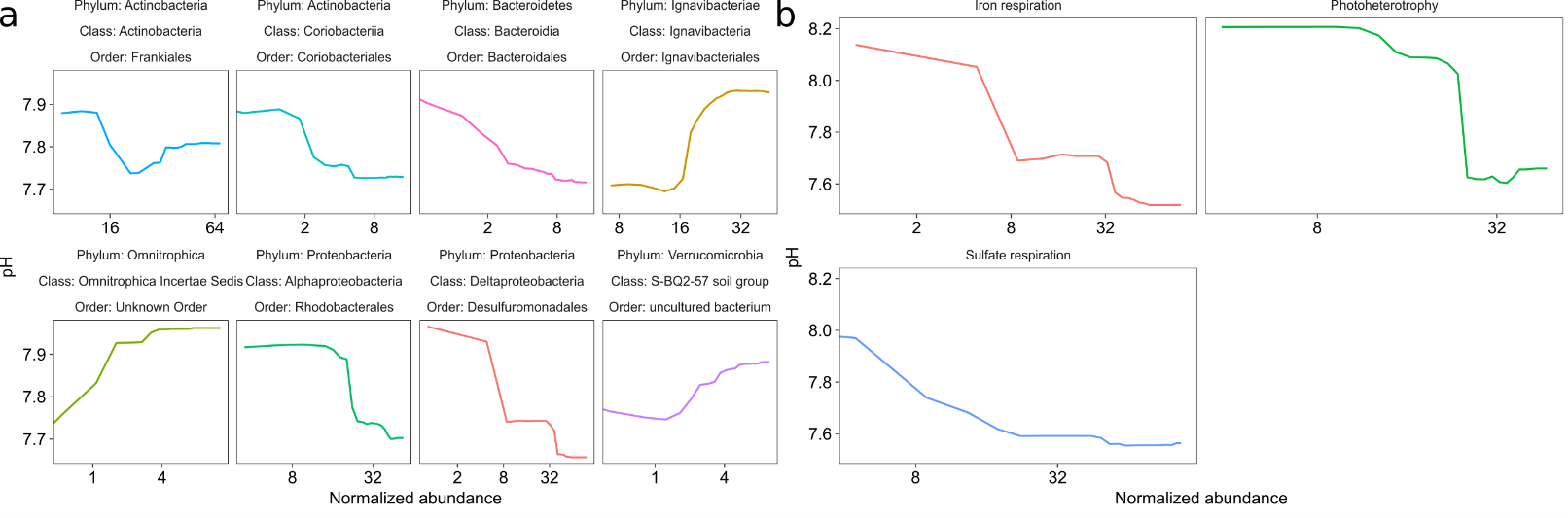


Figure S16: Partial dependence of random forest model prediction of pH from spring 2014/2015 data set with universal primers. **a**: Phylogenetic data **b**: Functionally mapped data.


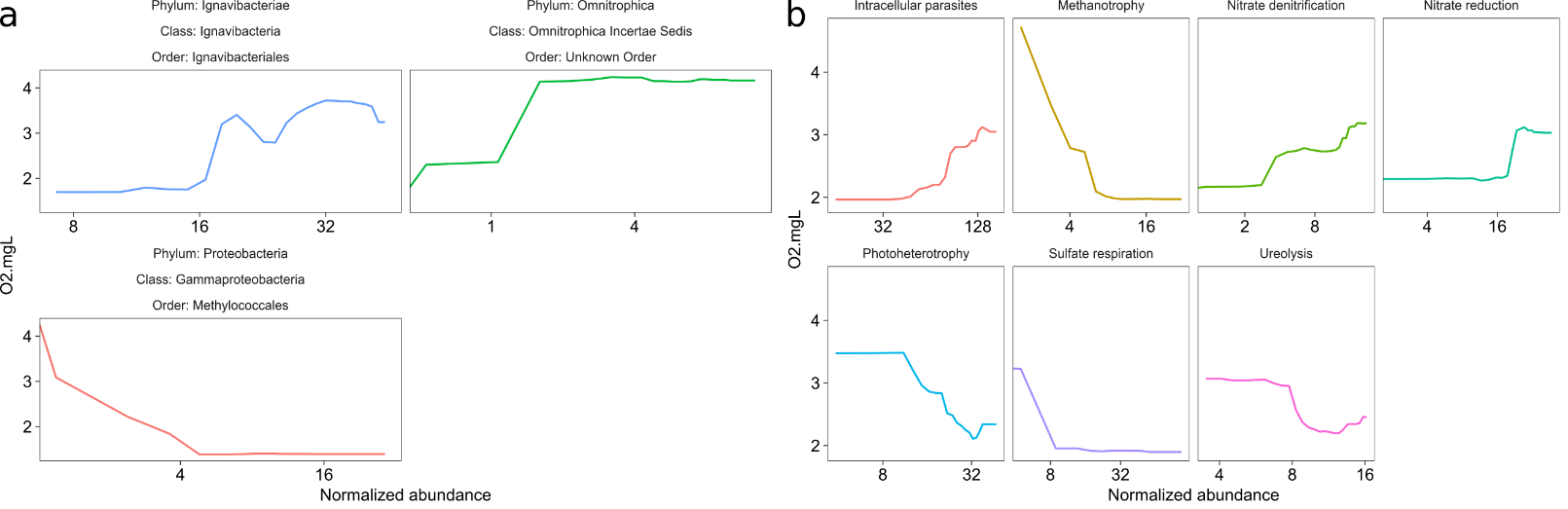


Figure S17: Partial dependence of random forest model prediction of [O2] from spring 2014/2015 data set with universal primers. **a**: Phylogenetic data **b**: Functionally mapped data.


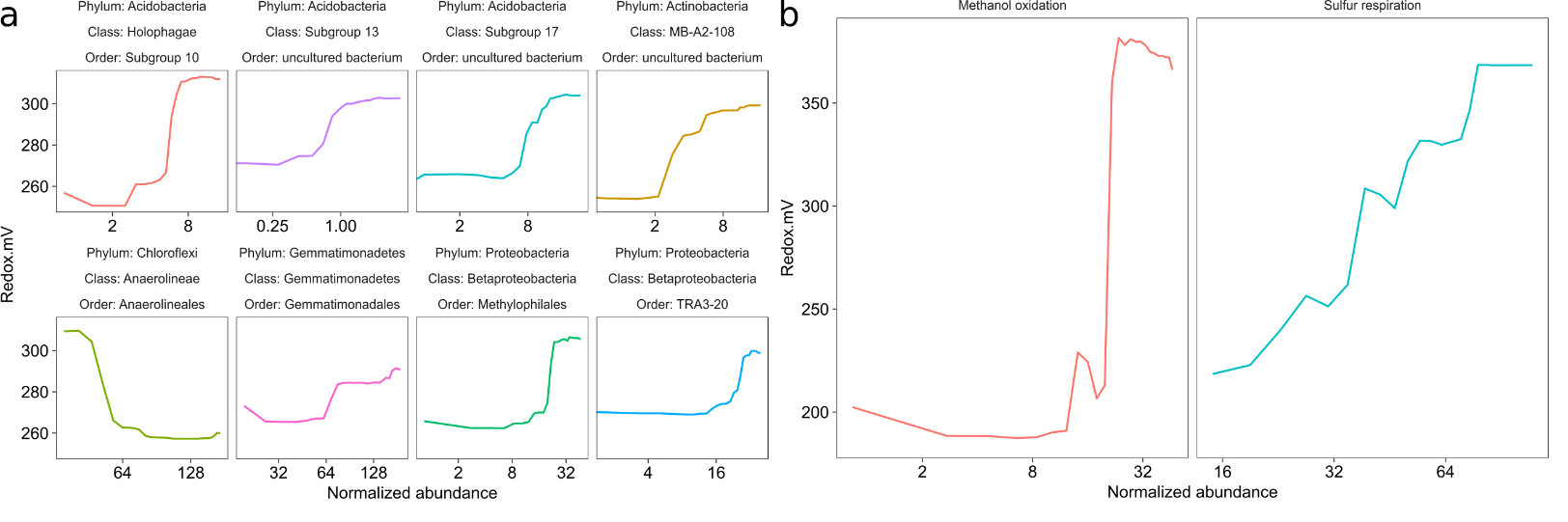


Figure S18: Partial dependence of random forest model prediction of redox potential from spring 2014/2015 data set with universal primers. **a**: Phylogenetic data **b**: Functionally mapped data.


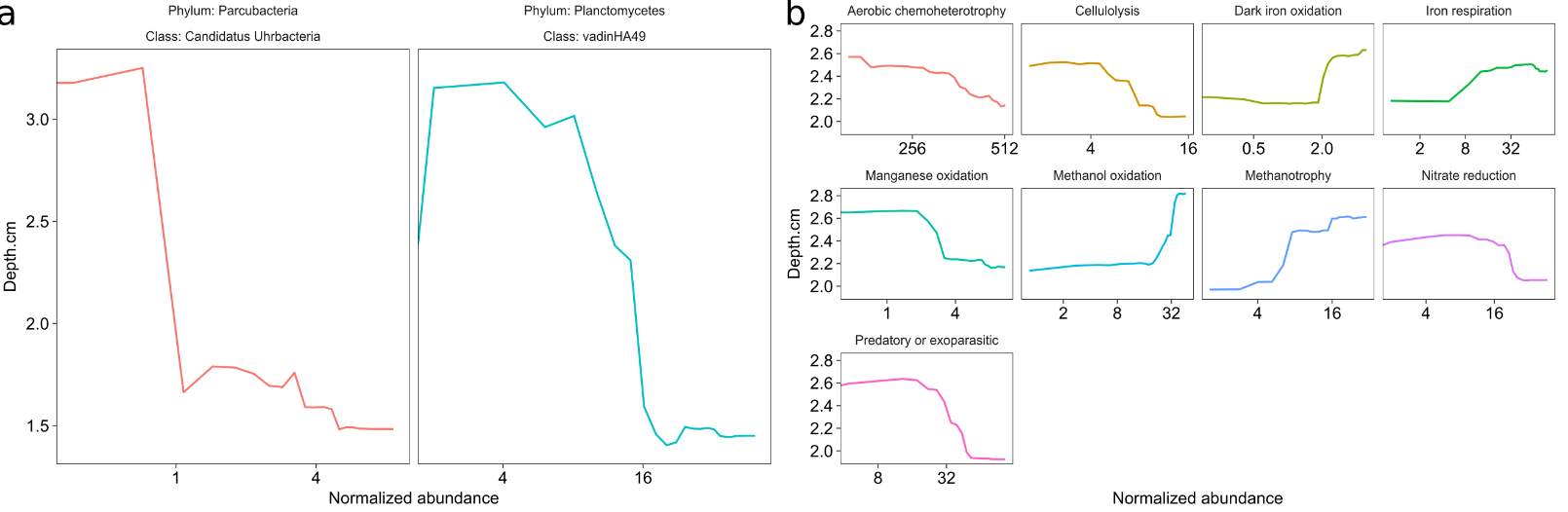


Figure S19: Partial dependence of random forest model prediction of sediment depth from spring 2014/2015 data set with universal primers. **a**: Phylogenetic data **b**: Functionally mapped data.


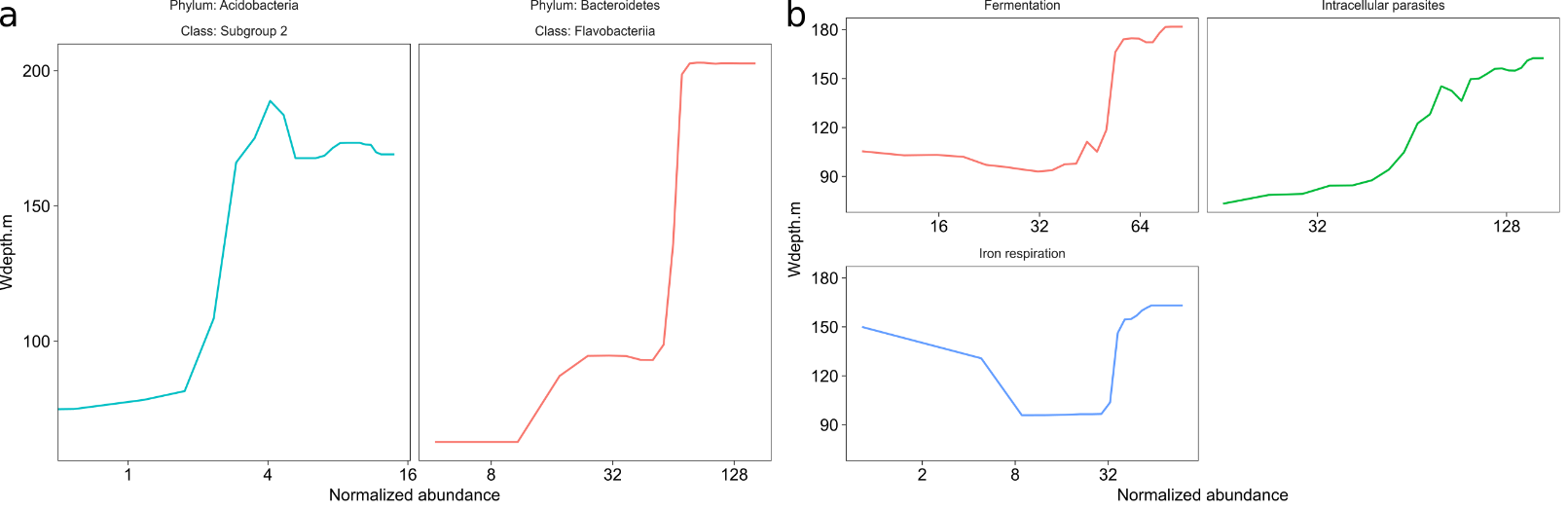


Figure S20: Partial dependence of random forest model prediction of water depth from spring 2014/2015 data set with universal primers. **a**: Phylogenetic data **b**: Functionally mapped data.

### 2.5.2 Continuous variables: summer 2015


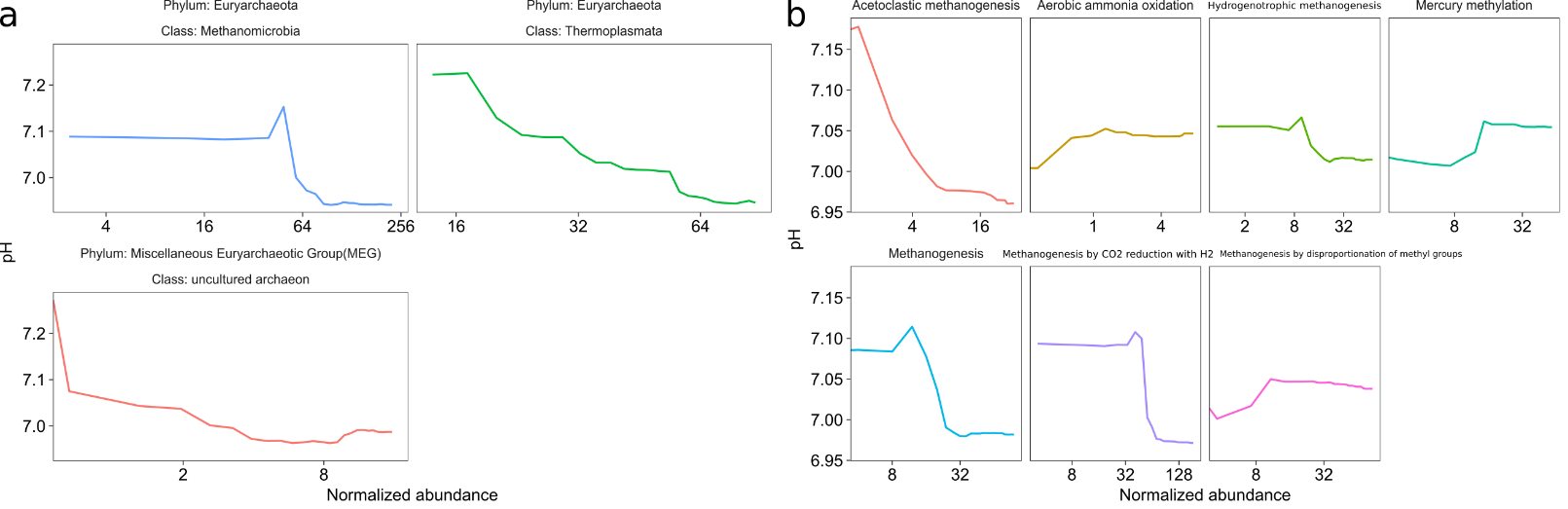


Figure S21: Partial dependence of random forest model prediction of pH from summer 2015 data set with archaeal primers. **a**: Phylogenetic data **b**: Functionally mapped data.


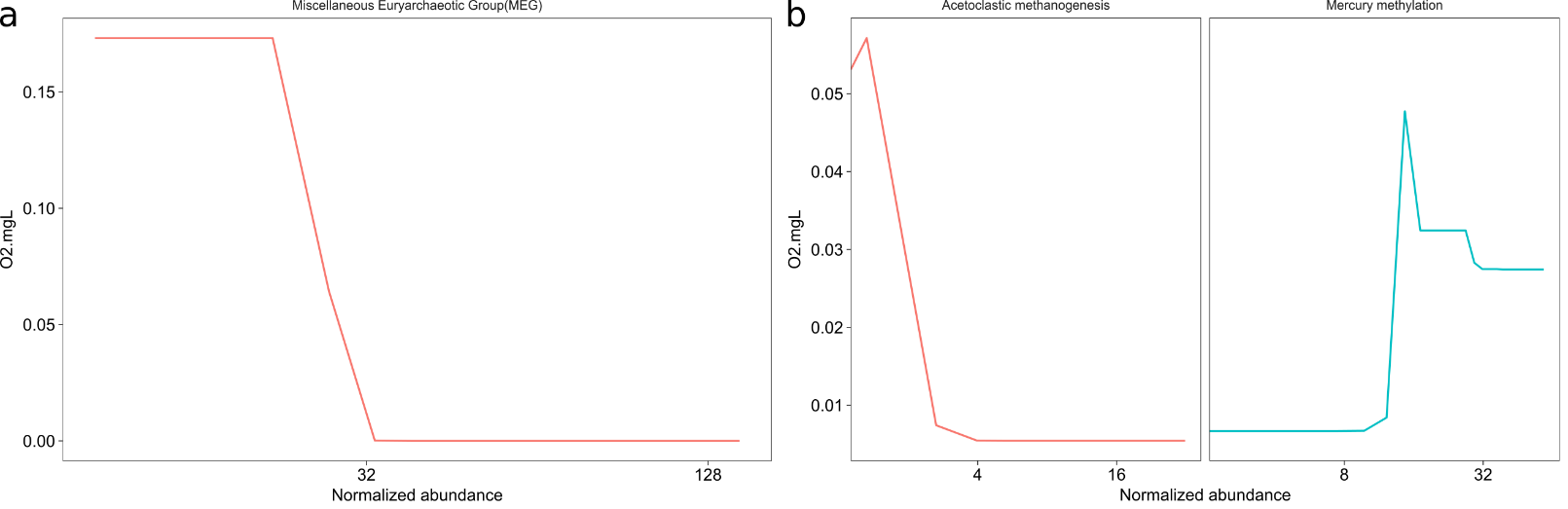


Figure S22: Partial dependence of random forest model prediction of [O2] from summer 2015 data set with archaeal primers. **a**: Phylogenetic data **b**: Functionally mapped data.


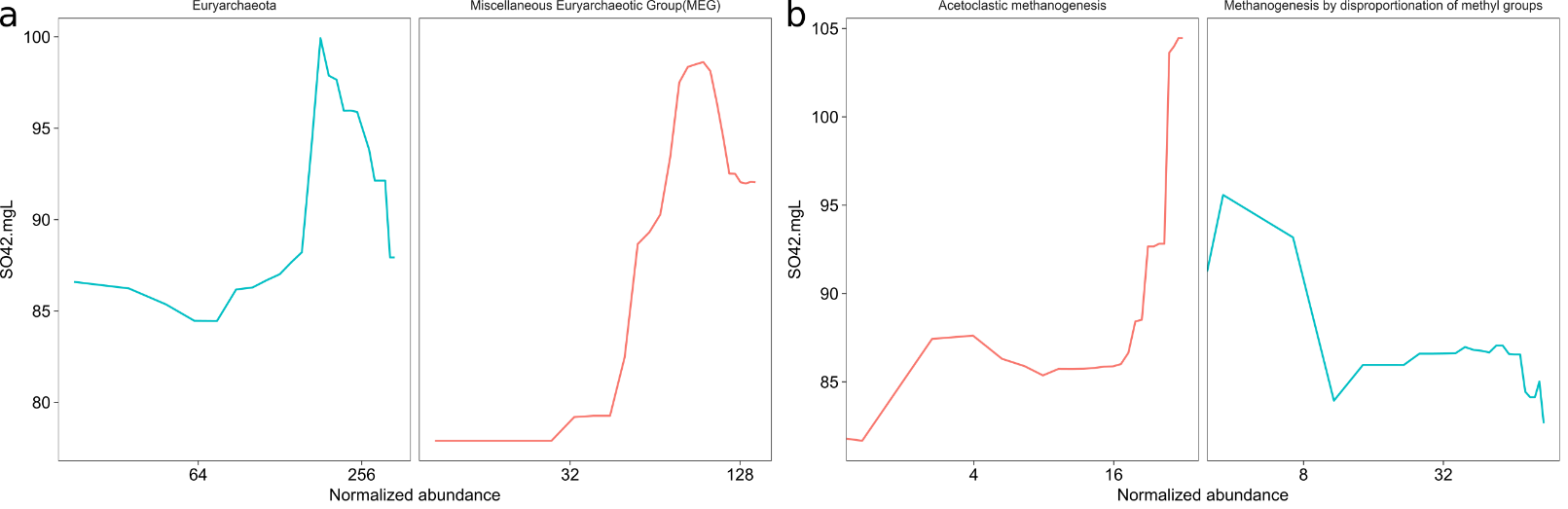


Figure S23: Partial dependence of random forest model prediction of [SO_4_^2-^] from summer 2015 data set with archaeal primers. **a**: Phylogenetic data **b**: Functionally mapped data.


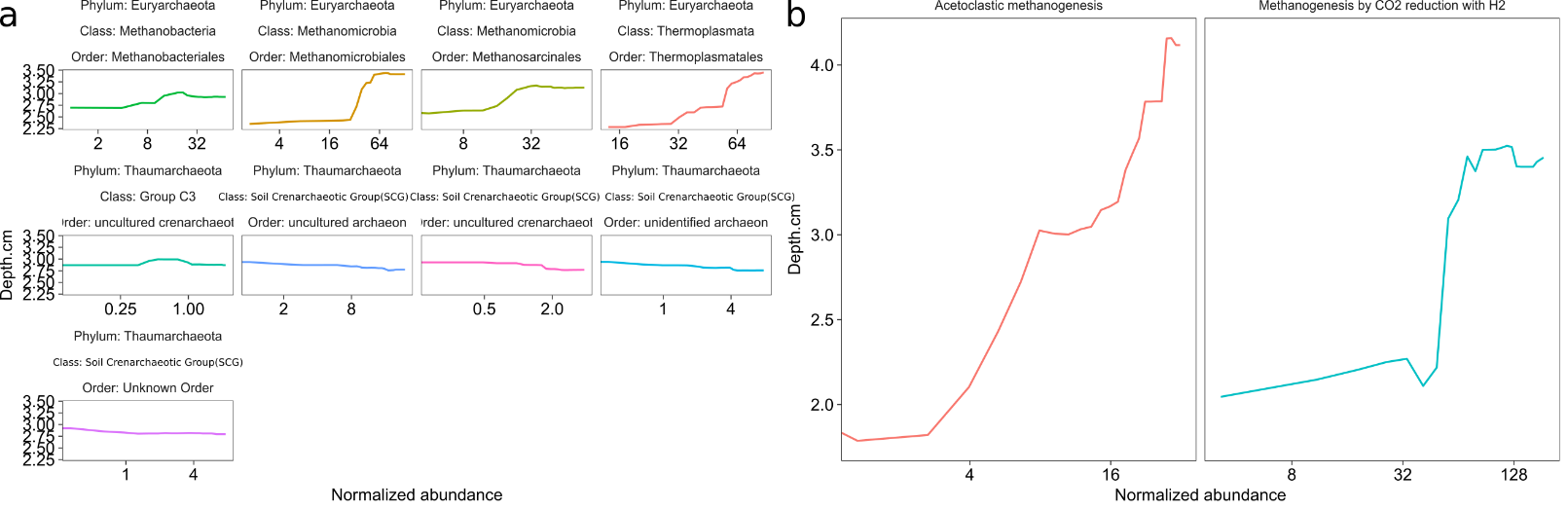


Figure S24: Partial dependence of random forest model prediction of sediment depth from summer 2015 data set with archaeal primers. **a**: Phylogenetic data **b**: Functionally mapped data.


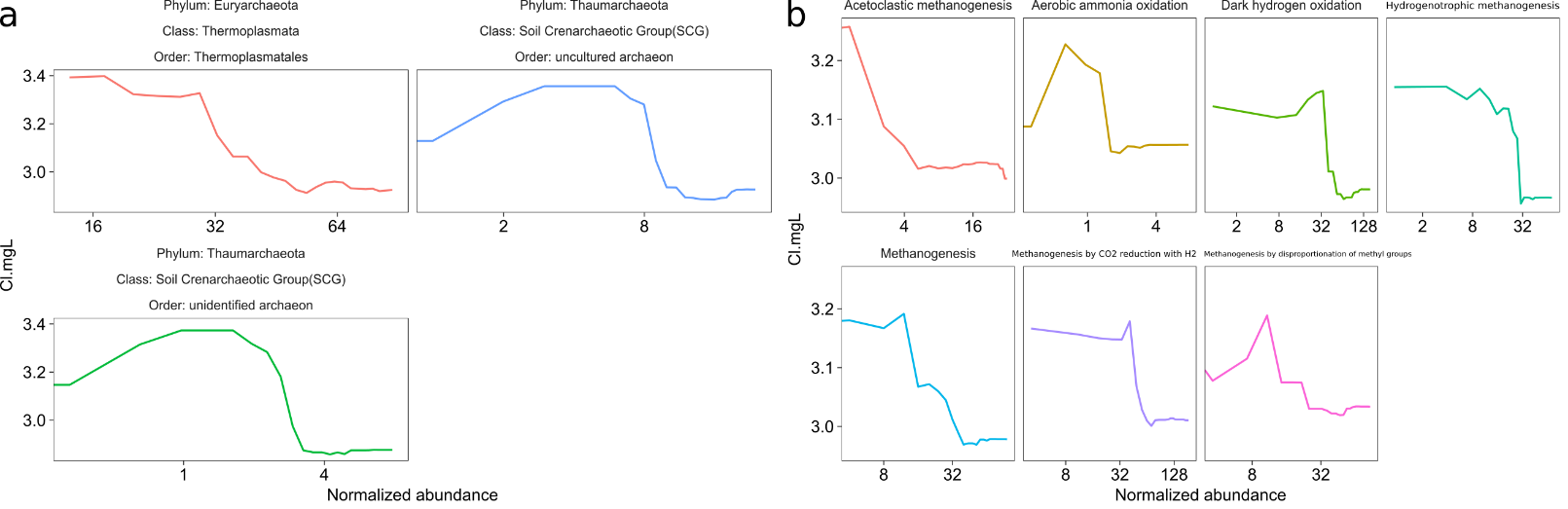


Figure S25: Partial dependence of random forest model prediction of [Cl-] from summer 2015 data set with archaeal primers. **a**: Phylogenetic data **b**: Functionally mapped data.


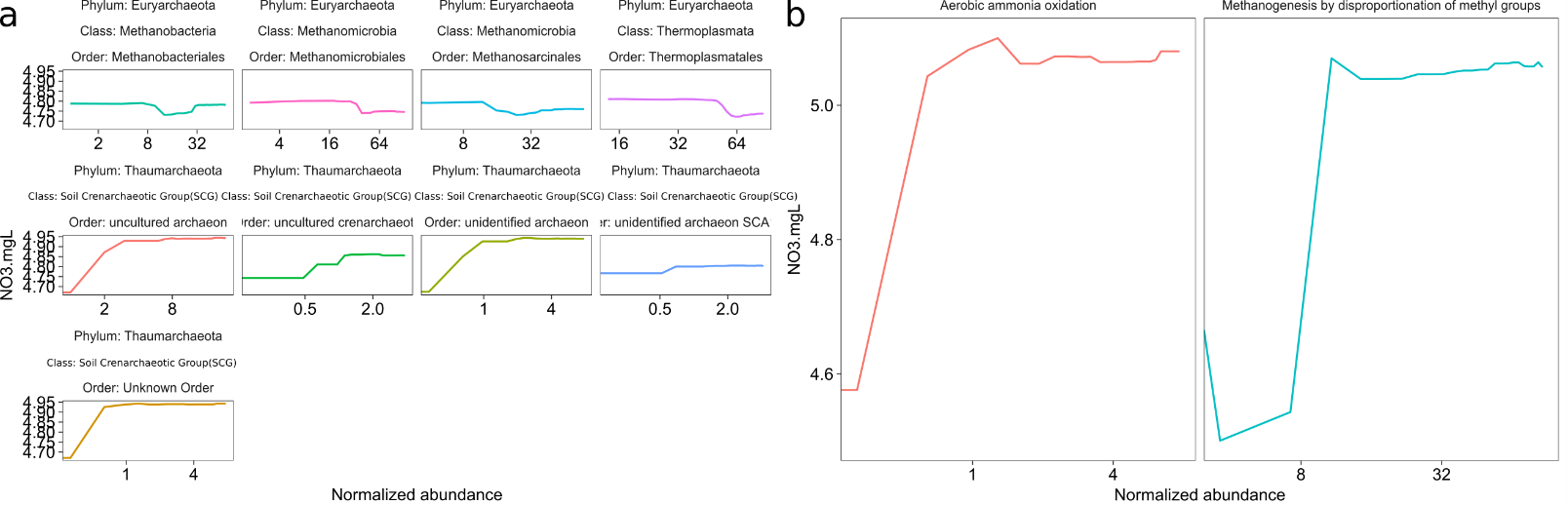


Figure S26: Partial dependence of random forest model prediction of [NO_3_-] from summer 2015 data set with archaeal primers. **a**: Phylogenetic data **b**: Functionally mapped data.


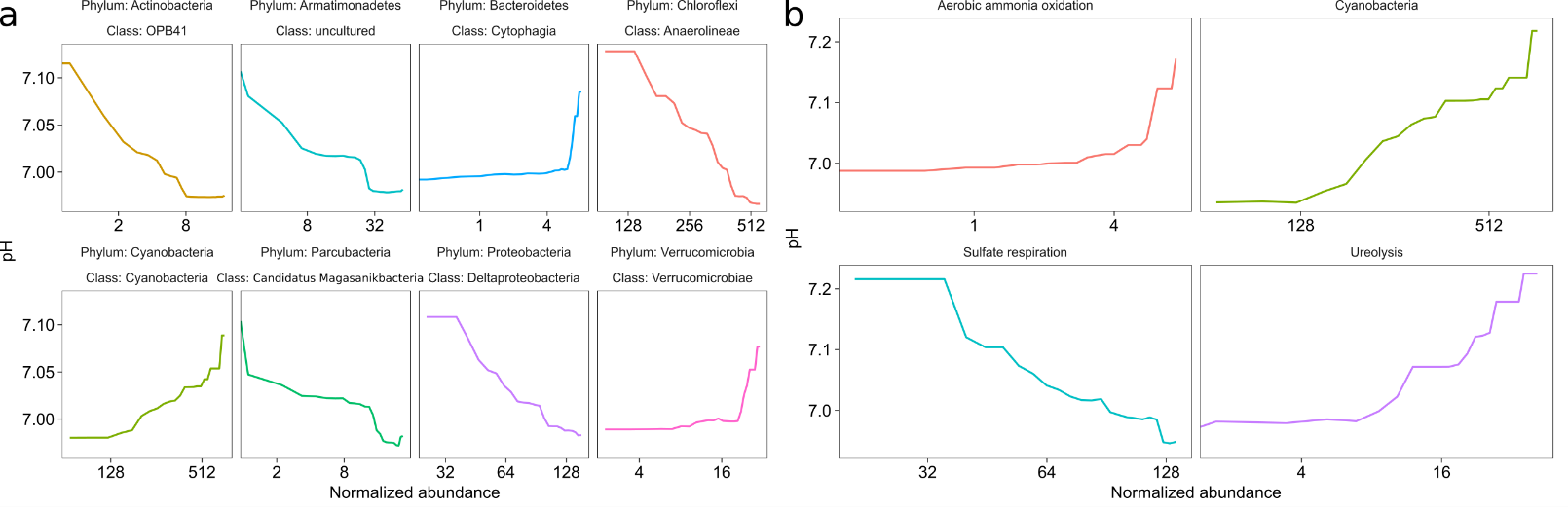


Figure S27: Partial dependence of random forest model prediction of pH from summer 2015 data set with bacterial primers. **a**: Phylogenetic data **b**: Functionally mapped data.


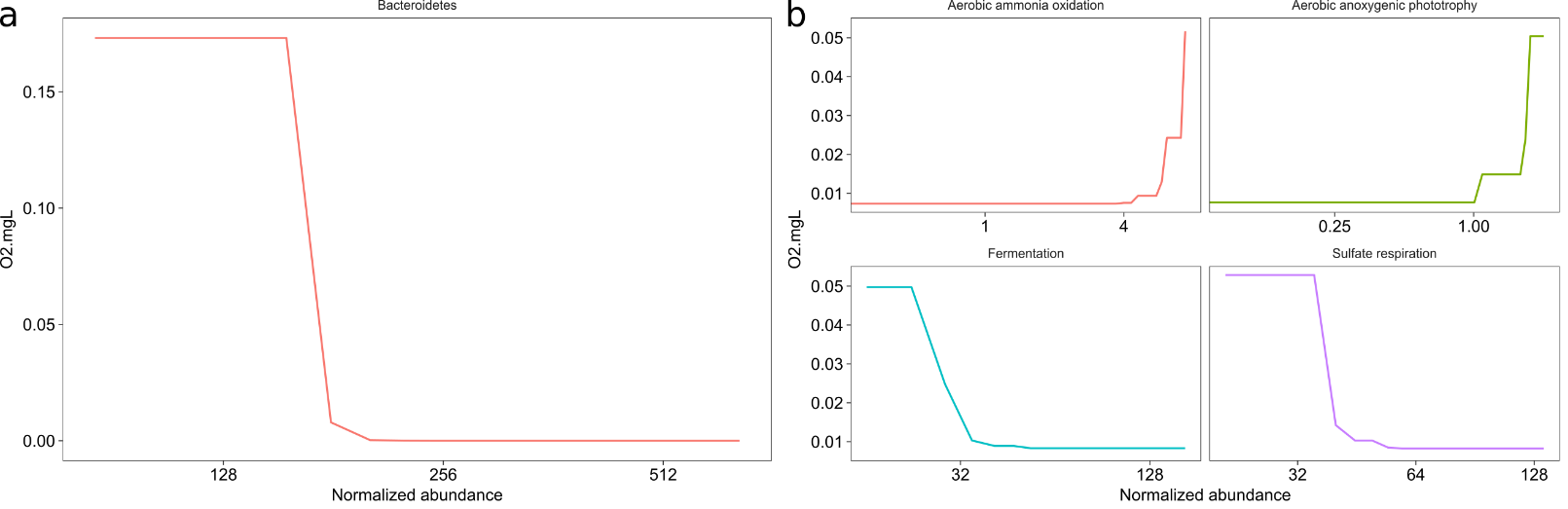


Figure S28: Partial dependence of random forest model prediction of [O_2_] from summer 2015 data set with bacterial primers. **a**: Phylogenetic data **b**: Functionally mapped data.


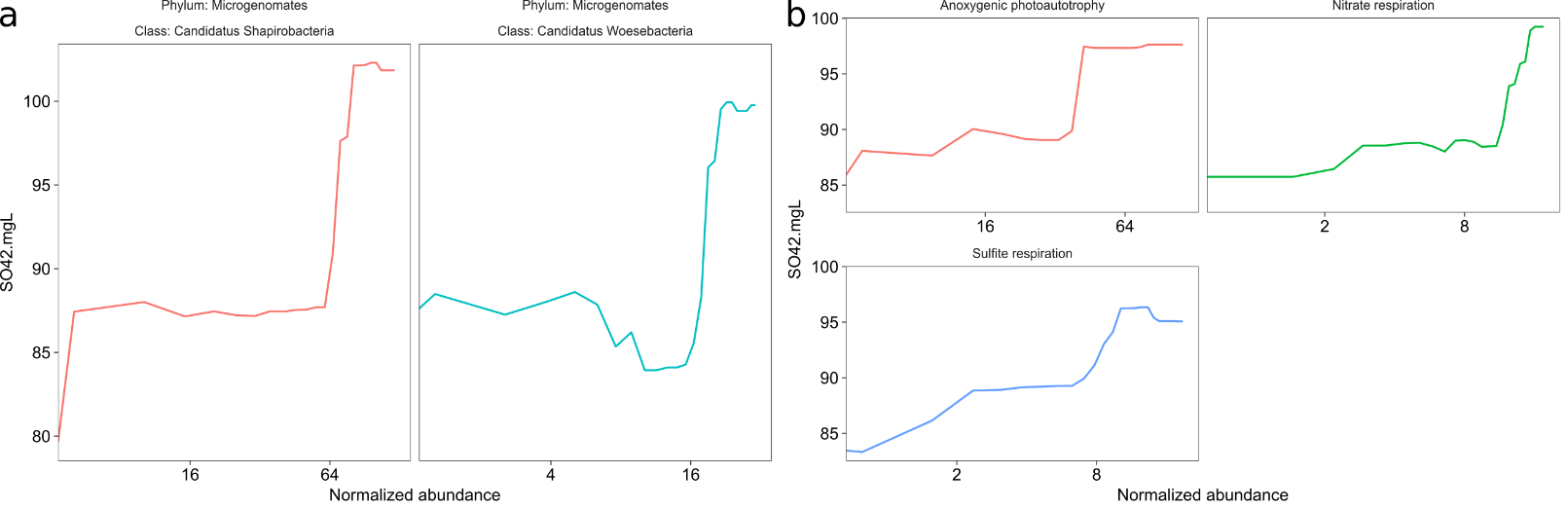


Figure S29: Partial dependence of random forest model prediction of [SO_4_^2-^] from summer 2015 data set with bacterial primers. **a**: Phylogenetic data **b**: Functionally mapped data.


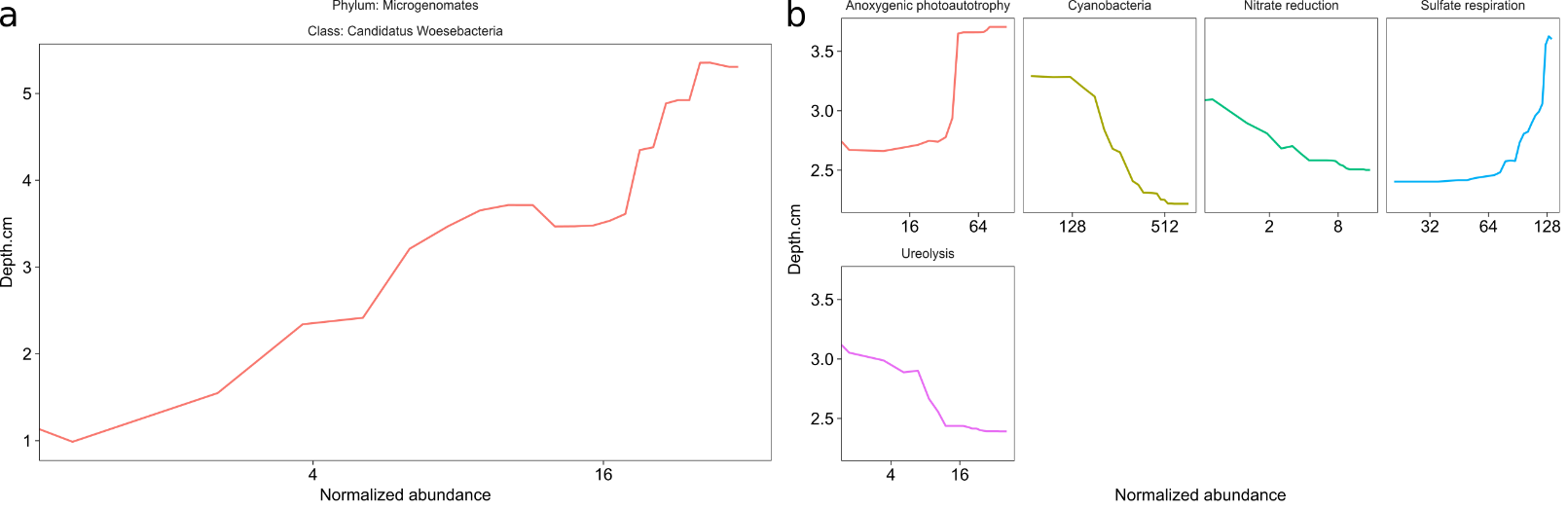


Figure S30: Partial dependence of random forest model prediction of sediment depth from summer 2015 data set with bacterial primers. **a**: Phylogenetic data **b**: Functionally mapped data.


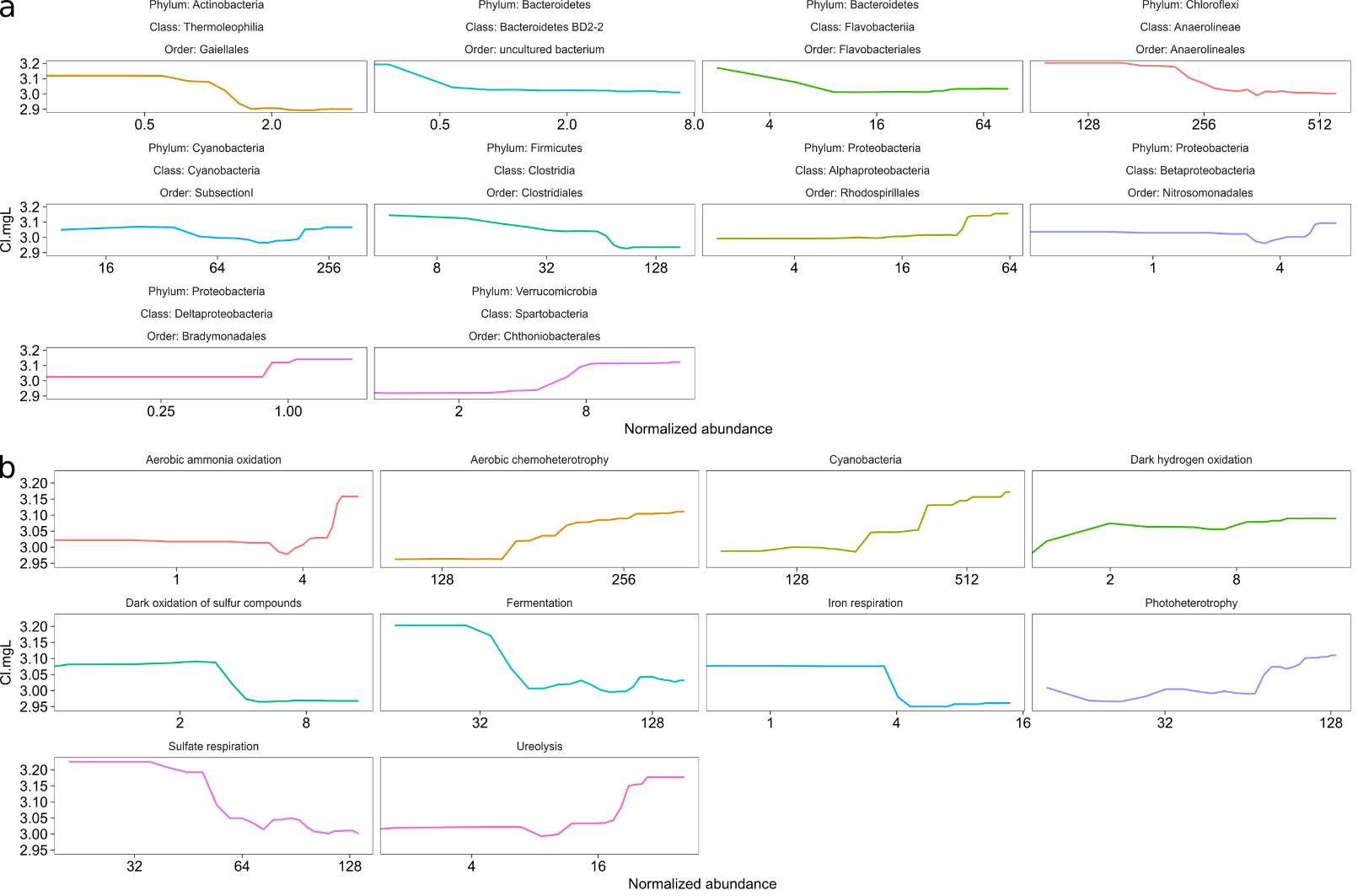


Figure S31: Partial dependence of random forest model prediction of [Cl-] from summer 2015 data set with bacterial primers. **a**: Phylogenetic data **b**: Functionally mapped data.


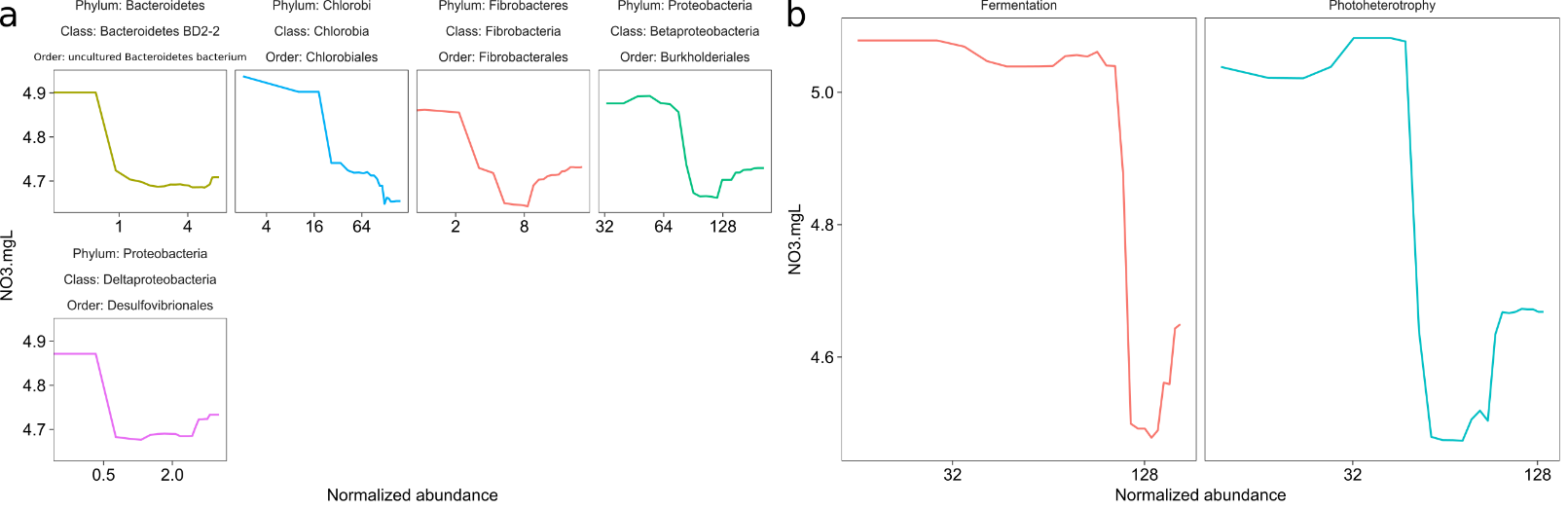


Figure S32: Partial dependence of random forest model prediction of [NO_3_-] from summer 2015 data set with bacterial primers. **a**: Phylogenetic data **b**: Functionally mapped data.

### 2.5.3 Categorical variables: spring 2014/2015


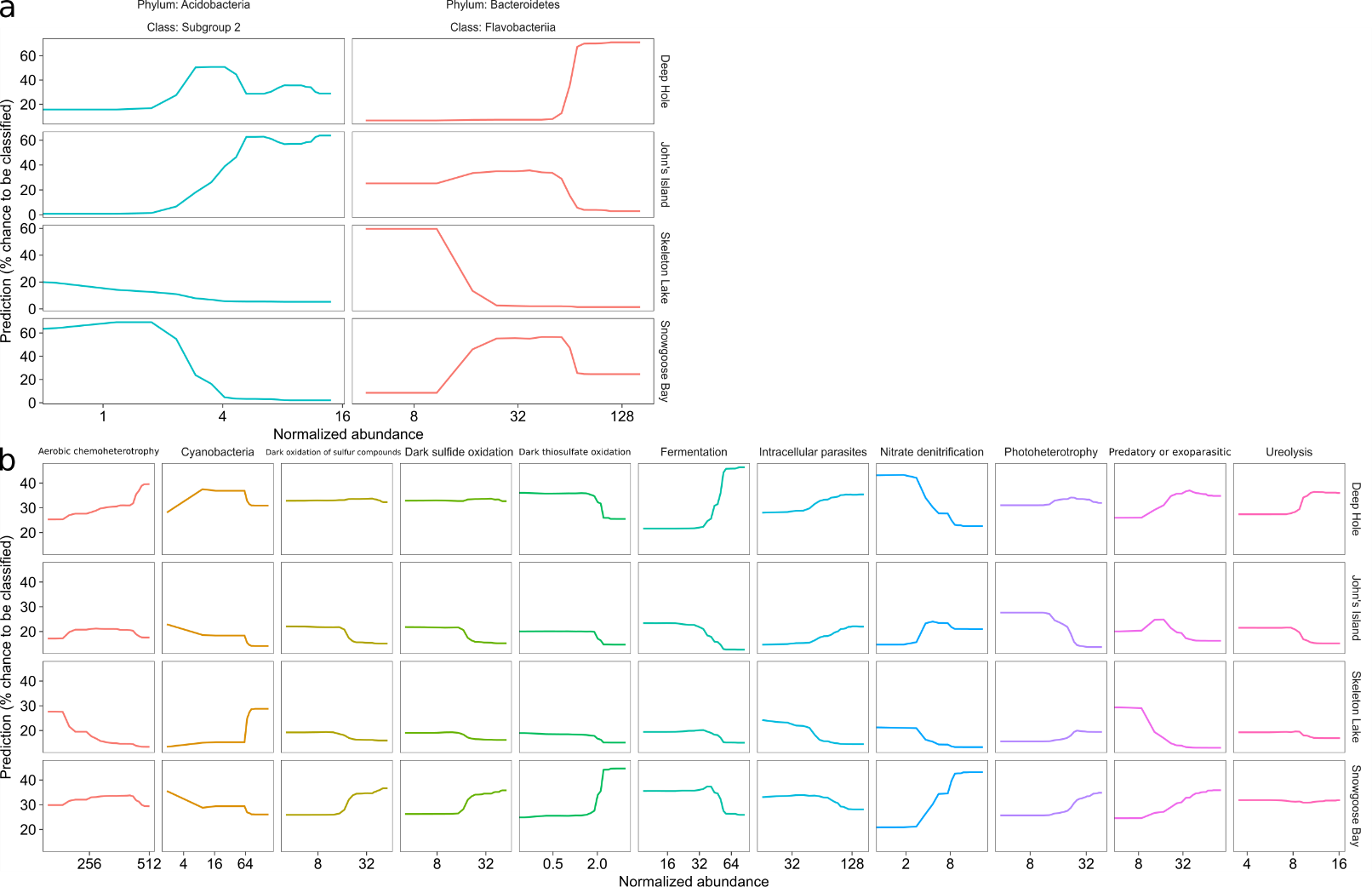


Figure S33: Partial dependence of random forest model prediction of sampling site from spring 2014/2015 data set with universal primers. **a**: Phylogenetic data **b**: Functionally mapped data.


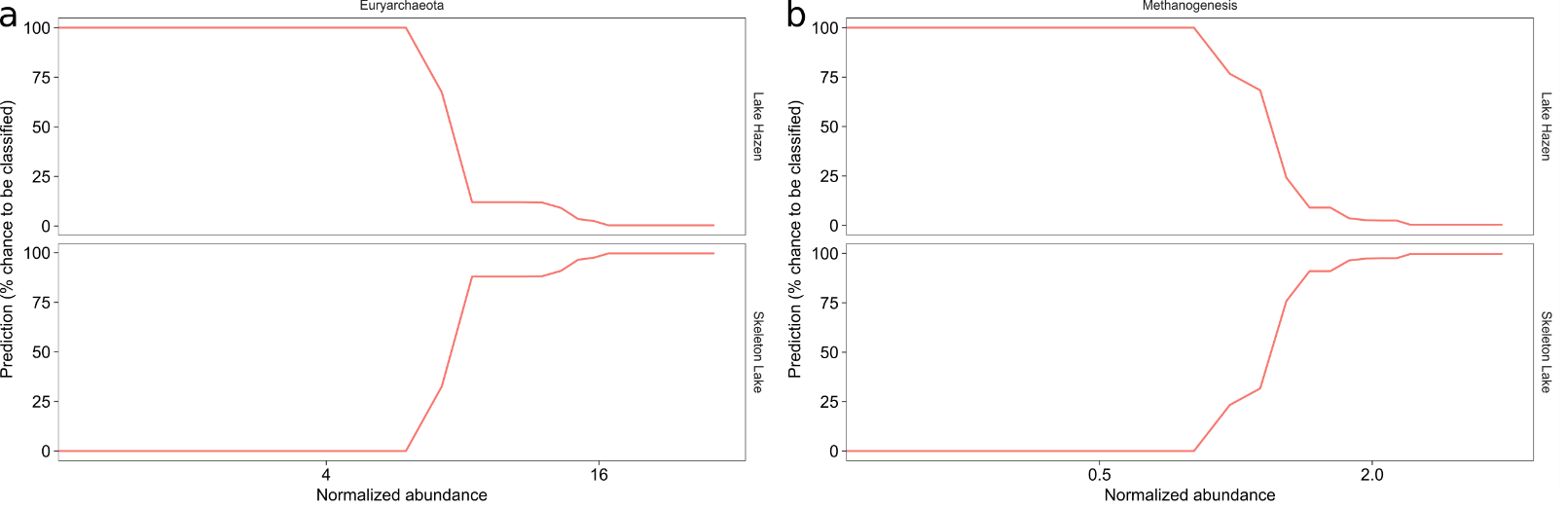


Figure S34: Partial dependence of random forest model prediction of sampled lake from spring 2014/2015 data set with universal primers. **a**: Phylogenetic data **b**: Functionally mapped data.


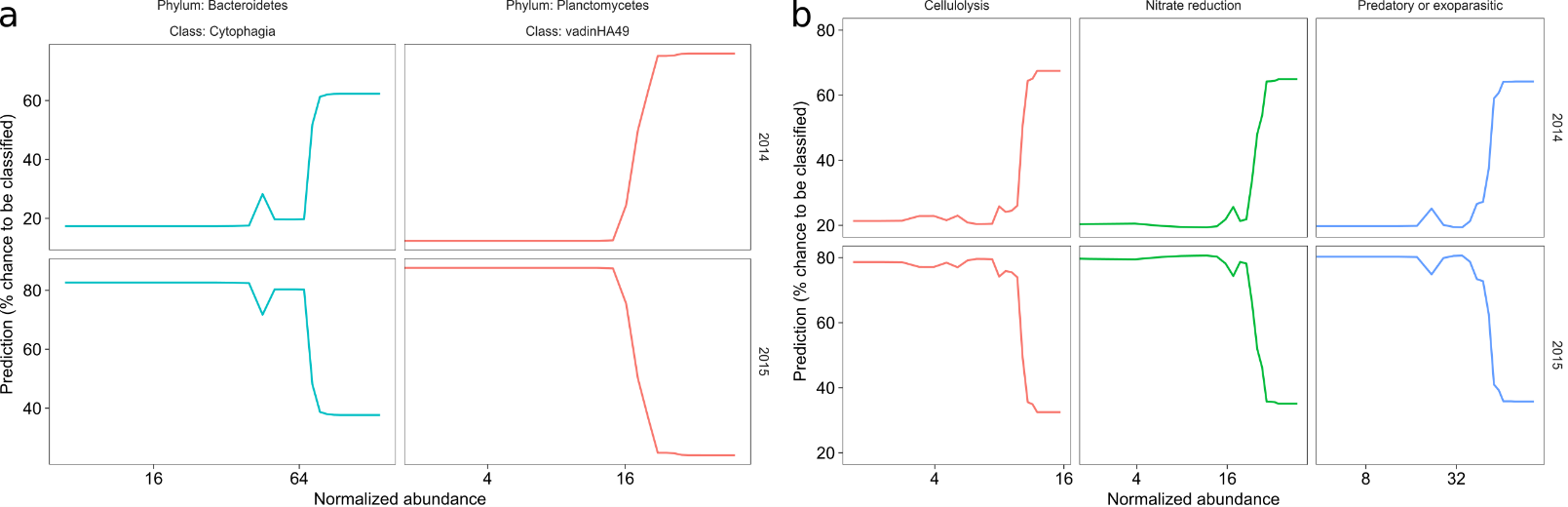


Figure S35: Partial dependence of random forest model prediction of sampling year from spring 2014/2015 data set with universal primers. **a**: Phylogenetic data **b**: Functionally mapped data.

### 2.5.4 Categorical variables: summer 2015


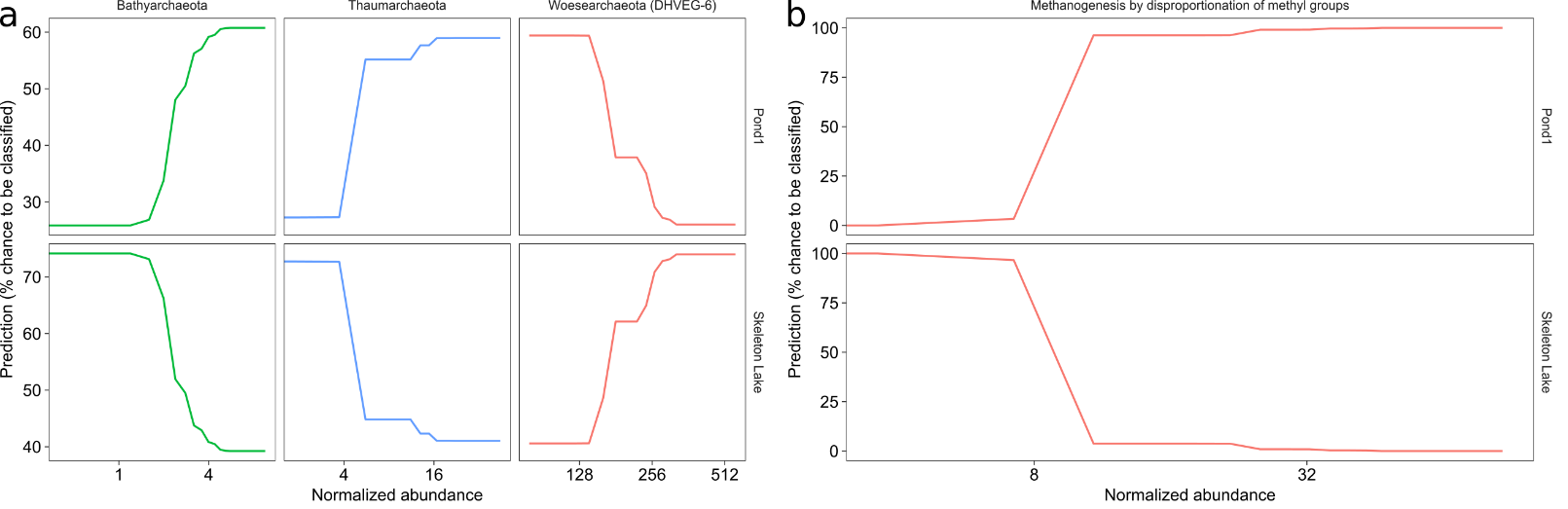


Figure S36: Partial dependence of random forest model prediction of sampling site from summer 2015 data set with archaeal primers. **a**: Phylogenetic data **b**: Functionally mapped data.


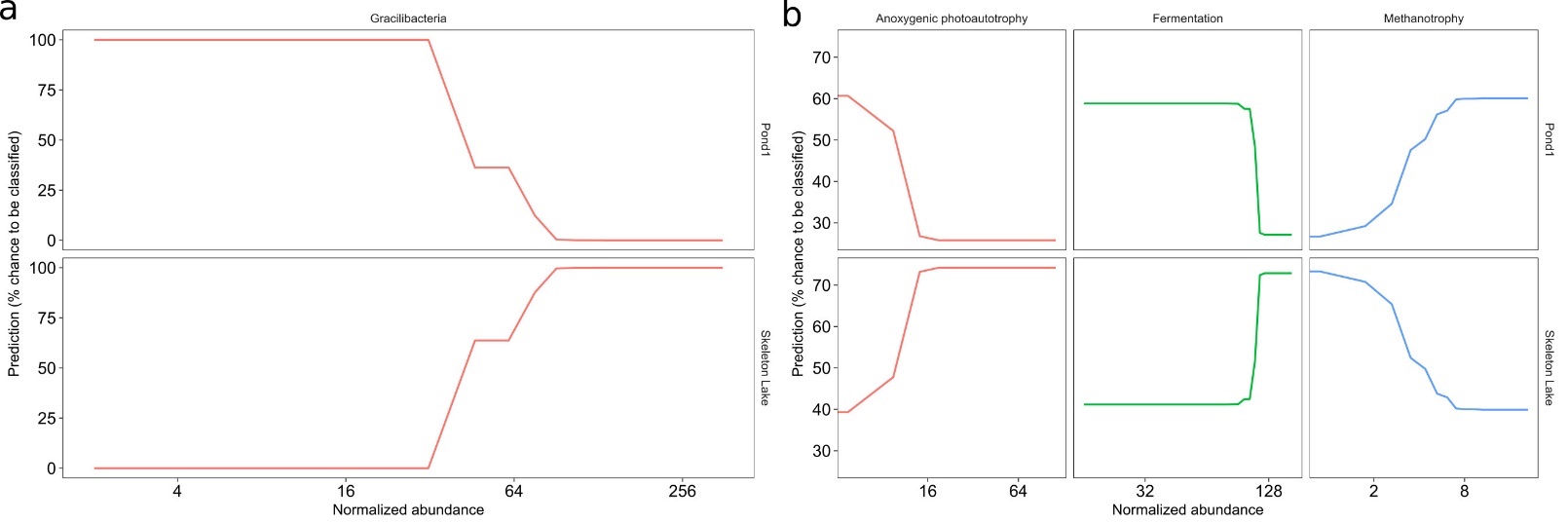


Figure S37: Partial dependence of random forest model prediction of sampling site from summer 2015 data set with bacterial primers. **a**: Phylogenetic data **b**: Functionally mapped data.

References

Andreotti, R., Pérez de León, A. A., Dowd, S. E., Guerrero, F. D., Bendele, K. G., and Scoles, G. A. (2011). Assessment of bacterial diversity in the cattle tick *Rhipicephalus* (*Boophilus*) microplus through tag-encoded pyrosequencing. *BMC Microbiol.* 11, 6. doi:10.1186/1471-2180-11-6.

Chu, H., Fierer, N., Lauber, C. L., Caporaso, J. G., Knight, R., and Grogan, P. (2010). Soil bacterial diversity in the Arctic is not fundamentally different from that found in other biomes. *Environ. Microbiol.* 12, 2998–3006. doi:10.1111/j.1462-2920.2010.02277.x.

Glassing, A., Dowd, S. E., Galandiuk, S., Davis, B., and Chiodini, R. J. (2016). Inherent bacterial DNA contamination of extraction and sequencing reagents may affect interpretation of microbiota in low bacterial biomass samples. *Gut Pathog.* 8. doi:10.1186/s13099-016-0103-7.

Herlemann, D. P., Labrenz, M., Jürgens, K., Bertilsson, S., Waniek, J. J., and Andersson, A. F. (2011). Transitions in bacterial communities along the 2000 km salinity gradient of the Baltic Sea. *ISME J.* 5, 1571–1579. doi:10.1038/ismej.2011.41.

Kämpfer, P. (2015). “*Sphingobacteriia class. nov.*,” in *Bergey’s Manual of Systematics of Archaea and Bacteria* (John Wiley & Sons, Ltd). doi:10.1002/9781118960608.cbm00013.

Klindworth, A., Pruesse, E., Schweer, T., Peplies, J., Quast, C., Horn, M., et al. (2013). Evaluation of general 16S ribosomal RNA gene PCR primers for classical and next-generation sequencing-based diversity studies. *Nucleic Acids Res.* 41, e1–e1. doi:10.1093/nar/gks808.

Köck, G., Muir, D., Yang, F., Wang, X., Talbot, C., Gantner, N., et al. (2012). Bathymetry and Sediment Geochemistry of Lake Hazen (Quttinirpaaq National Park, Ellesmere Island, Nunavut). *Arctic*, 56–66.

Rapp, J. Z., Bienhold, C., Offre, P., and Boetius, A. (2016). Polysaccharide degradation potential of bacterial communities in Arctic deep-sea sediments (1200-5500 m water depth). in Available at: http://epic.awi.de/41571/.

Salter, S. J., Cox, M. J., Turek, E. M., Calus, S. T., Cookson, W. O., Moffatt, M. F., et al. (2014). Reagent and laboratory contamination can critically impact sequence-based microbiome analyses. *BMC Biol.* 12, 87. doi:10.1186/s12915-014-0087-z.

Takai, K., and Horikoshi, K. (2000). Rapid detection and quantification of members of the archaeal community by quantitative PCR using fluorogenic probes. *Appl. Environ. Microbiol.* 66, 5066–5072.

Vergin, K. L., Urbach, E., Stein, J. L., DeLong, E. F., Lanoil, B. D., and Giovannoni, S. J. (1998). Screening of a fosmid library of marine environmental genomic DNA fragments reveals four clones related to members of the order Planctomycetales. *Appl. Environ. Microbiol.* 64, 3075–3078.

Ward, N. L., Challacombe, J. F., Janssen, P. H., Henrissat, B., Coutinho, P. M., Wu, M., et al. (2009). Three genomes from the phylum Acidobacteria provide insight into the lifestyles of these microorganisms in soils. *Appl. Environ. Microbiol.* 75, 2046–2056. doi:10.1128/AEM.02294-08.

Xiong, J., Liu, Y., Lin, X., Zhang, H., Zeng, J., Hou, J., et al. (2012). Geographic distance and pH drive bacterial distribution in alkaline lake sediments across Tibetan Plateau. *Environ. Microbiol.* 14, 2457–2466. doi:10.1111/j.1462-2920.2012.02799.x.
